# Survival and prognostic factors in hypertrophic cardiomyopathy: a meta-analysis

**DOI:** 10.1038/s41598-017-12289-4

**Published:** 2017-09-20

**Authors:** Qun Liu, Diandian Li, Alan E. Berger, Roger A. Johns, Li Gao

**Affiliations:** 1 Division of Allergy & Clinical Immunology, Johns Hopkins University School of Medicine, Baltimore, MD 21224 USA; 2 Department of Respiratory and Critical Care Medicine, West China Hospital, Sichuan University, Chengdu, Sichuan 610041 China; 3 Department of Anesthesiology and Critical Care Medicine, Johns Hopkins University School of Medicine, Baltimore, MD 21224 USA

## Abstract

Hypertrophic cardiomyopathy (HCM) is a clinically and genetically heterogeneous disorder but data on survival rates are still conflicting and have not so far been quantitatively reviewed. The aim of this study is to conduct a meta-analysis of cohort studies to assess pooled survival rates and prognostic factors for survival in patients with HCM. Nineteen studies were included representing 12,146 HCM patients. The pooled 1-, 3-, 5- and 10-year survival rates were 98.0%, 94.3%, 82.2% and 75.0%, respectively. Among patients with HCM, age, NYHA functional class, family history of sudden death (FHSD), syncope, atrial fibrillation, non-sustained ventricular tachycardia (nsVT), maximum left ventricular wall thickness and obstruction were significant prognostic factors for cardiovascular death. For sudden cardiac death, FHSD, nsVT, and obstruction showed significant predictive values. Moreover, estimation of population attributable risk (PAR) suggested that nsVT was the strongest predictor for cardiovascular death (13.02%, 95% CI 3.60–25.91%), while left ventricular outflow tract obstruction/mid-ventricular obstruction (LVO/MVO) was the strongest predictor for all-cause death and sudden cardiac death (10.09%, 95% CI 4.72–20.42% and 16.44%, 95% CI 7.45–31.55%, respectively). These risk factors may thus be useful for identifying HCM patients who might benefit from early diagnosis and therapeutic interventions.

## Introduction

Hypertrophic cardiomyopathy (HCM) is a clinically and genetically heterogeneous disorder. It is characterized most commonly by left ventricular hypertrophy (LVH), can lead to functional disability from heart failure (HF) and stroke^1^, and is the most frequent cause of sudden death in young people^2^. Recent studies suggest that HCM is more common than previously estimated (1/500), indeed, HCM would now appear to be the most common of the genetic heart diseases^3^.

Many studies have addressed survival and prognostic factors for survival in HCM^4–18^ and it has usually been asserted that survival among patients with HCM has improved over the years, with a range of potential outcomes including heart failure and sudden cardiac death, but also survival to normal life expectancy. In 1985, McKenna *et al*. reported a 2-year cumulative survival rate of 85.6%^19^; in 2003, Kawasaki *et al*. reported 2.3-year survival of 89% for HCM patients who were followed-up from 2001^17^; and more recently, 3-year survival of 90∼95% has been reported^7,11,20^. However, real improvement in the diagnosis and prognosis over time has not yet been clearly demonstrated^3^. Aside from the availability of new treatments and their possible impact, other factors explaining the observed variation in survival over time should be addressed, such as the baseline characteristics and average disease severity of patients, which may differ between studies^3^.

Prognostic factors for survival in HCM patients have also been assessed in several studies^1,2,4–7,9,11–13,17,19–33^ and were recently reviewed^18,28^. It is important to identify such factors in order to ensure optimal care and facilitate appropriate monitoring^4,8^. However, there are marked discrepancies among studies focused on HCM, and there is a lack of consensus on factors that best predict mortality in idiopathic HCM. Several studies have suggested that family history of sudden death (FHSD)^4,7,13,34^ and abnormal blood pressure response to exercise (aBPRE)^4,7,13,27^ are significant prognostic factors in HCM whereas other studies did not confirm these results^9,23^.

Similarly, there are major discrepancies concerning atrial fibrillation (AF) in HCM, in that some studies concluded that it was a prognostic factor^13,26,30^, whereas other studies indicated that it was not^9,16,20^. Conversely, in a recent review^18^, family history, aBPRE, and cardiac arrhythmia were recognized as important and indisputable prognostic factors for survival in idiopathic HCM. One explanation for this could be insufficient power in some studies of HCM to accurately identify prognostic factors. Indeed, there is a great need for studies of HCM that focus on prognostic factors and the effect of these risk factors in the overall population.

In the current study, we conducted a systematic review and meta-analysis of cohort studies to assess pooled survival rates and prognostic factors for survival in HCM. We also investigated the correlation between survival and the baseline clinical features of HCM and between survival and the period of time during which patients were included in the studies (time of inclusion). A key secondary objective was to estimate the population attributable risks (PAR) for risk factors in the overall population.

## Results

### Selection of the studies

**Figure 1:**
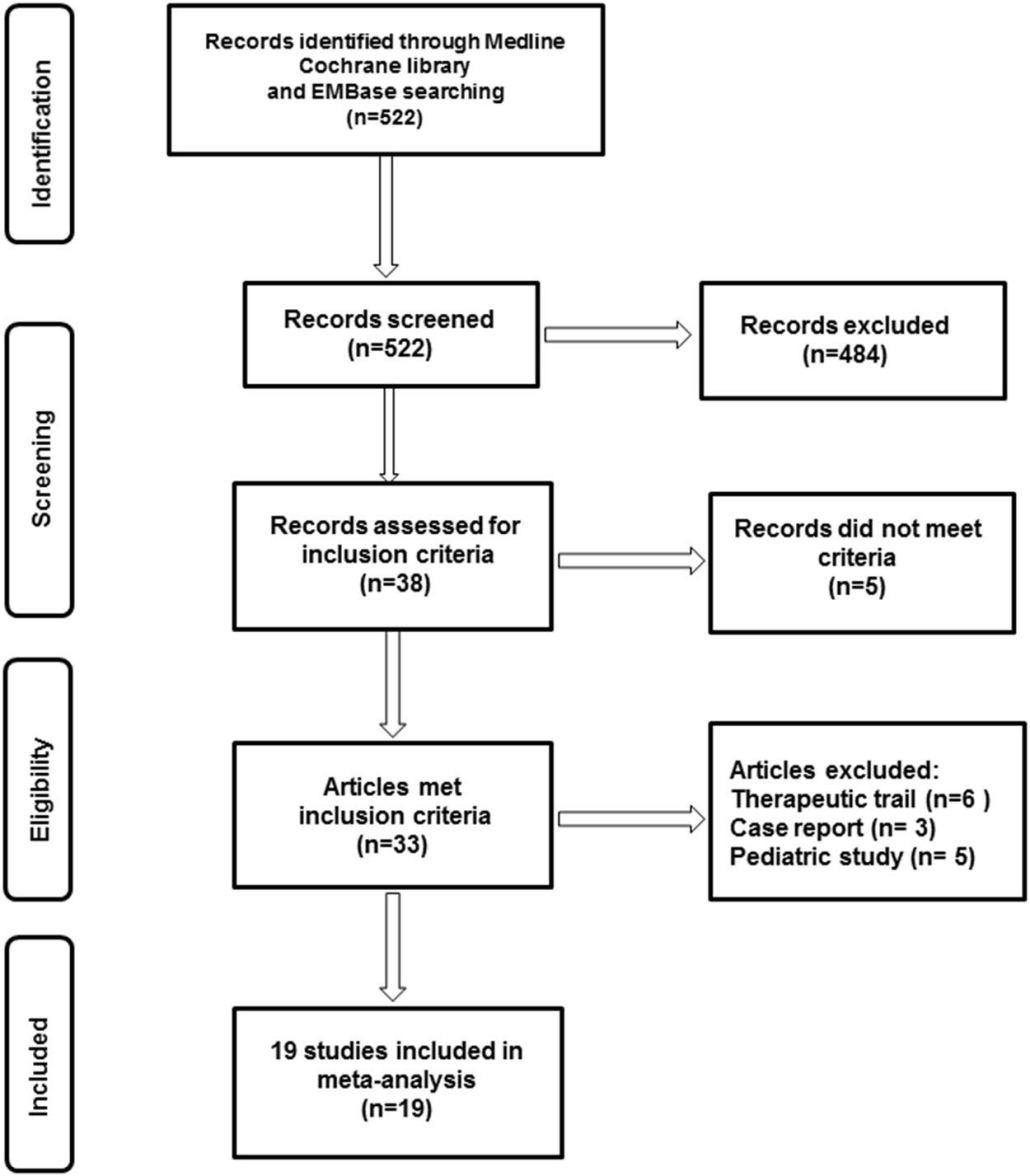
Flow diagram showing the search strategies used to identify publications for inclusion in the study.

Our search of the Medline, Cochrane database and EMBase databases for the period from January 1960 to September 2015 resulted in 522 citations (*see* Supplementary Results). After the abstracts were evaluated, 33 studies were examined for full text, and after the exclusion criteria described below were applied, 19 articles were finally selected, which included 12,146 patients distributed in 9 countries (Fig. 1 and Table 1). The HCM diagnoses of all cases were verified by echocardiography measures or clinical data.

**Table 1:** Baseline characteristics of patients included in the selected studies*.

Author	No. of NOS stars	Publication date	Sample size	Ethnicity	Male n (%)	Average age (years) (mean ± SD)	Follow-up period (years) (mean ± SD)	FHSD n (%)	NYHA class III/IV n (%)	Synocope n (%)	aBPRE n (%)	Af n (%)	nsVT n (%)	LAD (mm) (mean ± SD)	Obstruction (LVO/MVO) n (%)
Olivotto *et al*.	7	1999	126	Italy	90 (71.4%)	42 ± 14	4.7 ± 3.7	14 (11%)		16 (13%)	28 (22.2%)	14 (11%)	49 (39%)		27 (21%)
Olivotto *et al*.	7	2001	480	Italian	292 (60.8%)	45 ± 20	9.1 ± 6.4		54 (51%)	9 (8%)		25 (23%)	42 (39%)	50 ± 9	34 (32%)
Elliott *et al*.	7	2001	630	UK	382 (60.6%)	37 ± 16	10.42	162 (26%)	24 (4%)	108 (17%)	143 (23%)		101 (16%)	42 ± 9	146 (23%)
Monserrat *et al*.	7	2003	531	UK	323 (60.8%)	39 ± 15	5.83 ± 3.33	137 (26%)	19 (4%)	97 (18%)	155 (29%)	21 (4%)	104 (19.6%)	42.6 ± 8.3	120 (22%)
Maron *et al*.	7	2003	1101	Italy/USA	655 (59.0%)	45 ± 20	6.3 ± 6.2		107 (10)	152 (14)					273 (25%)
Arteaga *et al*.	6	2005	214	Brazil	102 (47.7%)	37 ± 16	1980–1997	28 (13%)	21 (10%)	33 (15%)		18 (8%)	33 (19%)		81 (51%)
Biagini *et al*.	6	2005	222	Italian	144 (64.9%)	40 ± 20	11 ± 9	47 (21%)	22 (10%)	21 (9%)		3 (1%)			67 (30%)
Nistri *et al*.	8	2006	1491	Italian	917 (61.5%)	47 ± 17	9.4 ± 7.4	516 (35%)	174 (12%)	134 (9%)		252 (17%)		43 ± 9	338 (23%)
Nasermoaddeli *et al*.	6	2007	1605	Japan	1118 (69.7%)	58.0 ± 17.5	5		76 (6.8%)			113 (7.7%)			
Losi *et al*.	8	2009	140	Italian	87 (62.1%)	40 ± 15	5 ± 3								
Yang *et al*.	8	2009	81	Korea	51 (63.0%)	57 ± 14	3.42 ± 1.42		17 (21%)	10 (12%)					23 (28%)
Gimeno *et al*.	6	2009	1380	UK	855 (62.0%)	42 ± 15	4.5 ± 4.1			194 (14.1%)	298 (21.6%)	124 (8.9%)	243 (17.6%)		365 (26.4%)
Spirito *et al*.	6	2009	1511	Italy	927 (61.4%)	46 ± 19.7	5.6 ± 5.2	288 (19%)	168 (11%)	205 (13.6%)				43 ± 8.5	442 (29%)
Dimitrow *et al*.	5	2010	1306	Poland	705 (54.0%)	47.5	5.6 ± 4.3	274 (20.9%)		366 (28.0%)	418 (32.0%)		353 (27.0%)		
Finocchiaro *et al*.	8	2012	84	Italy	52 (61.9%)	48 ± 17	8.5	17 (20%)	9 (10%)			11 (13%)	16 (21%)	43 ± 10	
Efthimiadis *et al*.	7	2013	423	Greece	280 (66.2%)	49.3 ± 17.2	7	46 (10.9%)	70 (16.5%)	58 (13.7%)	57 (13.5%)	95 (22.5%)	43 (10.2%)	41 ± 7.1	122 (28.8%)
Klarich *et al*.	6	2013	193	USA	120 (62.0%)	58 ± 17	4.1 ± 3.7	19 (10%)	34 (18%)	22 (11%)		43 (22%)	21 (11%)	43 ± 9	
Wang *et al*.	7	2014	529	Chinese	368 (69.6%)	50.4 ± 14.4	4.7 ± 3.2	77 (14.6%)	65 (12.3%)	59 (11.2%)		68 (12.9%)	8 (1.5%)		186 (35.2%)
Xiao *et al*.	7	2015	99	Chinese	71 (71.7%)	52 ± 16	3.9 ± 3	27 (27%)	56 (57%)	27 (27%)		53 (54%)	48 (48%)	48 ± 9	22 (22%)

*NOS = Newcastle–Ottawa Scale; HCM = hypertrophic cardiomyopathy; NYHA = New York Heart Association; FHSD = family history of SD; nsVT = non-sustained ventricular tachycardia; aBPRE = abnormal blood pressure response to exercise; LAD = left atrial diameter; LVOTO = left ventricular outflow tract obstruction; MVO = mid-ventricular obstruction; Af = Atrial fibrillation; Blank = Data not available.

### Survival of HCM patients

**Figure 2:**
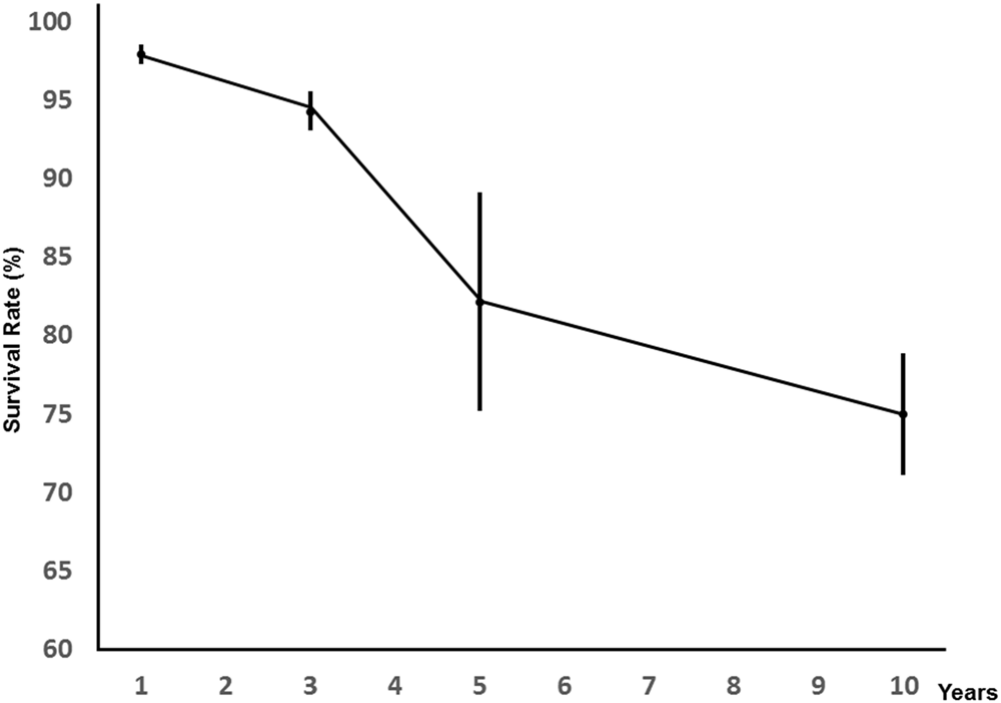
Pooled survival rate of recruited studies. The vertical lines represent 95% confidence intervals.

The pooled 1-, 3-, and 5-year survival rates were 98.0% (95% CI 97.4–98.6%), 94.3% (95% CI 93.1–95.6%) and 82.2% (95% CI 75.2–89.2%), respectively (Fig. 2). One main study inclusion criteria for consideration in the meta-analysis was the reporting of 1, 3 and 5 years HCM survival statistics. Although only 4 out of 19 studies that met our selection criteria have provided average ~10 years follow-up data, we think they are valuable, therefore we have performed additional meta-analysis on the pooled 10-year HCM survival rate. Our meta-analysis found a less favorable pooled 10-year survival of 75.5% (95% CI 71.1–78.9%). Tests for heterogeneity revealed significant heterogeneity of survival rates at 1, 3, 5 and 10 years. We speculate that variations in the time of the cohort studies, baseline electrocardiography and echocardiography measures, blood pressure response to exercise, and average age of HCM patients might explain this heterogeneity.

A meta-regression analysis was performed to explore the possible reasons for the heterogeneity of survival rates based on the following variables with sufficient data: time (mid–cohort year, publication date (before 2005 or after 2005)), sample size (≥1000 or <1000), geographical location (America, Europe or Asia) and Newcastle-Ottawa Scale (NOS) scores (≥7 or <7). We found that none of the above covariates was a statistically significant source of heterogeneity (all *p* > 0.05) (Table 2).

**Table 2:** Meta-regression of potential heterogeneity within the included studies.

Covariates	Number of studies (Total = 19)	Coefficient	SE	P-value
**Time**				
mid–cohort year		0.0462524	0.0263054	0.098
>5 years	11			
≤5 years	8			
publication date		−0.0020505	0.0030707	0.514
before 2005	7			
after 2005	12			
Sample size		0.0000133	0.0000252	0.604
≥1000	6			
<1000	13			
Geographical location				
America	2	−0.0358219	0.0334457	0.300
Europe	13			
Asia	4			
NOS scores				
≥7	12	−0.0050001	0.0176296	0.780
<7	7			

### Publication bias evaluation

**Figure 3:**
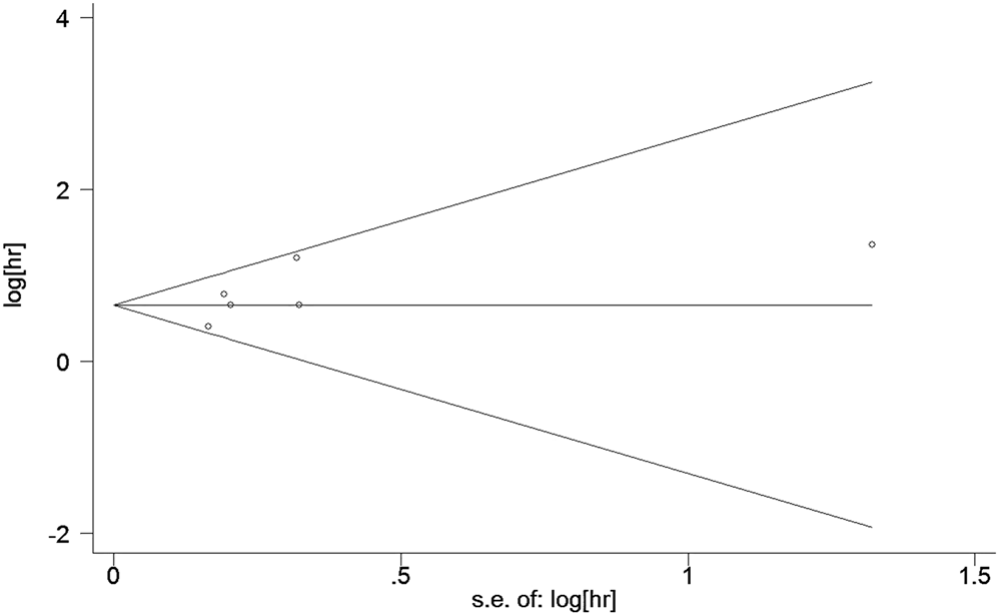
Begg’s funnel plot to assess the degree of publication bias. Circles in black color represent the six original studies included in the meta-analysis. Eggers test for family history of sudden cardiac death was not significant (p = 0.095). HR: hazard ratio. *We endeavored to conduct a comprehensive analysis, nevertheless, none of these variables were studied by all selected articles, therefore we have selected and focused on FH, which has been reported consistently among these studies, 6 studies showed follow-up data for FH.

Publication bias was examined using Eggers test with Begg’s funnel plots for FHSD, which was present in most of the 19 studies included in the meta-analysis. The shape of the funnel plot of the pooled HR did not reveal any evidence of obvious asymmetry (Fig. 3). The Eggers test also showed a statistically non-significant value (*p* = 0.095), indicating that there was no statistically significant publication bias. Begg’s funnel plot for NYHA class III/IV of cardiovascular death was presented in Supplementary Figure 7 (*p* = 0.093). In addition, we conducted the combined effect analysis to estimate the effect of each single study (Supplementary Figures 4, 5 and 6).

### Prognostic factors for survival

**Figure 4:**
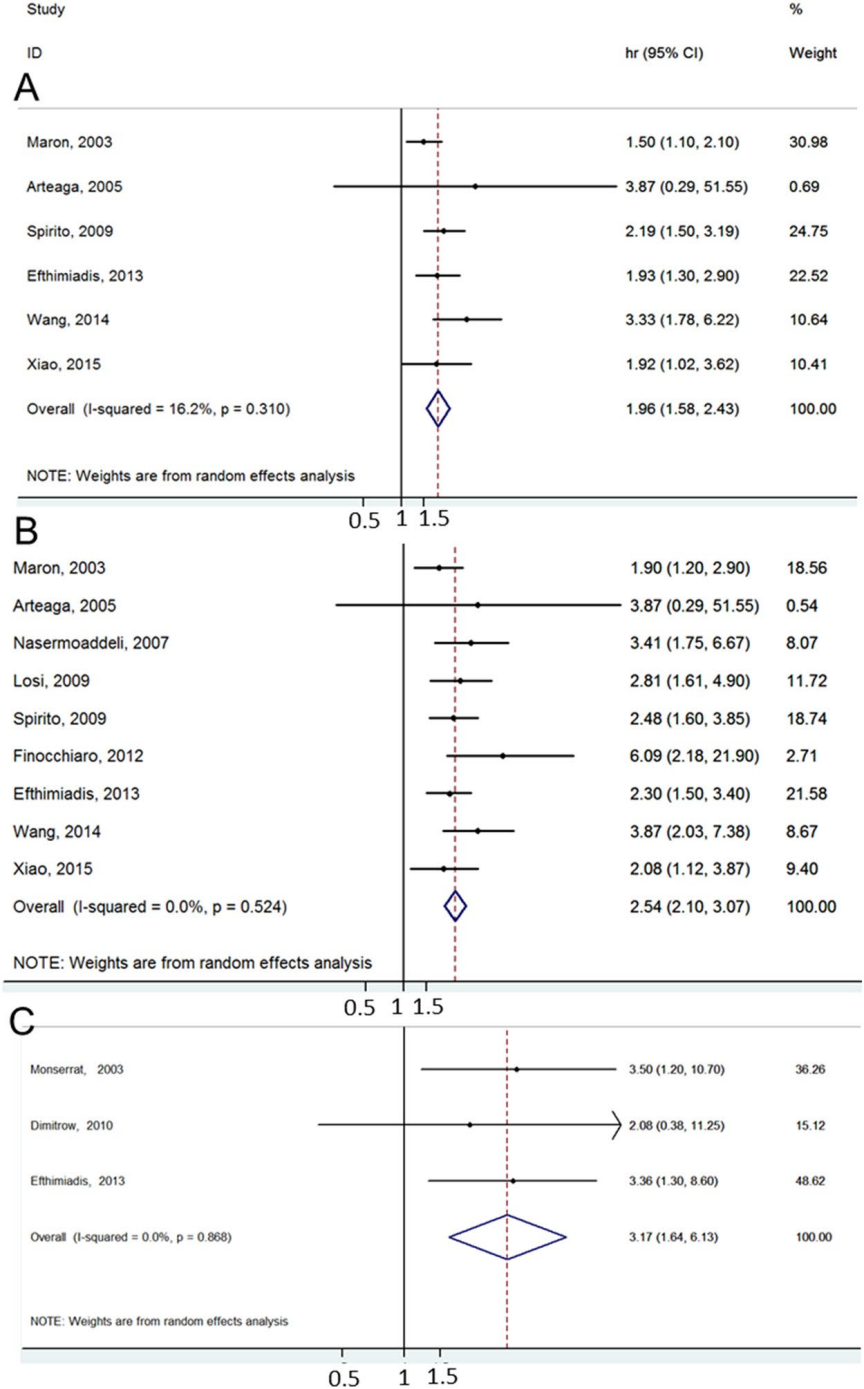
Forest plots showing the prognostic value of NYHA and MLVWT, represented by the pooled hazard ratio (HR) in HCM. The diamonds represent the combined HRs for each of the prognostic factors. (**A**) NYHA class III/IV is the strongest prognostic factor for all-cause death (HR = 1.96; 95% CI (1.58, 2.43)); (**B**) NYHA class III/IV is the most important risk factor for cardiovascular death (HR = 2.54; 95% CI (2.10, 3.07)); (**C**) MLVWT is the most important risk factor for sudden cardiac death (HR = 3.17; 95% CI (1.64, 6.13)).

The prognostic factors for survival and pooled hazard ratios are summarized in Table 3. Among patients with HCM, average age, NYHA functional class, non-sustained ventricular tachycardia (nsVT, Supplementary Figure 1), family history of sudden death (Supplementary Figure 2), syncope, AF, maximum left ventricular wall thickness (MLVWT) and obstruction (LVO/MVO) were significant prognostic factors for cardiovascular death. Conversely, for all-cause death, average age and FHSD were not significant prognostic factors. For sudden cardiac death (SCD), nsVT, FHSD (Supplementary Figure 3), and obstruction showed significant predictive values. Furthermore, NYHA class III/IV was the most important risk factor for cardiovascular death (HR = 2.53, 95% CI (2.10, 3.07)); MLVWT was the most important risk factor for sudden cardiac death (HR = 3.17, 95% CI (1.64, 6.12)) whereas NYHA class III/IV showed the strongest prognostic value for all-cause death (HR = 1.96, 95% CI (1.58, 2.43)) (Fig. 4).

**Table 3:** Prognostic factors and PAR for all-cause death, cardiovascular death and sudden cardiac death in HCM patients.

Prognostic factors	Prevalence (%)	All-cause death		Cardiovascular death		Sudden cardiac death	
		HR (95% CI)	PAR (%)	HR (95% CI)	PAR (%)	HR (95% CI)	PAR (%)
		Number of studies	(95% CL)	Number of studies	(95% CL)	Number of studies	(95% CL)
Age, years		1.05 (0.99, 1.09)		1.05 (1.01, 1.09)		0.53 (0.18, 1.535)	
		2		8		2	
Male sex	62.5	0.95 (0.49, 1.87)		0.78 (0.54, 1.12)			
		3		8			
FHSD	19.5	2.19 (0.38, 12.60)		2.38 (1.21, 4.67)	**11.31**	2.34 (1.46, 3.75)	**11.89**
		7		7	**(1.74, 24.99)**	6	**(3.15, 23.32)**
NYHA class III/IV	15.9	1.96 (1.58, 2.43)	**7.79**	2.54 (2.10, 3.07)	**9.61**		
		6	**(1.46, 31.42)**	9	**(2.10, 36.55)**		
Syncope	14.4	1.40 (1.02, 1.93)	4.11	2.39 (1.66, 3.44)	**8.37**	2.31 (1.22, 4.38)	**8.16**
		3	(0.15, 13.15)	4	**(3.18, 19.36)**	3	**(1.44, 21.07)**
aBPRE	23.6	0.88 (0.44, 1.74)		1.59 (0.83, 3.07)		1.38 (0.65, 2.89)	
		2		4		4	
Af	14.7	1.60 (0.93, 2.75)		1.52 (1.07, 2.17)	5.03		
		5		7	(0.07, 22.43)		
nsVT	22			2.46 (1.69, 3.57)	**13.02**	2.92 (1.97, 4.33)	14.47
				4	**(3.60, 25.91)**	4	(0.74, 34.84)
LAD	28.1	1.04 (0.99, 1.09)		1.07 (0.98, 1.17)			
		4		5			
MLVWT		1.48 (1.01, 2.17)		1.42 (1.06, 1.89)		3.17 (1.64, 6.13)	
				6		3	
LVEF				1.44 (0.97, 2.13)			
				3			
Obstruction (LVO/MVO)	28.1	1.56 (1.29, 1.90)	**10.09**	1.52 (1.11, 2.07)	**9.61**	2.41 (1.55, 3.73)	**16.44**
		4	**(4.72, 20.42)**	6	**(2.08, 22.28)**	3	**(7.45, 31.55)**

^*^Values are the pooled hazard ratio (95% confidence interval). PAR = population attributable risk; MLVWT = maximum left ventricular wall thickness; LVEF = LV ejection fraction; see Table 1 for other definitions. Significant PAR values are highlighted in bold.

### Population attributable risks

Table 3 also showed PARs by clinical outcomes for the various risk factors according to pooled hazard ratios. Family history of sudden death accounted for 11.31% (95% CI 1.74–24.99%) and 11.89% (95% CI 3.15–23.32%) of the PAR for cardiovascular death and sudden cardiac death, whereas no significant association was observed for all cause death. nsVT showed the highest PAR in cardiovascular death (13.02%, 95% CI 3.60–25.91%), and conversely, obstruction showed the highest PAR (10.09%, 95% CI 4.72–20.42%) for all-cause death and sudden cardiac death (16.44%, 95% CI 7.45–31.55%).

## Discussion

Hypertrophic cardiomyopathy is the most common monogenic heart disease characterized by unexplained LVH and myocardial fibrosis^18^. Physiologic consequences are highly variable and some patients with HCM are asymptomatic whereas others suffer severe heart failure and even sudden death. The prognosis is poor in a subset of affected individuals who rapidly progress to heart failure. Current clinical methods to assess the risk of these adverse events and to target therapy are somewhat limited. The main results of our analysis show that the pooled 1-, 3-, 5- and 10-year survival rates in the whole population were 98.0% (95% CI 97.4–98.6%), 94.3% (95% CI 93.1–95.6%), 82.2% (95% CI 75.2–89.2%) and 75.0% (95% CI 71.1–78.9%), respectively. Similar results have been reported previously in some independent cohort studies^21,35^. Given that heterogeneity was observed at 1, 3, 5 and 10 years, we undertook a meta-regression analysis to find possible reasons for heterogeneity. However, no correlation was found between survival heterogeneity and variables examined including time (mid–cohort year), publication date, sample size, geographical location and NOS scores. Furthermore, in our analysis, none of these variables included in the meta-regression was observed to substantially affect the predictive power of risk factors for HCM outcome.

In order to improve the homogeneity of the results, we performed subset analyses based on geographical location (America, Europe and Asia). The results for each group showed similar trends compared with the overall results but the statistic power was lower. Due to the limited data provided in original articles, detailed demographic characteristics could not be analyzed by meta-analysis. Therefore, further studies are needed to evaluate the influence of gender, ethnicity and race.

The currently accepted risk predictors for sudden cardiac death as an indication for primary prevention with implantable cardioverter defibrillators (ICDs) include family history of HCM-related sudden cardiac death, unexplained recent syncope, large left ventricular wall thickness (MLVWT ≥ 30 mm), multiple bursts of nsVT on ambulatory electrocardiography, and hypotensive or attenuated blood pressure response to exercise^7,18^. In our study, FHSD, recent syncope, MLVWT and obstruction (including left outflow obstruction and midventricular obstruction) showed significant correlation with all-cause death, cardiovascular death and sudden cardiac death, whereas abnormal blood pressure response to exercise was not significantly related to all-cause or cardiovascular death. As an indication of severe cardiac dysfunction, abnormally low systolic blood pressure (SBP) during exercise stress testing (LowExBP) is thought to be an ominous sign^36^. However, others have failed to identify significant differences in survival rates between patients with LowExBP and those with normal SBP responses^37^. Variation in clinical presentation, exercise mode (treadmill or bike), exercise intensity (moderate or maximal), and LowExBP category are potential explanations for these inconsistent results^38^. Due to the lack of required data in the original publications, we could not analyze the effect of abnormal blood pressure response accurately. It would be useful to establish standard procedures to guide the measurement of blood pressure response to exercise to facilitate the determination of the predictive value of this clinical variable.

FHSD is the most significant consideration for individual patients. The prognostic role of FHSD varied in previous studies (significant as an independent factor^39^ or significant in combination with syncope^40^ or insignificant^41,42^). In our study, FHSD showed strong predictive power for cardiovascular death and sudden cardiac death (HR = 2.38 and 2.34, respectively), but no significant correlation with all-cause death was observed. Moreover, FHSD showed a high PAR for cardiovascular death (11.31%) and sudden cardiac death (11.89%). The consideration that more FHSDs within a family confers greater risk of cardiac episodes had been suggested by Dimitrow *et al*.^7^, and genetic analysis is becoming a useful tool to identify factors which contribute to the risk of cardiac death, and to improve the early diagnosis of asymptomatic carriers among relatives^34^. Certain gene mutations may start to damage myocardium from the time of birth or even in utero, and increase the risk for cardiac death as the individual ages. It has been proposed to perform a survival analysis with follow-up from the moment of birth^7^. Studies included in our analyses were all performed in a traditional follow-up model (started at the first presentation of a patient at the institution), which may decrease the predictive power for cardiac death.

Large wall thickness (MWTh) ≥30 mm and non-sustained ventricular tachycardia in Holter monitoring of electrocardiography also appeared to be important prognostic factors in both cardiovascular death and sudden cardiac death, as suggested in previous studies^7,21^ and seen as well in our meta-analysis. Spirito and colleagues have suggested that severe left-ventricular hypertrophy (wall thickness ≥30 mm) alone is sufficient to warrant ICD therapy^7^, while another study suggested obstruction (including LVO and MVO) is a distinct phenotype of HCM associated with an unfavorable prognosis in terms of end-stage HCM, sudden death and lethal arrhythmic events^4^. Results of our analysis showed nsVT and obstruction were two of the most important risk predictors for cardiovascular death (HR = 2.46 and 1.52) and sudden cardiac death (HR = 2.92 and 2.41). nsVT also showed the highest PAR for cardiovascular death (PAR = 13.02%), partly because of a higher prevalence of nsVT (22.0%) compared to syncope (14.4%), which is significantly related with all-cause death (HR = 1.40), cardiovascular death (HR = 2.39) and sudden cardiac death (HR = 2.31).

Previous studies indicated male patients had a 3:2 predominance (59%)^35^. Our study showed a similar fraction (62.5%). Women with HCM were under-represented and more symptomatic than men, and showed higher risk of progression to advanced heart failure or death^29^. No significant correlation was observed between gender and survival in our study; more multicenter population studies need to be done to assess the impact of gender on the heterogeneous clinical profile and clinical course of HCM.

Advanced heart failure (NYHA III/IV) was reported as an independent predictor of cardiovascular mortality in end-stage HCM^16^, as patients with heart failure at diagnosis showed a trend toward more frequent adverse outcomes, probably because they had more advanced disease^11^. This was also confirmed in our study indicating that NYHA III/IV is an important prognostic factor for all-cause death (PAR = 7.79%) and cardiovascular death (PAR = 9.61%). In previous cohort studies, AF was reported to be a substantial risk factor for heart failure-related mortality and severe functional disability^12^, however, the significant relationship with AF disappeared in the multivariate model^16,27^. These results are in agreement with our study.

Left atrial diameter (LAD) and left ventricle ejection fraction (LVEF) are routine echocardiographic parameters used to assess cardiac function, but previous studies^32^ indicated the limitations of echocardiographic variables (including LAD and LVEF) in the assessment of HCM patients in clinical practice. In our study, no significant correlation was observed between these two variables and adverse clinical outcomes. EF is a poor measure of LV systolic performance when hypertrophy is present^43^ and the left atrium volume indexed to body surface area was reported^11^ because the LA enlargement is multifactorial, but the most common pathological mechanisms are systolic anterior motion (SAM)-related mitral regurgitation and elevated LV filling pressures.

### Limitations

Our study has several limitations. Some prognostic factors were assessed in only a few studies, thus weakening the effectiveness of meta-analysis. For instance, late gadolinium enhancement (LGE) by cardiac magnetic resonance imaging is a known predictor of adverse cardiovascular outcomes in patients with non-ischemic cardiomyopathy. However, we had to exclude this parameter due to insufficient data. Moreover, the number of studies was limited and we restricted our search strategy to articles published in English, so articles with potentially high-quality data that were published in other languages were not included because of anticipated difficulties in obtaining accurate medical translation. In this meta-analysis, we used inverse weighting proportional to variance. Therefore, larger studies, with presumably narrower confidence limits surrounding the point estimate of their effects, were given greater weight relative to smaller studies. Another possible limitation could arise if large studies included a narrower spectrum of disease and cofactors compared with small studies. In addition, different definitions of endpoint and length of follow-up could change the composition of the observed causes of mortality. The age of patients is primarily recorded at recruitment, hence other than HCM onset age, it is difficult to analyze the effect of age on prognosis^7^. Variable treatment strategies and availability of therapeutic agents were described in some included studies. The influence of these treatments was not assessed here because of the heterogeneity of the reported treatments and numerous missing data. The influence of treatment on survival has not yet been assessed in detail. Finally, no systematic genetic study was available in the majority of our patients; thus, it was not possible to assess the genotypic–phenotypic correlation and its prognostic implication, a main goal of future research.

## Conclusion

Our systematic review and meta-analysis aims to address important clinical questions regarding the pooled survival rates and prognostic factors influencing risk for death in patients with HCM. Evidence was observed for less favorable pooled survival rates with increasing durations of follow-up, however more studies with 10-year data are needed to increase confidence for the survival estimates, and larger prospective HCM outcome studies are needed. Some multicenter studies including the Hypertrophic Cardiomyopathy Registry are already underway and the HCM research community eagerly awaits their results (NCT01915615, NCT01091480, NCT01447654 and NCT02234336 *et al*.). Our findings indicate that known traditional HCM risk factors, such as FHSD and AF, have prognostic relevance, as expected. Judged by PAR, nsVT and LVO/MVO, respectively, are the most important risk factors for cardiovascular death and sudden cardiac death in HCM confirmed in our meta-analysis, and FHSD is also a highly important prognostic marker. Our results highlight the importance of early recognition of HCM and appropriate therapeutic interventions, including genetic testing and clinical evaluation in first-degree adult relatives, even if no definite genetic mutation is identified in the proband.

## Methods

This meta-analysis was performed according to the Preferred Reporting Items for Systematic Reviews and Meta-Analyses (PRISMA) Statement protocol^44^.

### Search strategy

We searched Medline, EMBase databases and the Cochrane Central Register of Controlled Trials database (January 1960 to September 2015) using the same strategy adapted to the specifics of each database. The search included the following terms: “hypertrophic cardiomyopathy” AND “death” OR “mortality” OR “clinical outcome” OR “survival” OR “prognosis”. In addition, the reference lists of the articles initially detected were searched by hand to identify additional relevant reports. The eligibility of references retrieved by the search was assessed independently by 2 of the authors (QL and DL), and disagreements were resolved at each step by input from LG and consensus. The full text of the remaining articles, including the references, was examined to determine whether the articles contained relevant information. Randomized clinical trials were excluded.

### Selection criteria

Studies were included if they met the following two criteria. First, the study group comprised patients with a diagnosis of HCM according to the European Society of Cardiology (ESC) 2014 Guidelines on diagnosis and management of hypertrophic cardiomyopathy^8^. HCM is defined by a wall thickness ≥15 mm in one or more LV myocardial segments—as measured by any imaging technique (echocardiography, cardiac magnetic resonance imaging or computed tomography)—that is not explained solely by loading conditions. Genetic and non-genetic disorders can present with lesser degrees of wall thickening (13–14 mm); in these cases, the diagnosis of HCM requires evaluation of other features including family history, non-cardiac symptoms and signs, electrocardiogram abnormalities, laboratory tests and multi-modality cardiac imaging. Second, the 1-, 3-, 5 years survival rates for patients with HCM were reported. Studies were excluded if the patients with HCM were ≤18 years old or had other causes of infiltrative/hypertrophic cardiomyopathies such as amyloidosis, sarcoidosis, Fabry disease, Danon disease, or Noonan syndrome (see Supplementary Methods). Quality assessment of individual studies was performed independently by 2 of the authors (DL and QL), using the Newcastle–Ottawa Scale for cohort studies. Disagreements between the reviewers were resolved by consensus with a 3rd author (LG) (see Supplementary Methods and Supplementary Table 1). The scale allocates stars (maximum of 9) for quality of selection, comparability, exposure, and outcome of study participants^45^. In addition, if we found more than one paper reporting on the same study population, the most recent one was included for further analysis. Any discrepancies were addressed by joint re-evaluation of the original article.

### Data extraction

The characteristics of the population (age, % male), follow-up duration, number and type of events, measured values from electrocardiogram and echocardiography, statistical analysis type and adjusted covariates from each eligible study were extracted for systematic review. For the meta-analysis, adjusted risk estimates and associated 95% CIs were extracted.

### Outcome assessment

The primary analysis focused on assessing pooled 1-, 3-, 5- and 10-year survival and prognostic factors for survival in HCM.

### Statistical analysis

We used the DerSimonian and Laird method^46^ to calculate pooled summary estimates of baseline characteristics (mean and proportion), mortality rate, and hazard ratio (HR) for mortality. Heterogeneity was quantified using a chi-square heterogeneity statistic and by means of an *I* ^2^ statistic for each meta-regression analysis^45^. A random-effects model was used to combine data. When the mortality rate and/or 95% confidence interval (95% CI) was not available, these values were estimated according to data obtained from the survival curve, using an actuarial method. The pooled mortality rate and HRs (provided in the adjusted value format) with 95% CIs were estimated and publication bias was estimated using the Eggers test with Begg’s funnel plots. For estimation of PAR, the standard formula was used^47^ (PAR = prevalence in exposed population x [(RR-1)/RR]. The 95% CI for PAR was calculated as ${\rm{P}}\hat{{\rm{A}}}{\rm{R}}$  ± 1.96 × $S\hat{{\rm{E}}}$ (${\rm{P}}\hat{{\rm{A}}}{\rm{R}}$)^43^. All analyses were performed using Stata 12.0 software and two-tailed*P* values less than 0.05 were considered significant. The datasets generated and analyzed during the current study are available from the corresponding author on reasonable request.

## Electronic supplementary material

Supplementary Information
