# Supplementary material for: Survival and prognostic factors in hypertrophic cardiomyopathy: a meta-analysis
Source: Sci Rep. 2017 Sep 20;7:11957. doi: 10.1038/s41598-017-12289-4 (PMC5607340; doi:10.1038/s41598-017-12289-4)
Supplement: Supplementary file 1 — Supplementary Information [file 41598_2017_12289_MOESM1_ESM.pdf]

Survival and prognostic factors in hypertrophic cardiomyopathy: a meta-analysis

Qun Liu[1,*], Diandian Li [2,*], Alan E. Berger[1], Roger A. Johns[3], Li Gao[1,#]

[1]Division of Allergy & Clinical Immunology, Johns Hopkins University School of Medicine, Baltimore, MD 21224, USA

[2]Division of Pulmonary Diseases, State Key Laboratory of Biotherapy of China and Department of Respiratory Medicine, West China Hospital of Sichuan University, Chengdu, Sichuan 610041, China

[3]Department of Anesthesiology and Critical Care Medicine, Johns Hopkins University School of Medicine, Baltimore, MD 21224, USA

[#]Correspondence to: Li Gao, Division of Allergy & Clinical Immunology, Johns Hopkins University School of Medicine, Baltimore, MD 21224, USA. E-mail: lgao2@jhmi.edu

Supplementary Methods

End point classification

The following events were used as end-points in the survival analysis:

(i) Sudden cardiac death: Witnessed sudden death with or without documented ventricular fibrillation, death within 1 h of new symptoms, or nocturnal death with no prior history of worsening symptoms[1].

(ii) Cardiovascular death: Preceded by new signs and/or symptoms of heart failure of more than 1 h duration and/or cardiogenic shock were considered as heart failure deaths. Deaths secondary to stroke, pulmonary or systemic embolism, or myocardial infarction were also considered as cardiovascular deaths.

(iii) All-cause death: All death reported during period of follow-up.

Exclude criteria

(i) Studies were excluded if the patients with HCM were ≤18 year or other causes of infiltrative/hypertrophic cardiomyopathies such as amyloidosis, sarcoidosis, Fabry disease, Danon disease, or Noonan syndrome.

(ii) randomized controlled trials and other clinic trails for interventional therapy or new medication

(iii) Reviews, conference abstracts or comments

Quality assessment of studies

Quality of included studies was assessed using the Newcastle–Ottawa scale. This scale consists of eight items assessing three aspects of a study: patient selection, comparability of study groups, and ascertainment of outcome. Each item could be awarded 1 point except for the item on comparability, which is awarded 2 points. Studies with scores of five to nine were considered high quality; those with scores of zero to four were considered low quality.

Results:

1. Selected articles during screening search (literature search)

Medline

1: Kammerlander AA, Marzluf BA, Zotter-Tufaro C, Aschauer S, Duca F, Bachmann A, Knechtelsdorfer K, Wiesinger M, Pfaffenberger S, Greiser A, Lang IM, Bonderman D, Mascherbauer J. T1 Mapping by CMR Imaging: From Histological Validation to Clinical Implication. JACC Cardiovasc Imaging. 2016 Jan;9(1):14-23. doi: 10.1016/j.jcmg.2015.11.002. Epub 2015 Dec 9. PubMed PMID: 26684970.

2: Magnusson P, Gadler F, Liv P, Mörner S. Risk Markers and Appropriate Implantable Defibrillator Therapy in Hypertrophic Cardiomyopathy. Pacing Clin Electrophysiol. 2016 Mar;39(3):291-301. doi: 10.1111/pace.12801. Epub 2016 Jan 21. PubMed PMID: 26681505.

3: Reich HJ, Awad M, Ruzza A, De Robertis MA, Ramzy D, Nissen N, Colquhoun S, Esmailian F, Trento A, Kobashigawa J, Czer LS. ombined Heart and Liver Transplantation: The Cedars-Sinai Experience. Transplant Proc. 2015 Nov;47(9):2722-6. doi: .1016/j.transproceed.2015.07.038. PubMed PMID: 26680081.

4: Peña-Peña ML, Gonzalvez-Garcia A, Navarro-Herrero S, Lucena-Romero J, Sanchez-Gomez A, Gonzalez-Campora R, Serrano-Gotarredona MP, Lopez-Pardo F, Lopez-Haldon JE, Urbano-Moral JA. Hypertrophic cardiomyopathy: The heart of the matter by looking into the matter of the heart. Int J Cardiol. 2016 Jan 15;203:573-4. doi: 10.1016/j.ijcard.2015.10.232. Epub 2015 Oct 30. PubMed PMID: 26569366.

5: Ziółkowska L, Turska-Kmieć A, Petryka J, Kawalec W. Predictors of Long-Term Outcome in Children with Hypertrophic Cardiomyopathy. Pediatr Cardiol. 2016 Mar;37(3):448-58. doi: 10.1007/s00246-015-1298-y. Epub 2015 Nov 2. PubMed PMID: 26526335; PubMed Central MCID: PMC4819755.

6: Ripoll-Vera T, Gámez JM, Govea N, Gómez Y, Núñez J, Socías L, Escandell Á, Rosell J. Clinical and Prognostic Profiles of ardiomyopathies Caused by Mutations in the Troponin T Gene. Rev Esp Cardiol (Engl Ed). 2016 Feb;69(2):149-58. doi: 10.1016/j.rec.2015.06.025. Epub 2015 Oct 24. PubMed PMID: 26507537.

7: Bertomeu-González V, Moreno-Arribas J, Castillo-Castillo J, Martínez-Ferrer J, Viñolas X, Rodríguez A, Díaz-Infante E, Fernández-Lozano I, Alzueta J, Fontenla A. Etiology and Programming Effects on Shock Efficacy in ICD Recipients. Pacing Clin Electrophysiol. 2016 Jan;39(1):73-80. doi: 10.1111/pace.12765. Epub 2015 Oct 30. PubMed PMID: 26450114.

8: Pasqualucci D, Fornaro A, Castelli G, Rossi A, Arretini A, Chiriatti C, Targetti M, Girolami F, Corda M, Orrù P, Matta G, Stefàno P, Cecchi F, Porcu M,Olivotto I. Clinical Spectrum, Therapeutic Options, and Outcome of Advanced HeartFailure in Hypertrophic Cardiomyopathy. Circ Heart Fail. 2015 Nov;8(6):1014-21.doi: 10.1161/CIRCHEARTFAILURE.114.001843. Epub 2015 Oct 7. PubMed PMID: 26446673.

9: Schaffer LR, Caltharp SA, Milla SS, Kogon BF, Cundiff CA, Dalal A, Quigley PC,Shehata BM. Rare presentation of four primary pediatric cardiac tumors.Cardiovasc Pathol. 2016 Jan-Feb;25(1):72-7. doi: 10.1016/j.carpath.2015.08.011.Epub 2015 Aug 31. PubMed PMID: 26419627.

10: Guo X, Fan C, Wang H, Zhao S, Duan F, Wang Z, Yan L, Yang Y, An S, Li Y. The Prevalence and Long-Term Outcomes of Extreme Right versus Extreme LeftVentricular Hypertrophic Cardiomyopathy. Cardiology. 2016;133(1):35-43. doi:10.1159/000439345. Epub 2015 Sep 29. PubMed PMID: 26414322.

11: Coats CJ, Rantell K, Bartnik A, Patel A, Mist B, McKenna WJ, Elliott PM.Cardiopulmonary Exercise Testing and Prognosis in Hypertrophic Cardiomyopathy.Circ Heart Fail. 2015 Nov;8(6):1022-31. doi: 10.1161/CIRCHEARTFAILURE.114.002248.Epub 2015 Sep 15. PubMed PMID: 26374874.

12: Ushikoshi H, Okada H, Morishita K, Imai H, Tomita H, Nawa T, Suzuki K,Ikeshoji H, Kato H, Yoshida T, Yoshida S, Shirai K, Toyoda I, Hara A, Ogura S. Anautopsy report of acute myocardial infarction with hypertrophic obstructivecardiomyopathy-like heart. Cardiovasc Pathol. 2015 Nov-Dec;24(6):405-7. doi:10.1016/j.carpath.2015.07.004. Epub 2015 Jul 11. PubMed PMID: 26251081.

13: Yang YJ, Yuan JQ, Fan CM, Pu JL, Fang PH, Ma J, Guo XY, Li YS. Incidence of ischemic stroke and systemic embolism in patients with hypertrophiccardiomyopathy, nonvalvular atrial fibrillation, CHA2DS2-VASc score of ≤1 andwithout anticoagulant therapy. Heart Vessels. 2016 Jul;31(7):1148-53. doi:10.1007/s00380-015-0718-5. Epub 2015 Aug 1. PubMed PMID: 26231425.

14: Troiano NH. Cardiomyopathy During Pregnancy. J Perinat Neonatal Nurs. 2015 Jul-Sep;29(3):222-8. doi: 10.1097/JPN.0000000000000113. PubMed PMID: 26218815.

15: Maron BJ, Casey SA, Chan RH, Garberich RF, Rowin EJ, Maron MS. Independent Assessment of the European Society of Cardiology udden Death Risk Model for Hypertrophic Cardiomyopathy. Am J Cardiol. 2015 Sep 1;116(5):757-64. doi: 10.1016/j.amjcard.2015.05.047. Epub 2015 Jun 4. PubMed PMID: 26183790.

16: Magnusson P, Gadler F, Liv P, Mörner S. Hypertrophic Cardiomyopathy and Implantable Defibrillators in Sweden: Inappropriate Shocks and Complications Requiring Surgery. J Cardiovasc Electrophysiol. 2015 Oct;26(10):1088-94. doi: 10.1111/jce.12750. Epub 2015 Sep 2. PubMed PMID: 26178879.

17: Jefferies JL, Wilkinson JD, Sleeper LA, Colan SD, Lu M, Pahl E, Kantor PF, Everitt MD, Webber SA, Kaufman BD, Lamour JM, Canter CE, Hsu DT, Addonizio LJ, Lipshultz SE, Towbin JA; Pediatric Cardiomyopathy Registry Investigators.. Cardiomyopathy Phenotypes and Outcomes

for Children With Left Ventricular Myocardial Noncompaction: Results From the Pediatric Cardiomyopathy Registry. J Card Fail. 2015 Nov;21(11):877-84. doi: 10.1016/j.cardfail.2015.06.381. Epub 2015 Jul 9. PubMed PMID: 26164213; PubMed Central PMCID: PMC4630116.

18: Wu W, Lu CX, Wang YN, Liu F, Chen W, Liu YT, Han YC, Cao J, Zhang SY, Zhang X. Novel Phenotype-Genotype Correlations of Restrictive Cardiomyopathy With Myosin-Binding Protein C (MYBPC3) Gene Mutations Tested by Next-Generation Sequencing. J Am Heart Assoc. 2015 Jul 10;4(7). pii: e001879. doi: 10.1161/JAHA.115.001879. PubMed PMID: 26163040; PubMed Central PMCID: PMC4608072.

19: Sharkey SW, Pink VR, Lesser JR, Garberich RF, Maron MS, Maron BJ. Clinical Profile of Patients With High-Risk Tako-Tsubo diomyopathy. Am J Cardiol. 2015 Sep 1;116(5):765-72. doi: 10.1016/j.amjcard.2015.05.054. Epub 2015 Jun 4. PubMed PMID: 26144453.

20: Zhu L, Wang J, Wang Y, Jia L, Sun K, Wang H, Zou Y, Tian T, Liu Y, Zou J, Hui R, Yuan Z, Song L. Plasma Uric Acid as a Prognostic Marker in Patients With Hypertrophic Cardiomyopathy. Can J Cardiol. 2015 Oct;31(10):1252-8. doi: 10.1016/j.cjca.2015.02.018. Epub 2015 Feb 20. PubMed PMID: 26111667.

21: Yang YJ, Fan CM, Yuan JQ, Qiao SB, Hu FH, Guo XY, Li YS. Survival after alcohol septal ablation versus conservative therapy in obstructive hypertrophic cardiomyopathy. Cardiol J. 2015;22(6):657-64. doi: 10.5603/CJ.a2015.0032. Epub 2015 Jun 23. PubMed PMID: 26100829.

22: Xiao Y, Yang KQ, Yang YK, Liu YX, Tian T, Song L, Jiang XJ, Zhou XL. Clinical Characteristics and Prognosis of End-stage Hypertrophic Cardiomyopathy. Chin Med J (Engl). 2015 Jun 5;128(11):1483-9. doi: 10.4103/0366-6999.157656. PubMed PMID: 26021505; PubMed Central PMCID: PMC4733774.

23: Wada Y, Aiba T, Matsuyama TA, Nakajima I, Ishibashi K, Miyamoto K, Yamada Y, Okamura H, Noda T, Satomi K, Morita Y, Kanzaki H, Kusano K, Anzai T, Kamakura S, Ishibashi-Ueda H, Shimizu W, Horie M, Yasuda S, Ogawa H. Clinical and Pathological Impact of Tissue Fibrosis on Lethal Arrhythmic Events in Hypertrophic Cardiomyopathy Patients With Impaired Systolic Function. Circ J. 2015;79(8):1733-41. doi: 10.1253/circj.CJ-15-0104. Epub 2015 May 27. PubMed PMID: 26016925.

24: Shin DG, Cho IJ, Shim CY, Ryu SK, Chang HJ, Hong GR, Ha JW, Chung N. Transient apical wall thickening in patients with stress cardiomyopathy: Prevalence, profile, and impact on clinical course. Int J Cardiol. 2015 Sep 1;194:87-92. doi: 10.1016/j.ijcard.2015.05.101. Epub 2015 May 19. PubMed PMID: 26011273.

25: Ayça B, Sahin I, Kucuk SH, Akin F, Kafadar D, Avşar M, Avci II, Gungor B,Okuyan E, Dinckal MH. Increased Transforming Growth Factor-β Levels AssociatedWith Cardiac Adverse Events in Hypertrophic Cardiomyopathy. Clin Cardiol. 2015Jun;38(6):371-7. doi: 0.1002/clc.22404. Epub 2015 May 14. PubMed PMID: 25973737.

26: Maron BJ, Rowin EJ, Casey SA, Link MS, Lesser JR, Chan RH, Garberich RF,Udelson JE, Maron MS. Hypertrophic Cardiomyopathy in Adulthood Associated WithLow Cardiovascular Mortality With Contemporary Management Strategies. J Am CollCardiol. 2015 May 12;65(18):1915-28. doi: 10.1016/j.jacc.2015.02.061. PubMed PMID: 25953744.

27: Gupta T, Harikrishnan P, Kolte D, Khera S, Aronow WS, Mujib M, Palaniswamy C, Sule S, Jain D, Ahmed A, Lanier GM, Cooper HA, Frishman WH, Fonarow GC, Panza JA. Outcomes of acute myocardial infarction in patients with hypertrophic cardiomyopathy. Am J Med. 2015 Aug;128(8):879-887.e1. doi: 10.1016/j.amjmed.2015.02.025. Epub 2015 Apr 22. PubMed PMID: 25910786.

28: Contreras-Valdes FM, Buxton AE, Josephson ME, Anter E. Atrial fibrillation ablation in patients with hypertrophic cardiomyopathy: long-term outcomes and clinical predictors. J Am Coll Cardiol. 2015 Apr 14;65(14):1485-7. doi: 10.1016/j.jacc.2014.12.063. PubMed PMID: 25857916.

29: Funada A, Kanzaki H, Noguchi T, Morita Y, Sugano Y, Ohara T, Hasegawa T, Hashimura H, Ishibashi-Ueda H, Kitakaze M, Yasuda S, Ogawa H, Anzai T. Prognostic significance of late gadolinium enhancement quantification in cardiac magnetic resonance imaging of hypertrophic cardiomyopathy with systolic dysfunction. Heart Vessels. 2016 May;31(5):758-70. doi: 10.1007/s00380-015-0670-4. Epub 2015 Mar 28. Erratum in: Heart Vessels. 2016 May;31(5):771-2. PubMed PMID: 25820658.

30: Sato T, Matsuyama TA, Seguchi O, Murata Y, Sunami H, Yanase M, Fujita T, Ishibashi-Ueda H, Nakatani T. Restrictive myocardium with an unusual pattern of apical hypertrophic cardiomyopathy. Cardiovasc Pathol. 2015 Jul-Aug;24(4):254-7. doi: 10.1016/j.carpath.2015.03.001. Epub 2015 Mar 6. PubMed PMID: 25804825.

31: Jacob D, Main ML, Gupta S, Gosch K, McCoy M, Magalski A. Prevalence and significance of isolated T wave inversion in 1755 consecutive American collegiate athletes. J Electrocardiol. 2015 May-Jun;48(3):407-14. doi: 10.1016/j.jelectrocard.2015.03.005. Epub 2015 Mar 6. PubMed PMID: 25795567.

32: Calore C, De Bortoli M, Romualdi C, Lorenzon A, Angelini A, Basso C, Thiene G, Iliceto S, Rampazzo A, Melacini P. A founder MYBPC3 mutation results in HCM with a high risk of sudden death after the fourth decade of life. J Med Genet. 2015 May;52(5):338-47. doi: 10.1136/jmedgenet-2014-102923. Epub 2015 Mar 4. PubMed PMID: 25740977.

33: Shin DG, Son JW, Park JY, Choi JW, Ryu SK. Impact of coronary artery anatomy on clinical course and prognosis in apical hypertrophic cardiomyopathy: analysis of coronary angiography and computed tomography. Korean Circ J. 2015Jan;45(1):38-43. doi: 0.4070/kcj.2015.45.1.38. Epub 2015 Jan 26. PubMed PMID:25653702; PubMed Central PMCID: PMC4310978.

34: Debonnaire P, Katsanos S, Joyce E, VAN DEN Brink OV, Atsma DE, Schalij MJ, Bax JJ, Delgado V, Marsan NA. QRS Fragmentation and QTc Duration Relate to Malignant Ventricular Tachyarrhythmias and Sudden Cardiac Death in Patients with Hypertrophic Cardiomyopathy. J Cardiovasc Electrophysiol. 2015 May;26(5):547-55. doi: 10.1111/jce.12629. Epub 2015 Mar 27. PubMed PMID: 25648421.

35: Zwadlo C, Schmidtmann E, Szaroszyk M, Kattih B, Froese N, Hinz H, Schmitto JD, Widder J, Batkai S, Bähre H, Kaever V, Thum T, Bauersachs J, Heineke J. Antiandrogenic therapy with finasteride attenuates cardiac hypertrophy and left ventricular dysfunction. Circulation. 2015 Mar 24;131(12):1071-81. doi: 10.1161/CIRCULATIONAHA.114.012066. Epub 2015 Jan 28. PubMed PMID: 25632043.

36: Vriesendorp PA, Schinkel AF, Soliman OI, Kofflard MJ, de Jong PL, van Herwerden LA, Ten Cate FJ, Michels M. Long-term benefit of myectomy and anterior mitral leaflet extension in obstructive hypertrophic cardiomyopathy. Am J Cardiol. 2015 Mar 1;115(5):670-5. doi: 10.1016/j.amjcard.2014.12.017. Epub 2014 Dec 18. PubMed PMID: 25591899.

37: Hartlage GR, Kim JH, Strickland PT, Cheng AC, Ghasemzadeh N, Pernetz MA, Clements SD, Williams BR 3rd. The prognostic value of standardized reference values for speckle-tracking global longitudinal strain in hypertrophic cardiomyopathy. Int J Cardiovasc Imaging. 2015 Mar;31(3):557-65. doi: 10.1007/s10554-015-0590-5. Epub 2015 Jan 14. PubMed PMID: 25585646.

38: Maron BJ, Nishimura RA. Surgical septal myectomy versus alcohol septalablation: assessing the status of the controversy in 2014. irculation. 2014 Oct 28;130(18):1617-24. doi: 10.1161/CIRCULATIONAHA.114.011580. PubMed PMID:25462821.

39: Sarrias A, Galve E, Sabaté X, Moya À, Anguera I, Núñez E, Villuendas R,Alcalde Ó, García-Dorado D. Implantable Cardioverter-efibrillator Therapy forHypertrophic Cardiomyopathy: Usefulness in Primary and Secondary Prevention. Rev Esp Cardiol (Engl Ed). 2015 Jun;68(6):492-6. doi: 10.1016/j.rec.2014.06.025. Epub2014 Nov 20. PubMed PMID: 25449813.

40: Vriesendorp PA, Liebregts M, Steggerda RC, Schinkel AF, Willems R, Ten CateFJ, van Cleemput J, Ten Berg JM, Michels M. Long-term outcomes after medical and invasive treatment in patients with hypertrophic cardiomyopathy. JACC Heart Fail.2014 Dec;2(6):630-6. doi: 10.1016/j.jchf.2014.06.012. Epub 2014 Oct 22. PubMedPMID: 25447346.

41: Talbot AS, Lewis NT, Nicholls KM. Cardiovascular outcomes in Fabry diseaseare linked to severity of chronic kidney disease. Heart. 2015 Feb;101(4):287-93. doi: 10.1136/heartjnl-2014-306278. Epub 2014 Nov 7. PubMed PMID: 25381325.

42: Steggerda RC, Damman K, Balt JC, Liebregts M, ten Berg JM, van den Berg MP.Periprocedural complications and long-term outcome after alcohol septal ablation versus surgical myectomy in hypertrophic obstructive cardiomyopathy: asingle-center experience. JACC Cardiovasc Interv. 2014 Nov;7(11):1227-34. doi:10.1016/j.jcin.2014.05.023. Epub 2014 Oct 15. PubMed PMID: 25326737.

43: Sedehi D, Finocchiaro G, Tibayan Y, Chi J, Pavlovic A, Kim YM, Tibayan FA,Reitz BA, Robbins RC, Woo J, Ha R, Lee DP, Ashley EA. Long-term outcomes ofseptal reduction for obstructive hypertrophic cardiomyopathy. J Cardiol. 2015Jul;66(1):57-62. doi:0.1016/j.jjcc.2014.08.010. Epub 2014 Sep 18. PubMed PMID: 25238885.

44: Panaich SS, Badheka AO, Chothani A, Mehta K, Patel NJ, Deshmukh A, Singh V,Savani GT, Arora S, Patel N, Bhalara V, Grover P, Shah N, Elder M, Mohamad T,Kaki A, Kondur A, Brown M, Grines C, Schreiber T. Results of ventricular septalmyectomy and hypertrophic ardiomyopathy (from Nationwide Inpatient Sample[1998-2010]). Am J Cardiol. 2014 Nov 1;114(9):1390-5. doi:10.1016/j.amjcard.2014.07.075. Epub 2014 Aug 12. PubMed PMID: 25205630.

45: Bombelli M, Facchetti R, Cuspidi C, Villa P, Dozio D, Brambilla G, Grassi G, Mancia G. Prognostic significance of left atrial enlargement in a generalpopulation: results of the PAMELA study. Hypertension. 2014 Dec;64(6):1205-11.doi: 10.1161/HYPERTENSIONAHA.114.03975. Epub 2014 Sep 8. PubMed PMID: 25201892.

46: Ishibashi-Ueda H, Ikeda Y, Matsuyama TA, Ohta-Ogo K, Sato T, Seguchi O,Yanase M, Fujita T, Kobayashi J, Nakatani T. The pathological implications ofheart transplantation: experience with 50 cases in a single center. Pathol Int.2014 Sep;64(9):423-31. doi: 10.1111/pin.12189. Epub 2014 Aug 22. PubMed PMID:25146228.

47: Wang J, Wang Y, Zou Y, Sun K, Wang Z, Ding H, Yuan J, Wei W, Hou Q, Wang H,Liu X, Zhang H, Ji Y, Zhou X, Sharma RK, Wang D, Ahmad F, Hui R, Song L.Malignant effects of multiple rare variants in sarcomere genes on the prognosis of patients with hypertrophic cardiomyopathy. Eur J Heart Fail. 2014Sep;16(9):950-7. doi: 10.1002/ejhf.144. Epub 2014 Jul 31. PubMed PMID: 25132132.

48: Inoue K, Murakawa Y, Nogami A, Shoda M, Naito S, Kumagai K, Miyauchi Y,Yamane T, Morita N, Okumura K; Japanese Heart Rhythm Society Members.. Clinicaland procedural predictors of early complications of ablation for atrialfibrillation: analysis of the national registry data. Heart Rhythm. 2014Dec;11(12):2247-53. doi: 10.1016/j.hrthm.2014.08.021. Epub 2014 Aug 15. PubMedPMID: 25131666.

49: Chan RH, Maron BJ, Olivotto I, Pencina MJ, Assenza GE, Haas T, Lesser JR,Gruner C, Crean AM, Rakowski H, Udelson JE, Rowin E, Lombardi M, Cecchi F,Tomberli B, Spirito P, Formisano F, Biagini E, Rapezzi C, De Cecco CN, Autore C, Cook EF, Hong SN, Gibson CM, Manning WJ, Appelbaum E, Maron MS. Prognostic value of quantitative contrast-enhanced cardiovascular magnetic resonance for the evaluation of sudden death risk in patients with hypertrophic cardiomyopathy.Circulation. 2014 Aug 5;130(6):484-95. oi: .1161/CIRCULATIONAHA.113.007094.PubMed PMID: 25092278.

50: Wang Y, Wang J, Zou Y, Bao J, Sun K, Zhu L, Tian T, Shen H, Zhou X, Ahmad F, Hui R, Song L. Female sex is associated with worse prognosis in patients withhypertrophic cardiomyopathy in China. PLoS One. 2014 Jul 21;9(7):e102969. doi:10.1371/journal.pone.0102969. eCollection 2014. PubMed PMID: 25047602; PubMedCentral PMCID: PMC4105411.

51: Yan LR, Zhao SH, Wang HY, Duan FJ, Wang ZM, Yang YJ, Guo XY, Cai C, Xu ZM, LiYS, Fan CM. Clinical characteristics and prognosis of 60 patients withmidventricular obstructive hypertrophic cardiomyopathy. J Cardiovasc Med(Hagerstown). 2015 Nov;16(11):751-60. doi: 10.2459/JCM.0000000000000163. PubMedPMID: 25022933.

52: Ismail TF, Jabbour A, Gulati A, Mallorie A, Raza S, Cowling TE, Das B, KhwajaJ, Alpendurada FD, Wage R, Roughton M, McKenna WJ, Moon JC, Varnava A,Shakespeare C, Cowie MR, Cook SA, Elliott P, O'Hanlon R, Pennell DJ, Prasad SK.Role of late gadolinium enhancement cardiovascular magnetic resonance in the riskstratification of hypertrophic cardiomyopathy. Heart. 2014 Dec;100(23):1851-8. doi: 10.1136/heartjnl-2013-305471. Epub 2014 Jun 24. PubMed PMID: 24966307.

53: Cho YH, Quintana E, Schaff HV, Nishimura RA, Dearani JA, Abel MD, Ommen S.Residual and recurrent gradients after septal myectomy for hypertrophiccardiomyopathy-mechanisms of obstruction and outcomes of reoperation. J ThoracCardiovasc Surg. 2014 Sep;148(3):909-15; discussion 915-6. doi:10.1016/j.jtcvs.2014.05.028. Epub 2014 May 16. PubMed PMID: 24930617.54: Caetano F, Botelho A, Trigo J, Silva J, Almeida I, Venâncio M, Pais J,Sanches C, Leitão Marques A. Phenotypic expression in hypertrophic cardiomyopathy and late gadolinium enhancement on cardiac magnetic resonance. Rev Port Cardiol. 2014 May;33(5):261-7. doi: 10.1016/j.repc.2013.10.005. Epub 2014 Jun 6. English, Portuguese. PubMed PMID: 24909443.

55: Borgeat K, Casamian-Sorrosal D, Helps C, Luis Fuentes V, Connolly DJ.Association of the myosin binding protein C3 mutation (MYBPC3 R820W) with cardiacdeath in a survey of 236 Ragdoll cats. J Vet Cardiol. 2014 Jun;16(2):73-80. doi: 10.1016/j.jvc.2014.03.005. Epub 2014 May 19. PubMed PMID: 24906243.

56: Kumare BD, Kawthalkar A. Pregnancy with hypertrophic cardiomyopathy--apotential risk factor for maternal mortality. Kathmandu Univ Med J (KUMJ). 2013Oct-Dec;11(44):335-7. PubMed PMID: 24899332.

57: Kang KW, Janardhan AH, Jung KT, Lee HS, Lee MH, Hwang HJ. Fragmented QRS as acandidate marker for high-risk assessment in hypertrophic cardiomyopathy. HeartRhythm. 2014 Aug;11(8):1433-40. doi: 10.1016/j.hrthm.2014.05.002. Epub 2014 May6. PubMed PMID: 24813377.

58: Efthimiadis GK, Pitsis A, Pagourelias ED, Kamperidis V, Kelpis T, MeditskouS, Hadjimiltiades S, Ninios V, Mezilis N, Maron BJ, Styliadis IH. Surgical septalmyectomy for hypertrophic cardiomyopathy in Greece: a single-center initialexperience. Hellenic J Cardiol. 2014 Mar-Apr;55(2):132-8. PubMed PMID: 24681791.

59: Fujita T, Fujino N, Anan R, Tei C, Kubo T, Doi Y, Kinugawa S, Tsutsui H,Kobayashi S, Yano M, Asakura M, Kitakaze M, Komuro I, Konno T, Hayashi K,Kawashiri MA, Ino H, Yamagishi M. Sarcomere gene mutations are associated withincreased cardiovascular events in left ventricular hypertrophy: results frommulticenter registration in Japan. JACC Heart Fail. 2013 Dec;1(6):459-66. doi:10.1016/j.jchf.2013.08.007. Epub 2013 Oct 24. PubMed PMID: 24621997.

60: Sakuma H. Late gadolinium enhancement and prognosis of hypertrophiccardiomyopathy. Circ J. 2014;78(4):832-4. Epub 2014 Mar 10. PubMed PMID:24614511.

61: Sathyamurthy I, Nayak R, Oomman A, Subramanyan K, Kalarical MS, Mao R,Ramachandran P. Alcohol septal ablation for hypertrophic obstructivecardiomyopathy - 8 years follow up. Indian Heart J. 2014 Jan-Feb;66(1):57-63.doi: 10.1016/j.ihj.2013.12.008. Epub 2013 Dec 25. PubMed PMID: 24581097; PubMedCentral PMCID: PMC5125590.

62: Sadat K, Diddi HP, Klas B, Asaad AH, Çekirdekçi Eİ, Sungur A, Sudhakar S,Cain M, Kamal A, Nanda NC. Live/real time three-dimensional transesophagealechocardiographic assessment of ventricular septal volume and mass before andafter myectomy in hypertrophic cardiomyopathy. Echocardiography. 2013Nov;30(10):1227-31. doi: 10.1111/echo.12375. PubMed PMID: 24579743.

63: Natali A, Boldrini B, Baldi S, Rossi M, Landi P, Severi S, Solini A,Ferrannini E. Impact of mild to moderate reductions of glomerular filtration rateon coronary artery disease severity. Nutr Metab Cardiovasc Dis. 2014Jun;24(6):681-8. doi: 10.1016/j.numecd.2013.12.005. Epub 2013 Dec 24. PubMedPMID: 24477005.

64: Cai C, Duan FJ, Yang YJ, Guo XY, Liu YL, Liu YQ, Yan LR, Xu ZM, Zhao SH, Hua W, Li YS, Fan CM. Comparison of the prevalence, clinical features, and long-term outcomes of midventricular hypertrophy vs apical phenotype in patients with hypertrophic cardiomyopathy. Can J Cardiol. 2014 Apr;30(4):441-7. doi:10.1016/j.cjca.2013.10.005. Epub 2013 Oct 9. PubMed PMID: 24468417.

65: Veselka J, Krejčí J, Tomašov P, Zemánek D. Long-term survival after alcoholseptal ablation for hypertrophic obstructive cardiomyopathy: a comparison withgeneral population. Eur Heart J. 2014 Aug 7;35(30):2040-5. doi:10.1093/eurheartj/eht495. Epub 2014 Jan 24. PubMed PMID: 24464834.

66: Nishi H, Toda K, Miyagawa S, Yoshikawa Y, Fukushima S, Saito S, Yoshioka D,Saito T, Ueno T, Sakaguchi T, Sawa Y. Initial experience in Japan with HeartWare ventricular assist system. J Artif Organs. 2014 Jun;17(2):149-56. doi:10.1007/s10047-013-0753-x. Epub 2014 Jan 25. PubMed PMID: 24464393.

67: Veselka J, Lawrenz T, Stellbrink C, Zemanek D, Branny M, Januska J, Sitar J, Dimitrow P, Krejci J, Dabrowski M, Mizera S, Bartel T, Kuhn H. Early outcomes of alcohol septal ablation for hypertrophic obstructive cardiomyopathy: a Europeanmulticenter and multinational study. Catheter Cardiovasc Interv. 2014 Jul1;84(1):101-7. doi: 10.1002/ccd.25236. Epub 2013 Nov 7. PubMed PMID: 24285605.

68: Luo MY, Wang SY, Sun HT, Yin ZH, Sun X, Song YH, Hu SS. [Clinical featuresand management of complete heart block after transaortic extended septal myectomyin patients with hypertrophic obstructive cardiomyopathy]. Zhonghua Xin Xue Guan Bing Za Zhi. 2013 Jul;41(7):598-601. Chinese. PubMed PMID: 24284190.

69: Katritsis DG, Siontis GC, Camm AJ. Prognostic significance of ambulatory ECG monitoring for ventricular arrhythmias. Prog Cardiovasc Dis. 2013Sep-Oct;56(2):133-42. doi: 10.1016/j.pcad.2013.07.005. Epub 2013 Sep 5. PubMedPMID: 24215745.

70: Tsaregorodtsev DA, Bukiia IR, Sulimov VA, Leont'eva IV, Makarova VA. [Heartrate turbulence and T-wave alternans as markers of risk of sudden cardiac deathin patients with hypertrophic cardiomyopathy]. Kardiologiia. 2013;53(9):40-6.Russian. PubMed PMID: 24090385.

71: Singh V, Badheka AO, Bokhari SS, Ghersin E, Clark PM, O'Neill WW. Retrograde percutaneous closure of a ventricular septal defect after myectomy forhypertrophic obstructive cardiomyopathy. Tex Heart Inst J. 2013;40(4):468-71.PubMed PMID: 24082382; PubMed Central PMCID: PMC3783118.

72: Kunkala MR, Schaff HV, Burkhart H, Sandhu GS, Spoon DB, Ommen SR, Sorajja P, Dearani JA. Outcome of repair of myocardial bridging at the time of septalmyectomy. Ann Thorac Surg. 2014 Jan;97(1):118-23. doi:10.1016/j.athoracsur.2013.07.079. Epub 2013 Sep 24. PubMed PMID: 24075496.

73: Vriesendorp PA, Schinkel AF, Van Cleemput J, Willems R, Jordaens LJ, TheunsDA, van Slegtenhorst MA, de Ravel TJ, ten Cate FJ, Michels M. Implantablecardioverter-defibrillators in hypertrophic cardiomyopathy: patient outcomes,rate of appropriate and inappropriate interventions, and complications. Am Heart J. 2013 Sep;166(3):496-502. doi: 10.1016/j.ahj.2013.06.009. Epub 2013 Jul 16.PubMed PMID: 24016499.

74: Lipshultz SE, Orav EJ, Wilkinson JD, Towbin JA, Messere JE, Lowe AM, Sleeper LA, Cox GF, Hsu DT, Canter CE, Hunter JA, Colan SD; Pediatric CardiomyopathyRegistry Study Group.. Risk stratification at diagnosis for children withhypertrophic cardiomyopathy: an analysis of data from the PediatricCardiomyopathy Registry. Lancet. 2013 Dec 7;382(9908):1889-97. doi:10.1016/S0140-6736(13)61685-2. Epub 2013 Sep 3. PubMed PMID: 24011547; PubMedCentral PMCID: PMC4007309.

75: Lyons KS, Dixon LJ, Johnston N, Noad R, Hamilton A, McKeag N, Horan P. Lategadolinium enhancement is common in patients with hypertrophic cardiomyopathy andno clinical risk factors for sudden cardiac death: A single center experience.Cardiol J. 2014;21(1):29-32. doi: 10.5603/CJ.a2013.0115. Epub 2013 Aug 30. PubMedPMID: 23990187.

76: Gutermann H, Pettinari M, Van Kerrebroeck C, Vander Laenen M, Engelen K, FretT, Dion RA. Myectomy and mitral repair through the left atrium in hypertrophicobstructive cardiomyopathy: the preferred approach for contemporary surgicalcandidates? J Thorac Cardiovasc Surg. 2014 Jun;147(6):1833-6. doi:10.1016/j.jtcvs.2013.07.024. Epub 2013 Aug 26. PubMed PMID: 23988290.

77: Habib A, Haque T. Pregnancy outcome in a case of non-obstructive hypertrophiccardiomyopathy. Mymensingh Med J. 2013 Jul;22(3):603-8. PubMed PMID: 23982559.

78: Anastasakis A, Theopistou A, Rigopoulos A, Kotsiopoulou C, Georgopoulos S,Fragakis K, Sevdalis E, Stefanadis C. Sudden cardiac death: investigation of the classical risk factors in a community-based hypertrophic cardiomyopathy cohort.Hellenic J Cardiol. 2013 Jul-Aug;54(4):281-8. PubMed PMID: 23912920.

79: El-Saiedi SA, Seliem ZS, Esmail RI. Hypertrophic cardiomyopathy: prognosticfactors and survival analysis in 128 Egyptian patients. Cardiol Young. 2014Aug;24(4):702-8. doi: 10.1017/S1047951113001030. Epub 2013 Jul 29. PubMed PMID:23895893.

80: Samardhi H, Walters DL, Raffel C, Rateesh S, Harley C, Burstow D, Pohlner P, Aroney C. The long-term outcomes of transcoronary ablation of septal hypertrophy compared to surgical myectomy in patients with symptomatic hypertrophicobstructive cardiomyopathy. Catheter Cardiovasc Interv. 2014 Feb;83(2):270-7.doi: 10.1002/ccd.25134. Epub 2013 Aug 31. PubMed PMID: 23873660.

81: D'Amato R, Tomberli B, Castelli G, Spoladore R, Girolami F, Fornaro A,Caldini A, Torricelli F, Camici P, Gensini GF, Cecchi F, Olivotto I. Prognosticvalue of N-terminal pro-brain natriuretic Peptide in outpatients withhypertrophic cardiomyopathy. Am J Cardiol. 2013 Oct 15;112(8):1190-6. doi:10.1016/j.amjcard.2013.06.018. Epub 2013 Jul 19. PubMed PMID: 23871673.

82: Lucon A, Palud L, Pavin D, Donal E, Behar N, Leclercq C, Mabo P, Daubert JC. Very late effects of dual chamber pacing therapy for obstructive hypertrophiccardiomyopathy. Arch Cardiovasc Dis. 2013 Jun-Jul;106(6-7):373-81. doi:10.1016/j.acvd.2013.04.003. Epub 2013 Jun 24. PubMed PMID: 23806305.

83: Takigawa M, Kuwahara T, Takahashi A, Watari Y, Okubo K, Takahashi Y, TakagiK, Kuroda S, Osaka Y, Kawaguchi N, Yamao K, Nakashima E, Sugiyama T, Akiyama D,Kamiishi T, Kimura S, Hikita H, Hirao K, Isobe M. Differences in catheter ablation of paroxysmal atrial fibrillation between males and females. Int JCardiol. 2013 Oct 3;168(3):1984-91. doi: 10.1016/j.ijcard.2012.12.101. Epub 2013 Feb 4. PubMed PMID: 23782910.

84: Desai MY, Bhonsale A, Smedira NG, Naji P, Thamilarasan M, Lytle BW, Lever HM.Predictors of long-term outcomes in symptomatic hypertrophic obstructivecardiomyopathy patients undergoing surgical relief of left ventricular outflowtract obstruction. Circulation. 2013 Jul 16;128(3):209-16. doi:10.1161/CIRCULATIONAHA.112.000849. Epub 2013 Jun 14. PubMed PMID: 23770748.

85: Efthimiadis GK, Pagourelias ED, Parcharidou D, Gossios T, Kamperidis V,Theofilogiannakos EK, Pappa Z, Meditskou S, Hadjimiltiades S, Pliakos C,Karvounis H, Styliadis IH. Clinical characteristics and natural history ofhypertrophic cardiomyopathy with midventricular obstruction. Circ J.2013;77(9):2366-74. Epub 2013 May 31. PubMed PMID: 23728066.

86: Sherrid MV, Shetty A, Winson G, Kim B, Musat D, Alviar CL, Homel P, BalaramSK, Swistel DG. Treatment of obstructive hypertrophic cardiomyopathy symptoms andgradient resistant to first-line therapy with β-blockade or verapamil. Circ HeartFail. 2013 Jul;6(4):694-702. doi: 10.1161/CIRCHEARTFAILURE.112.000122. Epub 2013 May 23. PubMed PMID: 23704138.

87: Luo MY, Wang SY, Song YH, Yin CH, Sun HT, Sun X, Xu N, Xu JP, Wang W, Hu SS. [Surgical treatment for hypertrophic obstructive cardiomyopathy: a report of 118 cases]. Zhonghua Yi Xue Za Zhi. 2013 Jan 8;93(2):110-3. Chinese. PubMed PMID:23648346.

88: Jensen MK, Prinz C, Horstkotte D, van Buuren F, Bitter T, Faber L, Bundgaard H. Alcohol septal ablation in patients with hypertrophic obstructivecardiomyopathy: low incidence of sudden cardiac death and reduced risk profile.Heart. 2013 Jul;99(14):1012-7. doi: 10.1136/heartjnl-2012-303339. Epub 2013 May3. PubMed PMID: 23644300.

89: Kubo T, Kitaoka H, Yamanaka S, Hirota T, Baba Y, Hayashi K, Iiyama T, KumagaiN, Tanioka K, Yamasaki N, Matsumura Y, Furuno T, Sugiura T, Doi YL. Significance of high-sensitivity cardiac troponin T in hypertrophic cardiomyopathy. J Am Coll Cardiol. 2013 Oct 1;62(14):1252-9. doi: 10.1016/j.jacc.2013.03.055. Epub 2013 Apr23. PubMed PMID: 23623916.

90: Hayashi T, Fukamizu S, Hojo R, Komiyama K, Tanabe Y, Tejima T, Soejima K,Nishizaki M, Hiraoka M, Ako J, Momomura S, Sakurada H. Prophylactic catheterablation for induced monomorphic ventricular tachycardia in patients withimplantable cardioverter defibrillators as primary prevention. Europace. 2013Oct;15(10):1507-15. doi: 10.1093/europace/eut050. Epub 2013 Apr 19. PubMed PMID: 23603305.

91: Geske JB, McKie PM, Ommen SR, Sorajja P. B-type natriuretic peptide andsurvival in hypertrophic cardiomyopathy. J Am Coll Cardiol. 2013 Jun18;61(24):2456-60. doi: 10.1016/j.jacc.2013.04.004. Epub 2013 Apr 16. PubMedPMID: 23602778.

92: Moravsky G, Ofek E, Rakowski H, Butany J, Williams L, Ralph-Edwards A,Wintersperger BJ, Crean A. Myocardial fibrosis in hypertrophic cardiomyopathy:accurate reflection of histopathological findings by CMR. JACC CardiovascImaging. 2013 May;6(5):587-96. doi: 10.1016/j.jcmg.2012.09.018. Epub 2013 Apr 10.PubMed PMID: 23582356.

93: Klarich KW, Attenhofer Jost CH, Binder J, Connolly HM, Scott CG, Freeman WK, Ackerman MJ, Nishimura RA, Tajik AJ, Ommen SR. Risk of death in long-termfollow-up of patients with apical hypertrophic cardiomyopathy. Am J Cardiol. 2013Jun 15;111(12):1784-91. doi: 10.1016/j.amjcard.2013.02.040. Epub 2013 Mar 27.Erratum in: Am J Cardiol. 2013 Oct 15;112(8):1271. PubMed PMID: 23540548.

94: Kamp AN, Von Bergen NH, Henrikson CA, Makhoul M, Saarel EV, Lapage MJ,Russell MW, Strieper M, Yu S, Dick M, Day SM, Bradley DJ. Implanteddefibrillators in young hypertrophic cardiomyopathy patients: a multicenterstudy. Pediatr Cardiol. 2013 Oct;34(7):1620-7. doi: 10.1007/s00246-013-0676-6.Epub 2013 Mar 20. PubMed PMID: 23512332.

95: Maron BJ, Spirito P, Ackerman MJ, Casey SA, Semsarian C, Estes NA 3rd,Shannon KM, Ashley EA, Day SM, Pacileo G, Formisano F, Devoto E, Anastasakis A,Bos JM, Woo A, Autore C, Pass RH, Boriani G, Garberich RF, Almquist AK, RussellMW, Boni L, Berger S, Maron MS, Link MS. Prevention of sudden cardiac death with implantable cardioverter-defibrillators in children and adolescents with hypertrophic cardiomyopathy. J Am Coll Cardiol. 2013 Apr 9;61(14):1527-35. doi:10.1016/j.jacc.2013.01.037. PubMed PMID: 23500286.

96: Fujita E, Nakanishi T, Nishizawa T, Hagiwara N, Matsuoka R. Mutations in the cardiac troponin T gene show various prognoses in Japanese patients withhypertrophic cardiomyopathy. Heart Vessels. 2013 Nov;28(6):785-94. doi:10.1007/s00380-013-0332-3. Epub 2013 Mar 14. PubMed PMID: 23494605; PubMedCentral PMCID: PMC3830204.

97: Coats CJ, Gallagher MJ, Foley M, O'Mahony C, Critoph C, Gimeno J, Dawnay A,McKenna WJ, Elliott PM. Relation between serum N-terminal pro-brain natriureticpeptide and prognosis in patients with hypertrophic cardiomyopathy. Eur Heart J. 2013 Aug;34(32):2529-37. doi: 10.1093/eurheartj/eht070. Epub 2013 Mar 1. PubMedPMID: 23455360.

98: Heredia Cambra T, Doñate Bertolín L, Bel Mínguez AM, Hernández Acuña CE,Schuler M, Pérez Guillén M, Margarit Calabuig JA, Montero Argudo JA. Experience, outcomes and impact of delayed indication for video-assisted wide septal myectomyin 69 consecutive patients with hypertrophic cardiomyopathy. Eur J CardiothoracSurg. 2013 Aug;44(2):e149-55; discussion e155. doi: 10.1093/ejcts/ezt050. Epub2013 Feb 21. PubMed PMID: 23428579.

99: O'Mahony C, Tome-Esteban M, Lambiase PD, Pantazis A, Dickie S, McKenna WJ,Elliott PM. A validation study of the 2003 American College ofCardiology/European Society of Cardiology and 2011 American College of CardiologyFoundation/American Heart Association risk stratification and treatmentalgorithms for sudden cardiac death in patients with hypertrophic cardiomyopathy.Heart. 2013 Apr;99(8):534-41. doi: 10.1136/heartjnl-2012-303271. Epub 2013 Jan22. PubMed PMID: 23339826.

100: Griksaitis MJ, Rosengarten JA, Gnanapragasam JP, Haw MP, Morgan JM.Implantable cardioverter defibrillator therapy in paediatric practice: asingle-centre UK experience with focus on subcutaneous defibrillation. Europace. 2013 Apr;15(4):523-30. doi: 10.1093/europace/eus388. Epub 2013 Jan 20. PubMedPMID: 23333943.

101: Maron BJ, Rowin EJ, Casey SA, Haas TS, Chan RH, Udelson JE, Garberich RF,Lesser JR, Appelbaum E, Manning WJ, Maron MS. Risk stratification and outcome of patients with hypertrophic cardiomyopathy >=60 years of age. Circulation. 2013Feb 5;127(5):585-93. doi: 10.1161/CIRCULATIONAHA.112.136085. Epub 2012 Dec 30.PubMed PMID: 23275385.

102: Olde Nordkamp LR, Wilde AA, Tijssen JG, Knops RE, van Dessel PF, de GrootJR. The ICD for primary prevention in patients with inherited cardiac diseases:indications, use, and outcome: a comparison with secondary prevention. CircArrhythm Electrophysiol. 2013 Feb;6(1):91-100. doi: 10.1161/CIRCEP.112.975268.Epub 2012 Dec 29. PubMed PMID: 23275262.

103: Orme NM, Sorajja P, Dearani JA, Schaff HV, Gersh BJ, Ommen SR. Comparison ofsurgical septal myectomy to medical therapy alone in patients with hypertrophiccardiomyopathy and syncope. Am J Cardiol. 2013 Feb 1;111(3):388-92. doi:10.1016/j.amjcard.2012.10.014. Epub 2012 Nov 17. PubMed PMID: 23168291.

104: Bates MG, Hollingsworth KG, Newman JH, Jakovljevic DG, Blamire AM, MacGowan GA, Keavney BD, Chinnery PF, Turnbull DM, Taylor RW, Trenell MI, Gorman GS.Concentric hypertrophic remodelling and subendocardial dysfunction inmitochondrial DNA point mutation carriers. Eur Heart J Cardiovasc Imaging. 2013Jul;14(7):650-8. doi: 10.1093/ehjci/jes226. Epub 2012 Nov 4. PubMed PMID:23129433; PubMed Central PMCID: PMC3681541.

105: Terrier B, Karras A, Cluzel P, Collet JP, Sène D, Saadoun D, Cacoub P.Presentation and prognosis of cardiac involvement in hepatitis C virus-relatedvasculitis. Am J Cardiol. 2013 Jan 15;111(2):265-72. doi:10.1016/j.amjcard.2012.09.028. Epub 2012 Oct 30. PubMed PMID: 3116612.

106: Maron BJ, Haas TS, Ahluwalia A, Garberich RF, Estes NA 3rd, Link MS.Increasing survival rate from commotio cordis. Heart Rhythm. 2013Feb;10(2):219-23. doi: 10.1016/j.hrthm.2012.10.034. Epub 2012 Oct 26. PubMedMID: 23107651.

107: Gao C, Ren C, Xiao C, Wu Y, Wang G, Liu G, Wang Y. Extended myectomy forhypertrophic obstructive cardiomyopathy. Heart Surg Forum. 2012 Oct;15(5):E251-6.doi: 10.1532/HSF98.20111185. PubMed PMID: 23092660.

108: Goto D, Kinugawa S, Hamaguchi S, Sakakibara M, Tsuchihashi-Makaya M, Yokota T, Yamada S, Yokoshiki H, Tsutsui H; JCARE-CARD Investigators.. Clinicalcharacteristics and outcomes of dilated phase of hypertrophic cardiomyopathy:report from the registry data in Japan. J Cardiol. 2013 Jan;61(1):65-70. doi:10.1016/j.jjcc.2012.08.010. Epub 2012 Oct 15. PubMed PMID: 23078864.

109: Sorajja P, Ommen SR, Holmes DR Jr, Dearani JA, Rihal CS, Gersh BJ, LennonRJ, Nishimura RA. Survival after alcohol septal ablation for obstructivehypertrophic cardiomyopathy. Circulation. 2012 Nov 13;126(20):2374-80. doi:10.1161/CIRCULATIONAHA.111.076257. Epub 2012 Oct 17. PubMed PMID: 23076968.

110: Kitaoka H, Kubo T, Hayashi K, Yamasaki N, Matsumura Y, Furuno T, Doi YL.Tissue Doppler imaging and prognosis in asymptomatic or mildly symptomaticpatients with hypertrophic cardiomyopathy. Eur Heart J Cardiovasc Imaging. 2013Jun;14(6):544-9. doi: 10.1093/ehjci/jes200. Epub 2012 Oct 11. PubMed PMID:23060455.

111: Alsheikh-Ali AA, Link MS, Semsarian C, Shen WK, Estes NA 3rd, Maron MS, HaasTS, Formisano F, Boriani G, Spirito P, Maron BJ. Ventriculartachycardia/fibrillation early after defibrillator implantation in patients with hypertrophic cardiomyopathy is explained by a high-risk subgroup of patients.Heart Rhythm. 2013 Feb;10(2):214-8. doi: 10.1016/j.hrthm.2012.10.003. Epub 2012Oct 4. PubMed PMID: 23041573.

112: Leonardi RA, Townsend JC, Patel CA, Wolf BJ, Todoran TM, Powers ER,Steinberg DH, Fernandes VL, Nielsen CD. Alcohol septal ablation for obstructivehypertrophic cardiomyopathy: outcomes in young, middle-aged, and elderlypatients. Catheter Cardiovasc Interv. 2013 Nov 1;82(5):838-45. doi:10.1002/ccd.24643. Epub 2013 Mar 25. PubMed PMID: 22936613.

113: Singh TP, Almond CS, Piercey G, Gauvreau K. Current outcomes in US children with cardiomyopathy listed for heart transplantation. Circ Heart Fail. 2012 Sep1;5(5):594-601. Epub 2012 Aug 16. PubMed PMID: 22899768.

114: Finocchiaro G, Pinamonti B, Merlo M, Brun F, Barbati G, Sinagra G.Prognostic role of clnical presentation in symptomatic patients with

hypertrophic cardiomyopathy. J Cardiovasc Med (Hagerstown). 2012Dec;13(12):810-8. doi: 10.2459/JCM.0b013e328356a231. PubMed PMID: 22885530.

115: Wong TC, Piehler K, Meier CG, Testa SM, Klock AM, Aneizi AA, Shakesprere J, Kellman P, Shroff SG, Schwartzman DS, Mulukutla SR, Simon MA, Schelbert EB.Association between extracellular matrix expansion quantified by cardiovascularmagnetic resonance and short-term mortality. Circulation. 2012 Sep4;126(10):1206-16. doi: 10.1161/CIRCULATIONAHA.111.089409. Epub 2012 Jul 31.

PubMed PMID: 22851543; PubMed Central PMCID: PMC3464491.

116: Webber SA, Lipshultz SE, Sleeper LA, Lu M, Wilkinson JD, Addonizio LJ,Canter CE, Colan SD, Everitt MD, Jefferies JL, Kantor PF, Lamour JM, MargossianR, Pahl E, Rusconi PG, Towbin JA; Pediatric Cardiomyopathy RegistryInvestigators.. Outcomes of restrictive cardiomyopathy in childhood and theinfluence of phenotype: a report from the Pediatric Cardiomyopathy Registry.Circulation. 2012 Sep 4;126(10):1237-44. Epub 2012 Jul 27. PubMed PMID: 22843787.

117: Nappi G, Dialetto G, De Feo M, Provenzano R, Cotrufo M, Santè P, Della CorteA. Apico-aortic conduit for severe hypertrophic cardiomyopathy: sudden deathdespite conduit patency 31 years postoperatively. Ann Thorac Cardiovasc Surg.2013;19(1):63-6. Epub 2012 Jun 29. PubMed PMID: 22785550.

118: Greulich S, Schumm J, Grün S, Bruder O, Sechtem U, Mahrholdt H. Incremental value of late gadolinium enhancement for management of patients with hypertrophiccardiomyopathy. Am J Cardiol. 2012 Oct 15;110(8):1207-12. doi:10.1016/j.amjcard.2012.05.064. Epub 2012 Jul 3. PubMed PMID: 22762717.

119: Gordon SG, Saunders AB, Roland RM, Winter RL, Drourr L, Achen SE, Hariu CD, Fries RC, Boggess MM, Miller MW. Effect of oral administration of pimobendan incats with heart failure. J Am Vet Med Assoc. 2012 Jul 1;241(1):89-94. doi:10.2460/javma.241.1.89. PubMed PMID: 22720992.

120: Kato TS, Takayama H, Yoshizawa S, Marboe C, Schulze PC, Farr M, Naka Y,Mancini D, Maurer MS. Cardiac transplantation in patients with hypertrophiccardiomyopathy. Am J Cardiol. 2012 Aug 15;110(4):568-74. doi:10.1016/j.amjcard.2012.04.030. Epub 2012 May 15. PubMed PMID: 22591671.

121: Iacovoni A, Spirito P, Simon C, Iascone M, Di Dedda G, De Filippo P,Pentiricci S, Boni L, Senni M, Gavazzi A, Ferrazzi P. A contemporary Europeanexperience with surgical septal myectomy in hypertrophic cardiomyopathy. EurHeart J. 2012 Aug;33(16):2080-7. doi: 10.1093/eurheartj/ehs064. Epub 2012 Apr 20.PubMed PMID: 22522842; PubMed Central PMCID: PMC3418509.

122: Sorajja P, Binder J, Nishimura RA, Holmes DR Jr, Rihal CS, Gersh BJ,Bresnahan JF, Ommen SR. Predictors of an optimal clinical outcome with alcoholseptal ablation for obstructive hypertrophic cardiomyopathy. Catheter Cardiovasc Interv. 2013 Jan 1;81(1):E58-67. doi: 10.1002/ccd.24328. Epub 2012 Apr 17. PubMedPMID: 22511295.

123: Birks EJ, George RS, Firouzi A, Wright G, Bahrami T, Yacoub MH, Khaghani A. Long-term outcomes of patients bridged to recovery versus patients bridged totransplantation. J Thorac Cardiovasc Surg. 2012 Jul;144(1):190-6. doi:10.1016/j.jtcvs.2012.03.021. Epub 2012 Apr 11. PubMed PMID: 22498081.

124: Maron BJ, Casey SA, Haas TS, Kitner CL, Garberich RF, Lesser JR.Hypertrophic cardiomyopathy with longevity to 90 years or older. Am J Cardiol.2012 May 1;109(9):1341-7. doi: 10.1016/j.amjcard.2011.12.027. Epub 2012 Feb 28.PubMed PMID: 381158.

125: Di Tommaso L, Stassano P, Mannacio V, Russolillo V, Monaco M, Pinna G, Vosa C. Asymmetric septal hypertrophy in patients with severe aortic stenosis: theusefulness of associated septal myectomy. J Thorac Cardiovasc Surg. 2013Jan;145(1):171-5. doi: 10.1016/j.jtcvs.2011.10.096. Epub 2012 Feb 15. PubMedPMID: 22341422.

126: Page SP, Kounas S, Syrris P, Christiansen M, Frank-Hansen R, Andersen PS,Elliott PM, McKenna WJ. Cardiac myosin binding protein-C mutations in familieswith hypertrophic cardiomyopathy: disease expression in relation to age, gender, and long term outcome. Circ Cardiovasc Genet. 2012 Apr 1;5(2):156-66. doi:10.1161/CIRCGENETICS.111.960831. Epub 2012 Jan 20. PubMed PMID: 22267749.

127: Kaimoto S, Kawasaki T, Kuribayashi T, Yamano M, Miki S, Kamitani T,Matsubara H. Myocardial perfusion abnormality in the area of ventricularseptum-free wall junction and cardiovascular events in nonobstructivehypertrophic cardiomyopathy. Int J Cardiovasc Imaging. 2012 Oct;28(7):1829-39.doi: 10.1007/s10554-011-9994-z. Epub 2011 Dec 21. PubMed PMID: 22187199.

128: Vassileva CM, Boley T, Markwell S, Hazelrigg S. Mitral valve repair isunderused in patients with hypertrophic obstructive cardiomyopathy. Heart SurgForum. 2011 Dec;14(6):E376-9. doi: 10.1532/HSF98.20111067. PubMed PMID: 22167765.

129: Schaff HV, Dearani JA, Ommen SR, Sorajja P, Nishimura RA. Expanding theindications for septal myectomy in patients with hypertrophic cardiomyopathy:results of operation in patients with latent obstruction. J Thorac CardiovascSurg. 2012 Feb;143(2):303-9. doi: 10.1016/j.jtcvs.2011.10.059. Epub 2011 Dec 10. PubMed PMID: 22154797.

130: Anagnostopoulos PV, Johnson NC, Robertson L, Sapru A, Azakie A. Surgicalmanagement of left ventricular outflow tract obstruction. J Card Surg. 2012Jan;27(1):103-11. doi: 10.1111/j.1540-8191.2011.01359.x. Epub 2011 Dec 12. PubMedPMID: 22150843.

131: Yan L, Wang Z, Xu Z, Li Y, Tao Y, Fan C. Two hundred eight patients withapical hypertrophic cardiomyopathy in china: clinical feature, prognosis, andcomparison of pure and mixed forms. Clin Cardiol. 2012 Feb;35(2):101-6. doi:10.1002/clc.20995. Epub 2011 Nov 28. PubMed PMID: 22125122.

132: Nannenberg EA, Michels M, Christiaans I, Majoor-Krakauer D, Hoedemaekers YM,van Tintelen JP, Lombardi MP, ten Cate FJ, Schinkel AF, Tijssen JG, van LangenIM, Wilde AA, Sijbrands EJ. Mortality risk of untreated myosin-binding proteinC-related hypertrophic cardiomyopathy: insight into the natural history. J AmColl Cardiol. 2011 Nov 29;58(23):2406-14. doi: 10.1016/j.jacc.2011.07.044.

Erratum in: J Am Coll Cardiol. 2012 Feb 14;59(7):702. PubMed PMID: 22115648.

133: Cardim N, Freitas A, Brito D. From hypertrophic cardiomyopathy centers toinherited cardiovascular disease centers in Europe. A small or a major step? Aposition paper from the Nucleus of the Working Group on Myocardial andPericardial Diseases of the Portuguese Society of Cardiology. Rev Port Cardiol.2011 Nov;30(11):829-35. doi: 10.1016/j.repc.2011.09.005. PubMed PMID: 22030325.

134: Moon J, Shim CY, Ha JW, Cho IJ, Kang MK, Yang WI, Jang Y, Chung N, Cho SY.Clinical and echocardiographic predictors of outcomes in patients with apicalhypertrophic cardiomyopathy. Am J Cardiol. 2011 Dec 1;108(11):1614-9. doi:10.1016/j.amjcard.2011.07.024. Epub 2011 Sep 3. PubMed PMID: 21890076.

135: Lisboa LA, Dallan LA, Pomerantzeff PM, Oliveiras SA, Jatene FB, Stolf NA.Long term results of septal myectomy in the treatment of obstructive hypertrophiccardiomyopathy. Rev Bras Cir Cardiovasc. 2011 Jan-Mar;26(1):86-92. English,Portuguese. PubMed PMID: 21881716.

136: Pagourelias ED, Efthimiadis GK, Parcharidou DG, Gossios TD, Kamperidis V,Karoulas T, Karvounis H, Styliadis IH. Prognostic value of right ventriculardiastolic function indices in hypertrophic cardiomyopathy. Eur J Echocardiogr.2011 Nov;12(11):809-17. doi: 10.1093/ejechocard/jer126. Epub 2011 Aug 15. PubMed PMID: 21846651.

137: Koźluk E, Gaj S, Kiliszek M, Lodziński P, Piątkowska A, Opolski G. Efficacy of catheter ablation in patients with an electrical storm. Kardiol Pol.2011;69(7):665-70. PubMed PMID: 21769783.

138: O'Mahony C, Lambiase PD, Quarta G, Cardona M, Calcagnino M, Tsovolas K,Al-Shaikh S, Rahman SM, Arnous S, Jones S, McKenna W, Elliott P. The long-termsurvival and the risks and benefits of implantable cardioverter defibrillators inpatients with hypertrophic cardiomyopathy. Heart. 2012 Jan;98(2):116-25. doi:10.1136/hrt.2010.217182. Epub 2011 Jul 13. PubMed PMID: 21757459.

139: Maron BJ, Maron MS, Maron BA, Haas TS, Altman RK, Smalley SJ, Doerer JJ,Link MS, Mooney MR. Successful therapeutic hypothermia in patients withhypertrophic cardiomyopathy. J Am Coll Cardiol. 2011 Jun 14;57(24):2454-6. doi:10.1016/j.jacc.2011.03.021. PubMed PMID: 21640539.

140: Fernández A, Vigliano CA, Casabé JH, Diez M, Favaloro LE, Guevara E,Favaloro RR, Laguens RP. Comparison of prevalence, clinical course, andpathological findings of left ventricular systolic impairment versus normalsystolic function in patients with hypertrophic cardiomyopathy. Am J Cardiol.2011 Aug 15;108(4):548-55. doi: 10.1016/j.amjcard.2011.03.083. Epub 2011 May 31. PubMed PMID: 21624540.

141: Jensen MK, Havndrup O, Hassager C, Helqvist S, Kelbæk H, Jørgensen E, Køber L, Bundgaard H. Survival and sudden cardiac death after septal ablation forhypertrophic obstructive cardiomyopathy. Scand Cardiovasc J. 2011Jun;45(3):153-60. doi: 10.3109/14017431.2011.565793. Epub 2011 Mar 29. PubMedPMID: 21604920.

142: Jensen MK, Almaas VM, Jacobsson L, Hansen PR, Havndrup O, Aakhus S, Svane B,Hansen TF, Køber L, Endresen K, Eriksson MJ, Jørgensen E, Amlie JP, Gadler F,Bundgaard H. Long-term outcome of percutaneous transluminal septal myocardialablation in hypertrophic obstructive cardiomyopathy: a Scandinavian multicenterstudy. Circ Cardiovasc Interv. 2011 Jun;4(3):256-65. doi: 10.1161/CIRCINTERVENTIONS.110.959718. Epub 2011 May 3. PubMed PMID: 21540441.

143: Tao YK, Yan LR, Li YS, Xu ZM, Zhang GQ, Zu LM, DU HY, Fan CM. [Clinicalfeatures and prognosis of 188 Chinese patients with apical hypertrophiccardiomyopathy]. Zhonghua Xin Xue Guan Bing Za Zhi. 2011 Feb;39(2):106-9.Chinese. PubMed PMID: 21426741.

144: Chen CC, Lei MH, Hsu YC, Chung SL, Sung YJ. Apical hypertrophiccardiomyopathy: correlations between echocardiographic parameters, angiographicleft ventricular morphology, and clinical outcomes. Clin Cardiol. 2011Apr;34(4):233-8. doi: 10.1002/clc.20874. Epub 2011 Mar 13. PubMed PMID: 21400548.

145: Hickey EJ, Mehta R, Elmi M, Asoh K, McCrindle BW, Williams WG, Manlhiot C,Benson L. Survival implications: hypertrophic cardiomyopathy in Noonan syndrome. Congenit Heart Dis. 2011 Jan-Feb;6(1):41-7. doi:10.1111/j.1747-0803.2010.00465.x. PubMed PMID: 21269411.

146: Hagège A. [Cardiac manifestations of Anderson-Fabry disease and efficacy of enzyme replacement therapy]. Rev Med Interne. 2010 Dec;31 Suppl 2:S238-42. doi:10.1016/S0248-8663(10)70020-5. French. PubMed PMID: 21211672.

147: Boufidou A, Mantziari L, Paraskevaidis S, Karvounis H, Nenopoulou E, ManthouME, Styliadis IH, Parcharidis G. An interesting case of cardiac amyloidosisinitially diagnosed as hypertrophic cardiomyopathy. Hellenic J Cardiol. 2010Nov-Dec;51(6):552-7. PubMed PMID: 1169191.

148: Samad Z, Kaul P, Shaw LK, Glower DD, Velazquez EJ, Douglas PS, Jollis JG.Impact of early surgery on survival of patients with severe mitral regurgitation.Heart. 2011 Feb;97(3):221-4. doi: 10.1136/hrt.2010.202432. Epub 2010 Nov 11.PubMed PMID: 21071750.

149: Payne J, Luis Fuentes V, Boswood A, Connolly D, Koffas H, Brodbelt D.Population characteristics and survival in 127 referred cats with hypertrophiccardiomyopathy (1997 to 2005). J Small Anim Pract. 2010 Oct;51(10):540-7. doi:10.1111/j.1748-5827.2010.00989.x. PubMed PMID: 21029098.

150: Fesslova V, Mongiovì M, Pipitone S, Brankovic J, Villa L. Features andoutcomes in utero and after birth of fetuses with myocardial disease. Int JPediatr. 2010;2010:628451. doi: 10.1155/2010/628451. Epub 2010 Oct 3. PubMedPMID: 20976307; PubMed Central PMCID: MC2952816.

151: Berruezo A, Vatasescu R, Mont L, Sitges M, Perez D, Papiashvili G, Vidal B, Francino A, Fernández-Armenta J, Silva E, Bijnens B, González-Juanatey JR,Brugada J. Biventricular pacing in hypertrophic obstructive cardiomyopathy: apilot study. Heart Rhythm. 2011 Feb;8(2):221-7. doi: 10.1016/j.hrthm.2010.10.010.Epub 2010 Oct 14. Erratum in: Heart Rhythm. 2011 Jun;8(6):953. Papiashvilli, Giorgi [corrected to Papiashvili, Giorgi]. PubMed PMID: 20951231.

152: Dimitrow PP, Chojnowska L, Rudzinski T, Piotrowski W, Ziólkowska L,Wojtarowicz A, Wycisk A, Dabrowska-Kugacka A, Nowalany-Kozielska E, Sobkowicz B, Wróbel W, Aleszewicz-Baranowska J, Rynkiewicz A, Loboz-Grudzien K, Marchel M,Wysokinski A. Sudden death in hypertrophic cardiomyopathy: old risk factorsre-assessed in a new model of maximalized follow-up. Eur Heart J. 2010Dec;31(24):3084-93. doi: 10.1093/eurheartj/ehq308. Epub 2010 Sep 15. PubMed PMID:20843960.

153: Brown ML, Schaff HV, Dearani JA, Li Z, Nishimura RA, Ommen SR. Relationship between left ventricular mass, wall thickness, and survival after subaorticseptal myectomy for hypertrophic obstructive cardiomyopathy. J Thorac Cardiovasc Surg. 2011 Feb;141(2):439-43. doi: 10.1016/j.jtcvs.2010.04.046. Epub 2010 Sep 15.PubMed PMID: 20832083.

154: Hamdan MA, El-Zoabi BA, Begam MA, Mirghani HM, Almalik MH. Antenataldiagnosis of pompe disease by fetal echocardiography: impact on outcome afterearly initiation of enzyme replacement therapy. J Inherit Metab Dis. 2010 Dec;33 Suppl 3:S333-9. doi: 10.1007/s10545-010-9179-2. Epub 2010 Sep 4. PubMed PMID:20821053.

155: Maron MS, Kalsmith BM, Udelson JE, Li W, DeNofrio D. Survival after cardiac transplantation in patients with hypertrophic cardiomyopathy. Circ Heart Fail.2010 Sep;3(5):574-9. doi: 10.1161/CIRCHEARTFAILURE.109.922872. Epub 2010 Aug 24. PubMed PMID: 20736440.

156: O'Hanlon R, Grasso A, Roughton M, Moon JC, Clark S, Wage R, Webb J, KulkarniM, Dawson D, Sulaibeekh L, Chandrasekaran B, Bucciarelli-Ducci C, Pasquale F,Cowie MR, McKenna WJ, Sheppard MN, Elliott PM, Pennell DJ, Prasad SK. Prognostic significance of myocardial fibrosis in hypertrophic cardiomyopathy. J Am CollCardiol. 2010 Sep 7;56(11):867-74. doi: 10.1016/j.jacc.2010.05.010. Epub 2010 Jun25. PubMed PMID: 20688032.

157: Choi JO, Yu CW, Chun Nah J, Rang Park J, Lee BS, Jeong Choi Y, Cho BR, LeeSC, Woo Park S, Kimura A, Euy Park J. Long-term outcome of 4 Korean families withhypertrophic cardiomyopathy caused by 4 different mutations. Clin Cardiol. 2010Jul;33(7):430-8. doi: 10.1002/clc.20795. PubMed PMID: 20641121.

158: Hamada T, Kubo T, Kitaoka H, Hirota T, Hoshikawa E, Hayato K, Shimizu Y,Okawa M, Yamasaki N, Matsumura Y, Yabe T, Takata J, Doi YL. Clinical features of the dilated phase of hypertrophic cardiomyopathy in comparison with those ofdilated cardiomyopathy. Clin Cardiol. 2010 Jul;33(7):E24-8. doi:10.1002/clc.20533. PubMed PMID: 20641106.

159: Lyne JC, Kilpatrick T, Duncan A, Knight CJ, Sigwart U, Fox KM. Long-termfollow-up of the first patients to undergo transcatheter alcohol septal ablation.Cardiology. 2010;116(3):168-73. doi: 10.1159/000318307. Epub 2010 Jul 9. PubMedPMID: 20616549.

160: Salerno M, Kramer CM. Prognosis in hypertrophic cardiomyopathy withcontrast-enhanced cardiac magnetic resonance: the future looks bright. J Am Coll Cardiol. 2010 Sep 7;56(11):888-9. doi: 10.1016/j.jacc.2010.06.004. Epub 2010 Jul 2. PubMed PMID: 20598824; PubMed Central PMCID: PMC2943765.

161: Yacoub M, Olivotto I, Cecchi F. Hypertrophic cardiomyopathy at 50. JCardiovasc Transl Res. 2009 Dec;2(4):339-40. doi: 10.1007/s12265-009-9157-y. Epub2009 Dec 4. PubMed PMID: 20559992.

162: Gerstenfeld EP. Hypertrophic cardiomyopathy with midcavitary obstruction:another substrate for ventricular tachycardia? J Cardiovasc Electrophysiol. 2010 Sep;21(9):1000-1. doi: 10.1111/j.1540-8167.2010.01800.x. PubMed PMID: 20487115.

163: Furushima H, Chinushi M, Iijima K, Sanada A, Izumi D, Hosaka Y, Aizawa Y.Ventricular tachyarrhythmia associated with hypertrophic cardiomyopathy:incidence, prognosis, and relation to type of hypertrophy. J CardiovascElectrophysiol. 2010 Sep;21(9):991-9. doi: 10.1111/j.1540-8167.2010.01769.x.PubMed PMID: 20487113.

164: Efthimiadis GK, Parcharidou D, Pagourelias ED, Meditskou S, Spanos G,Hadjimiltiades S, Pliakos C, Gavrielides S, Karvounis H, Styliadis IH,Parcharidis GE. Prevalence and clinical outcomes of incidentally diagnosedhypertrophic cardiomyopathy. Am J Cardiol. 2010 May 15;105(10):1445-50. doi:10.1016/j.amjcard.2009.12.066. Epub 2010 Mar 30. PubMed PMID: 20451692.

165: Aslam F, Haque A, Foody J, Shirani J. The frequency and functional impact ofoverlapping hypertension on hypertrophic cardiomyopathy: a single-centerexperience. J Clin Hypertens (Greenwich). 2010 Apr;12(4):240-5. doi:10.1111/j.1751-7176.2009.00251.x. PubMed PMID: 0433544.

166: Lin MH, Chou NK, Chen YS, Chi NH, Ko WJ, Yu HY, Huang SC, Wu NT, Wang JK, WuMH, Wang SS. Outcome in children bridged and nonbridged to cardiactransplantation. Transplant Proc. 2010 Apr;42(3):916-9. doi:10.1016/j.transproceed.2010.02.052. PubMed PMID: 0430203.

167: ten Cate FJ, Soliman OI, Michels M, Theuns DA, de Jong PL, Geleijnse ML,Serruys PW. Long-term outcome of alcohol septal ablation in patients withobstructive hypertrophic cardiomyopathy: a word of caution. Circ Heart Fail. 2010May;3(3):362-9. doi: 0.1161/CIRCHEARTFAILURE.109.862359. Epub 2010 Mar 23.PubMed PMID: 20332420.

168: Pinamonti B, Merlo M, Nangah R, Korcova R, Di Lenarda A, Barbati G, Sinagra G. The progression of left ventricular systolic and diastolic dysfunctions inhypertrophic cardiomyopathy: clinical and prognostic significance. J CardiovascMed (Hagerstown). 2010 Sep;11(9):669-77. doi: 10.2459/JCM.0b013e3283383355.PubMed PMID: 20216227.

169: Syska P, Przybylski A, Chojnowska L, Lewandowski M, Sterliński M, Maciag A, Gepner K, Pytkowski M, Kowalik I, Maczyńska-Mazuruk R, Ruzyłło W, Szwed H.Implantable cardioverter-defibrillator in patients with hypertrophiccardiomyopathy: efficacy and complications of the therapy in long-term follow-up.J Cardiovasc Electrophysiol. 2010 Aug 1;21(8):883-9. doi:10.1111/j.1540-8167.2009.01716.x. Epub 2010 Feb 1. PubMed PMID: 20132378.

170: Heffernan KS, Napolitano CA, Maron MS, Patvardhan EA, Patel AR, Pandian NG, Karas RH, Kuvin JT. Peripheral vascular endothelial function in patients withhypertrophic cardiomyopathy. Am J Cardiol. 2010 Jan 1;105(1):112-5. doi:10.1016/j.amjcard.2009.08.658. PubMed PMID: 20102901; PubMed Central PMCID:PMC2919165.

171: Wei BQ, Yang YJ, Zhang J, Dou KF, Zhang YH, Huang XH, Kang LM, Zhang CL, Gu Q, Gao X, Yang YM, Dai Y, Yu LT, Zhang HM, Lü R. [Predictive value of admissionamino-terminal pro-B-type natriuretic peptide on in-hospital mortality inpatients with decompensated heart failure]. Zhonghua Xin Xue Guan Bing Za Zhi.2009 Jun;37(6):481-5. Chinese. PubMed PMID: 19927625.

172: Rankin JS, Burrichter CA, Walton-Shirley MK, Whiteside JH, Teague SM,McLaughlin VW, Sharma MK, Johnston TS, McRae AT, Myers PR. Trends in mitral valvesurgery: a single practice experience. J Heart Valve Dis. 2009 Jul;18(4):359-66. PubMed PMID: 19852138.

173: Kubo T, Kitaoka H, Okawa M, Hirota T, Hoshikawa E, Hayato K, Yamasaki N,Matsumura Y, Yabe T, Nishinaga M, Takata J, Doi YL. Clinical profiles ofhypertrophic cardiomyopathy with apical phenotype--comparison of pure-apical formand distal-dominant form. Circ J. 2009 Dec;73(12):2330-6. Epub 2009 Oct 17.PubMed PMID: 19838003.

174: Goda A, Yamashita T, Suzuki S, Ohtsuka T, Uejima T, Oikawa Y, Yajima J,Koike A, Nagashima K, Kirigaya H, Sagara K, Ogasawara K, Isobe M, Sawada H,Aizawa T. Prevalence and prognosis of patients with heart failure in Tokyo: aprospective cohort of Shinken Database 2004-5. Int Heart J. 2009Sep;50(5):609-25. PubMed PMID: 19809210.

175: Dearani JA, Schaff HV, Ommen SR. [Septal myectomy for obstructivehypertrophic cardiomyopathy: the gold standard]. Zhonghua Xin Xue Guan Bing ZaZhi. 2009 Apr;37(4):307-11. Chinese. PubMed PMID: 19791464.

176: Olivotto I, DiDonna P, Baldi M, Sgalambro A, Maron BJ, Cecchi F. [Atrialfibrillation in hypertrophic cardiomyopathy: determinants, clinical course andmanagement]. Zhonghua Xin Xue Guan Bing Za Zhi. 2009 Apr;37(4):303-7. Chinese.PubMed PMID: 19791463.

177: Nemes A, Balázs E, Soliman OI, Sepp R, Csanády M, Forster T. Long-termprognostic value of coronary flow velocity reserve in patients with hypertrophic cardiomyopathy: 9-year follow-up results from SZEGED study. Heart Vessels. 2009Sep;24(5):352-6. doi: 10.1007/s00380-008-1131-0. Epub 2009 Sep 27. PubMed PMID:19784818.

178: Gajarski R, Naftel DC, Pahl E, Alejos J, Pearce FB, Kirklin JK, Zamberlan M,Dipchand AI; Pediatric Heart Transplant Study Investigators.. Outcomes ofpediatric patients with hypertrophic cardiomyopathy listed for transplant. JHeart Lung Transplant. 2009 Dec;28(12):1329-34. doi:

10.1016/j.healun.2009.05.028. Epub 2009 Sep 26. PubMed PMID: 19782603.179: Dipchand AI, Naftel DC, Feingold B, Spicer R, Yung D, Kaufman B, Kirklin JK,Allain-Rooney T, Hsu D; Pediatric Heart Transplant Study Investigators.. Outcomesof children with cardiomyopathy listed for transplant: a multi-institutionalstudy. J Heart Lung Transplant. 2009 Dec;28(12):1312-21. doi:10.1016/j.healun.2009.05.019. Epub 2009 Sep 26. PubMed PMID: 19782592.

180: Sorajja P, Nishimura RA, Gersh BJ, Dearani JA, Hodge DO, Wiste HJ, Ommen SR.Outcome of mildly symptomatic or asymptomatic obstructive hypertrophiccardiomyopathy: a long-term follow-up study. J Am Coll Cardiol. 2009 Jul14;54(3):234-41. doi: 10.1016/j.jacc.2009.01.079. PubMed PMID: 19589436.

181: Maron BJ, Haas TS, Shannon KM, Almquist AK, Hodges JS. Long-term survivalafter cardiac arrest in hypertrophic cardiomyopathy. Heart Rhythm. 2009Jul;6(7):993-7. doi: 10.1016/j.hrthm.2009.03.014. Epub 2009 Mar 11. PubMed PMID: 19497790.

182: Delmo Walter EM, Siniawski H, Hetzer R. Sustained improvement after combinedanterior mitral valve leaflet retention plasty and septal myectomy in preventing systolic anterior motion in hypertrophic obstructive cardiomyopathy in children. Eur J Cardiothorac Surg. 2009 ep;36(3):546-52. doi: 10.1016/j.ejcts.2009.03.049.Epub 2009 May 22. PubMed PMID: 19464922.

183: Fernandes VL, Nielsen C, Nagueh SF, Herrin AE, Slifka C, Franklin J, SpencerWH 3rd. Follow-up of alcohol septal ablation for symptomatic hypertrophicobstructive cardiomyopathy the Baylor and Medical University of South Carolinaexperience 1996 to 2007. JACC Cardiovasc Interv. 2008 Oct;1(5):561-70. doi:10.1016/j.jcin.2008.07.005. PubMed PMID: 19463359.

184: Kwon DH, Kapadia SR, Tuzcu EM, Halley CM, Gorodeski EZ, Curtin RJ,Thamilarasan M, Smedira NG, Lytle BW, Lever HM, Desai MY. Long-term outcomes inhigh-risk symptomatic patients with hypertrophic cardiomyopathy undergoingalcohol septal ablation. JACC Cardiovasc Interv. 2008 Aug;1(4):432-8. doi:10.1016/j.jcin.2008.05.009. PubMed PMID: 19463341.

185: Velazquez-Ceceña JL, Lubell DL, Nagajothi N, Al-Masri H, Siddiqui M, Khosla S. Syncope from dynamic left ventricular outflow tract obstruction simulatinghypertrophic cardiomyopathy in a patient with primary AL-type amyloid heartdisease. Tex Heart Inst J. 2009;36(1):50-4. PubMed PMID: 19436787; PubMed CentralPMCID: PMC2676515.

186: Arteaga E, de Araújo AQ, Bernstein M, Ramires FJ, Ianni BM, Fernandes F,Mady C. Prognosticvalue of the collagen volume fraction in hypertrophiccardiomyopathy. Arq Bras Cardiol. 2009 Mar;92(3):210-4, 216-20. English, Spanish.PubMed PMID: 19390710.

187: Maron BJ, Roberts WC, Arad M, Haas TS, Spirito P, Wright GB, Almquist AK,Baffa JM, Saul JP, Ho CY, Seidman J, Seidman CE. Clinical outcome and phenotypic expression in LAMP2 cardiomyopathy. JAMA. 2009 Mar 25;301(12):1253-9. doi:

10.1001/jama.2009.371. PubMed PMID: 19318653; PubMed Central PMCID: PMC4106257.

188: Spirito P, Autore C, Rapezzi C, Bernabò P, Badagliacca R, Maron MS,Bongioanni S, Coccolo F, Estes NA, Barillà CS, Biagini E, Quarta G, Conte MR,Bruzzi P, Maron BJ. Syncope and risk of sudden death in hypertrophiccardiomyopathy. Circulation. 2009 Apr 7;119(13):1703-10. doi:

10.1161/CIRCULATIONAHA.108.798314. Epub 2009 Mar 23. PubMed PMID: 19307481.

189: Kahveci G, Bayrak F, Mutlu B, Başaran Y. [Importance of left atrial size in predicting adverse clinical events in hypertrophic cardiomyopathy]. Turk KardiyolDern Ars. 2008 Dec;36(8):541-5. Turkish. PubMed PMID: 19223720.190: Diaz T, Pencina MJ, Benjamin EJ, Aragam J, Fuller DL, Pencina KM, Levy D,Vasan RS. Prevalence, clinical correlates, and prognosis of discrete upper septal

thickening on echocardiography: the Framingham Heart Study. Echocardiography.2009 Mar;26(3):247-53. doi: 10.1111/j.1540-8175.2008.00806.x. Epub 2008 Oct 23.PubMed PMID: 19175779; PubMed Central PMCID: PMC2657181.

191: Uchiyama K, Hayashi K, Fujino N, Konno T, Sakamoto Y, Sakata K, KawashiriMA, Ino H, Yamagishi M. Impact of QT variables on clinical outcome of genotypedhypertrophic cardiomyopathy. Ann Noninvasive Electrocardiol. 2009

Jan;14(1):65-71. doi: 10.1111/j.1542-474X.2008.00275.x. Erratum in: AnnNoninvasive Electrocardiol. 2009 Apr;14(2):218. PubMed PMID: 19149795.

192: Losi MA, Betocchi S, Barbati G, Parisi V, Tocchetti CG, Pastore F, Migliore T, Contaldi C, Caputi A, Romano R, Chiariello M. Prognostic significance of left atrial volume dilatation in patients with hypertrophic cardiomyopathy. J Am SocEchocardiogr. 2009 Jan;22(1):76-81. doi: 10.1016/j.echo.2008.11.001. PubMed PMID:19131006.

193: Balaram SK, Tyrie L, Sherrid MV, Afthinos J, Hillel Z, Winson G, Swistel DG.Resection-plication-release for hypertrophic cardiomyopathy: clinical andechocardiographic follow-up. Ann Thorac Surg. 2008 Nov;86(5):1539-44; discussion 1544-5. doi: 10.1016/j.athoracsur.2008.07.048. PubMed PMID: 19049745.

194: Cuoco FA, Spencer WH 3rd, Fernandes VL, Nielsen CD, Nagueh S, Sturdivant JL,Leman RB, Wharton JM, Gold MR. Implantable cardioverter-defibrillator therapy forprimary prevention of sudden death after alcohol septal ablation of hypertrophic cardiomyopathy. J Am Coll Cardiol. 2008 Nov 18;52(21):1718-23. doi:10.1016/j.jacc.2008.07.061. PubMed PMID: 19007692.

195: Hauser RG, Maron BJ, Marine JE, Lampert R, Kadish AH, Winters SL, Scher DL, Biria M, Kalia A. Safety and efficacy of transvenous high-voltage implantablecardioverter-defibrillator leads in high-risk hypertrophic cardiomyopathypatients. Heart Rhythm. 2008 Nov;5(11):1517-22. doi: 10.1016/j.hrthm.2008.08.021.Epub 2008 Aug 28. PubMed PMID: 18984525.

196: Yerebakan C, Buz S, Huebler M, Weng Y, Lehmkuhl H, Hetzer R. Rightventricular failure following heart transplantation--recovery after extendedmechanical support. J Card Surg. 2008 Sep-Oct;23(5):578-80. doi:10.1111/j.1540-8191.2008.00698.x. Erratum in: J Card Surg. 2008

Nov-Dec;23(6):799. Weng, Yug [corrected to Weng, Yuguo]. PubMed PMID: 18928498.

197: Dionigi B, Razzouk AJ, Hasaniya NW, Chinnock RE, Bailey LL. Late outcomes ofpediatric heart transplantation are independent of pre-transplant diagnosis andprior cardiac surgical intervention. J Heart Lung Transplant. 2008Oct;27(10):1090-5. doi: 10.1016/j.healun.2008.07.003. PubMed PMID: 18926399.

198: Genchi C, Venco L, Ferrari N, Mortarino M, Genchi M. Feline heartworm(Dirofilaria immitis) infection: a statistical elaboration of the duration of theinfection and life expectancy in asymptomatic cats. Vet Parasitol. 2008 Dec10;158(3):177-82. doi: 10.1016/j.vetpar.2008.09.005. Epub 2008 Sep 7. PubMedPMID: 18922638.

199: Welge D, Seggewiss H, Fassbender D, Schmidt HK, Horstkotte D, Faber L.[Long-term follow-up after percutaneous septal ablation in hypertrophicobstructive cardiomyopathy]. Dtsch Med Wochenschr. 2008 Sep;133(39):1949-54.doi: 10.1055/s-0028-1085601. Epub 2008 Sep 16. German. PubMed PMID: 18798130.

200: Faber L, Welge D, Hering D, Butz T, Oldenburg O, Seggewiss H, Horstkotte D. Percutaneous septal ablation after unsuccessful surgical myectomy for patientswith hypertrophic obstructive cardiomyopathy. Clin Res Cardiol. 2008Dec;97(12):899-904. doi: 10.1007/s00392-008-0707-0. Epub 2008 Sep 5. PubMed PMID:18777001.

201: Hage FG, Karakus G, Luke WD Jr, Suwanjutah T, Burri MV, Nanda NC, Aqel RA.Effect of alcohol-induced septal ablation on left atrial volume and ejectionfraction assessed by real time three-dimensional transthoracic echocardiographyin patients with hypertrophic cardiomyopathy. Echocardiography. 2008Aug;25(7):784-9. doi: 10.1111/j.1540-8175.2008.00735.x. Errtum in:

Echocardiography. 2008 Oct;25(9):ii. Burri, Manjula V [added]. PubMed PMID:18754938.

202: Buja P, Zuin G, Di Pede F, Madalosso M, Grassi G, Celestre M, Millosevich P,Rigo F, Raviele A. Long-term outcome and sex distribution across ages of leftventricular apical ballooning syndrome. J Cardiovasc Med (Hagerstown). 2008Sep;9(9):905-9. doi: 10.2459/JCM.0b013e3282fec072. PubMed PMID: 18695427.

203: Badertscher A, Bauersfeld U, Arbenz U, Baumgartner MR, Schinzel A, Balmer C.Cardiomyopathy in newborns and infants: a broad spectrum of aetiologies and poor prognosis. Acta Paediatr. 2008 Nov;97(11):1523-8. doi:10.1111/j.1651-2227.2008.00957.x. Epub 2008 Jul 22. PubMed PMID: 18652581.

204: Sorajja P, Valeti U, Nishimura RA, Ommen SR, Rihal CS, Gersh BJ, Hodge DO,Schaff HV, Holmes DR Jr. Outcome of alcohol septal ablation for obstructivehypertrophic cardiomyopathy. Circulation. 2008 Jul 8;118(2):131-9. doi:10.1161/CIRCULATIONAHA.107.738740. PubMed PMID: 18591440.

205: Roldán V, Marín F, Gimeno JR, Ruiz-Espejo F, González J, Feliu E,García-Honrubia A, Saua D, de la Morena G, Valdés M, Vicente V. Matrixmetalloproteinases and tissue remodeling in hypertrophic cardiomyopathy. Am HeartJ. 2008 Jul;156(1):85-91. doi: 10.1016/j.ahj.2008.01.035. PubMed PMID: 18585501.

206: Dion RA. Mitral valve abnormalities in hypertrophic cardiomyopathy:echocardiographic features and surgical outcomes. Invited commentary. Ann Thorac Surg. 2008 May;85(5):1536. doi: 10.1016/j.athoracsur.2008.02.044. PubMed PMID:18442533.

207: Kaple RK, Murphy RT, DiPaola LM, Houghtaling PL, Lever HM, Lytle BW,Blackstone EH, Smedira NG. Mitral valve abnormalities in hypertrophiccardiomyopathy: echocardiographic features and surgical outcomes. Ann ThoracSurg. 2008 May;85(5):1527-35, 1535.e1-2. doi: 10.1016/j.athoracsur.2008.01.061.PubMed PMID: 18442532.

208: van der Lee C, Scholzel B, ten Berg JM, Geleijnse ML, Idzerda HH, vanDomburg RT, Vletter WB, Serruys PW, ten Cate FJ. Usefulness of clinical,echocardiographic, and procedural characteristics to predict outcome afterpercutaneous transluminal septal myocardial ablation. Am J Cardiol. 2008 May1;101(9):1315-20. doi: 10.1016/j.amjcard.2008.01.003. Epub 2008 Mar 10. PubMedPMID: 18435964.

209: Wang S, Zou Y, Fu C, Xu X, Wang J, Song L, Wang H, Chen J, Wang J, Huan T,Hui R. Worse prognosis with gene mutations of beta-myosin heavy chain thanmyosin-binding protein C in Chinese patients with hypertrophic cardiomyopathy.Clin Cardiol. 2008 Mar;31(3):114-8. doi: 10.1002/clc.20151. PubMed PMID:18383048.

210: Li M, Wang QB, Cheng K. [Long term follow-up results of 199 patients withhypertrophic cardiomyopathy]. Zhonghua Xin Xue Guan Bing Za Zhi. 2007Nov;35(11):988-91. Chinese. PubMed PMID: 18269816.

211: Brown ML, Schaff HV. Surgical management of hypertrophic cardiomyopathy in2007: what is new? World J Surg. 2008 Mar;32(3):350-4. doi:10.1007/s00268-007-9412-9. PubMed PMID: 18202887.

212: Vural AH, Tiryakioğlu O, Türk T, Ata Y, Ari H, Yalçinkaya S, Erkut B, Bozat T, Ozyazicioğlu A. Treatment modalities in hypertrophic obstructivecardiomyopathy: surgical myectomy versus percutaneous septal ablation. Heart SurgForum. 2007;10(6):493-7. doi: 10.1532/HSF98.20071136. PubMed PMID: 18187386.

213: Smedira NG, Lytle BW, Lever HM, Rajeswaran J, Krishnaswamy G, Kaple RK,Dolney DO, Blackstone EH. Current effectiveness and risks of isolated septalmyectomy for hypertrophic obstructive cardiomyopathy. Ann Thorac Surg. 2008Jan;85(1):127-33. PubMed PMID: 18154797.

214: Nagamatsu H, Momose M, Kobayashi H, Kusakabe K, Kasanuki H. Prognostic valueof 123I-metaiodobenzylguanidine in patients with various heart diseases. Ann NuclMed. 2007 Nov;21(9):513-20. Epub 2007 Nov 26. PubMed PMID: 18030583.

215: Refaat M. Prognosis of apical hypertrophic cardiomyopathy. JAMA. 2007 Nov7;298(17):2006; author reply 2006. PubMed PMID: 17986692.

216: Xin B, Puffenberger E, Tumbush J, Bockoven JR, Wang H. Homozygosity for anovel splice site mutation in the cardiac myosin-binding protein C gene causessevere neonatal hypertrophic cardiomyopathy. Am J Med Genet A. 2007 Nov15;143A(22):2662-7. PubMed PMID: 17937428.

217: Faber L, Welge D, Fassbender D, Schmidt HK, Horstkotte D, Seggewiss H.One-year follow-up of percutaneous septal ablation for symptomatic hypertrophicobstructive cardiomyopathy in 312 patients: predictors of hemodynamic andclinical response. Clin Res Cardiol. 2007 Dec;96(12):864-73. Epub 2007 Sep 25.PubMed PMID: 17891518.

218: Seggewiss H, Rigopoulos A, Welge D, Ziemssen P, Faber L. Long-term follow-upafter percutaneous septal ablation in hypertrophic obstructive cardiomyopathy.Clin Res Cardiol. 2007 Dec;96(12):856-63. Epub 2007 Sep 27. PubMed PMID:17891517.

219: Rosso R, Kalman JM, Rogowski O, Diamant S, Birger A, Biner S, Belhassen B,Viskin S. Calcium channel blockers and beta-blockers versus beta-blockers alonefor preventing exercise-induced arrhythmias in catecholaminergic polymorphicventricular tachycardia. Heart Rhythm. 2007 Sep;4(9):1149-54. Epub 2007 May 24.PubMed PMID: 17765612.

220: Nagueh SF, Buergler JM, Quinones MA, Spencer WH 3rd, Lawrie GM. Outcome ofsurgical myectomy after unsuccessful alcohol septal ablation for the treatment ofpatients with hypertrophic obstructive cardiomyopathy. J Am Coll Cardiol. 2007Aug 21;50(8):795-8. Epub 2007 Aug 6. PubMed PMID: 17707185.

221: Bayrak F, Kahveci G, Mutlu B, Değertekin M, Demirtaş E. Usefulness ofsurface electrocardiogram in predicting the clinical course of patients withhypertrophic cardiomyopathy. Anadolu Kardiyol Derg. 2007 Jul;7 Suppl 1:178-81.PubMed PMID: 17584718.

222: Bongioanni S, Bianchi F, Migliardi A, Gnavi R, Pron PG, Casetta M, Conte MR.Relation of QRS duration to mortality in a community-based cohort withhypertrophic cardiomyopathy. Am J Cardiol. 2007 Aug 1;100(3):503-6. Epub 2007 Jun13. PubMed PMID: 17659936.

223: Kubo T, Gimeno JR, Bahl A, Steffensen U, Steffensen M, Osman E, Thaman R,Mogensen J, Elliott PM, Doi Y, McKenna WJ. Prevalence, clinical significance, andgenetic basis of hypertrophic cardiomyopathy with restrictive phenotype. J AmColl Cardiol. 2007 Jun 26;49(25):2419-26. Epub 2007 Jun 11. PubMed PMID:17599605.

224: Efthimiadis GK, Giannakoulas G, Parcharidou DG, Karvounis HI, Mochlas ST,Styliadis IH, Papadopoulos CE, Kounatiadis P, Pliakos CI, Parcharidis GE,Louridas GE. Clinical significance of tissue Doppler imaging in patients withhypertrophic cardiomyopathy. Circ J. 2007 Jun;71(6):897-903. PubMed PMID:17526987.

225: Ohba M, Hosokawa R, Kambara N, Tadamura E, Mamede M, Kubo S, Yamamuro M,Fujita M, Kimura T, Nohara R, Kita T. Difference in myocardial flow reservebetween patients with dilated cardiomyopathy and those with dilated phase ofhypertrophic cardiomyopathy: evaluation by 15O-water PET. Circ J. 2007Jun;71(6):884-90. PubMed PMID: 17526985.

226: Keren A, Poteckin M, Mazouz B, Medina A, Banai S, Chenzbraun A, Khoury Z,Levin G. Late in-hospital pressure gradient measurements improve prediction oflong-term outcome of alcohol septal ablation in hypertrophic cardiomyopathy. Isr Med Assoc J. 2007 Apr;9(4):239-42. PubMed PMID: 17491213.

227: da Fonseca SM, Belo LG, Carvalho H, Araújo N, Munhoz C, Siqueira L, MacielW, Andréa E, Atié J. Clinical follow-up of patients with implantablecardioverter-defibrillator. Arq Bras Cardiol. 2007 Jan;88(1):8-16. English,Portuguese. PubMed PMID: 17364112.

228: Cha YM, Gersh BJ, Maron BJ, Boriani G, Spirito P, Hodge DO, Weivoda PL,Trusty JM, Friedman PA, Hammill SC, Rea RF, Shen WK. Electrophysiologicmanifestations of ventricular tachyarrhythmias provoking appropriatedefibrillator interventions in high-risk patients with hypertrophiccardiomyopathy. J Cardiovasc Electrophysiol. 2007 May;18(5):483-7. Epub 2007 Mar 6. PubMed PMID: 17343723.

229: Kim SS, Knight BP. Defibrillator therapies in hypertrophic cardiomyopathy:too many swings at bad pitches? J Cardiovasc Electrophysiol. 2007May;18(5):488-9. Epub 2007 Mar 6. PubMed PMID: 17343717.

230: Heldman AW, Wu KC, Abraham TP, Cameron DE. Myectomy or alcohol septalablation surgery and percutaneous intervention go another round. J Am CollCardiol. 2007 Jan 23;49(3):358-60. Epub 2007 Jan 4. PubMed PMID: 17239718.

231: Valeti US, Nishimura RA, Holmes DR, Araoz PA, Glockner JF, Breen JF, OmmenSR, Gersh BJ, Tajik AJ, Rihal CS, Schaff HV, Maron BJ. Comparison of surgicalseptal myectomy and alcohol septal ablation with cardiac magnetic resonanceimaging in patients with hypertrophic obstructive cardiomyopathy. J Am CollCardiol. 2007 Jan 23;49(3):350-7. Epub 2007 Jan 4. PubMed PMID: 17239717.

232: Daebritz SH, Schmoeckel M, Mair H, Kozlik-Feldmann R, Wittmann G, KowalskiC, Kaczmarek I, Reichart B. Blood type incompatible cardiac transplantation inyoung infants. Eur J Cardiothorac Surg. 2007 Mar;31(3):339-43; discussion 343.Epub 2007 Jan 17. PubMed PMID: 17239612.

233: Finsterer J, Stöllberger C, Krugluger W. Positive troponin-T innoncompaction is associated with neuromuscular disorders and poor outcome. ClinRes Cardiol. 2007 Feb;96(2):109-13. Epub 2006 Dec 8. PubMed PMID: 17146603.

234: McMahon CJ, Pignatelli RH, Nagueh SF, Lee VV, Vaughn W, Valdes SO, KovalchinJP, Jefferies JL, Dreyer WJ, Denfield SW, Clunie S, Towbin JA, Eidem BW. Leftventricular non-compaction cardiomyopathy in children: characterisation ofclinical status using tissue Doppler-derived indices of left ventriculardiastolic relaxation. Heart. 2007 Jun;93(6):676-81. Epub 2006 Nov 29. Erratum in:Heart. 2007 Aug 1;93(8):984. Jefferies, J Lynn [corrected to Jefferies, John L]. PubMed PMID: 17135224; PubMed Central PMCID: PMC1955174.

235: Kitaoka H, Kubo T, Okawa M, Hitomi N, Furuno T, Doi YL. Left ventricularremodeling of hypertrophic cardiomyopathy: longitudinal observation in ruralcommunity. Circ J. 2006 Dec;70(12):1543-9. PubMed PMID: 17127796.

236: Navarro-López F. [Hypertrophic cardiomyopathy: never-ending complexity]. RevEsp Cardiol. 2006 Oct;59(10):994-6. Spanish. PubMed PMID: 17125707.

237: Nasermoaddeli A, Miura K, Matsumori A, Soyama Y, Morikawa Y, Kitabatake A,Inaba Y, Nakagawa H. Prognosis and prognostic factors in patients withhypertrophic cardiomyopathy in Japan: results from a nationwide study. Heart.2007 Jun;93(6):711-5. Epub 2006 Nov 3. PubMed PMID: 17085533; PubMed CentralPMCID: PMC1955214.

238: Faber L, Welge D, Fassbender D, Schmidt HK, Horstkotte D, Seggewiss H.Percutaneous septal ablation for symptomatic hypertrophic obstructivecardiomyopathy: managing the risk of procedure-related AV conductiondisturbances. Int J Cardiol. 2007 Jul 10;119(2):163-7. Epub 2006 Oct 24. PubMedPMID: 17067708.

239: Nistri S, Olivotto I, Betocchi S, Losi MA, Valsecchi G, Pinamonti B, ConteMR, Casazza F, Galderisi M, Maron BJ, Cecchi F. Prognostic significance of leftatrial size in patients with hypertrophic cardiomyopathy (from the ItalianRegistry for Hypertrophic Cardiomyopathy). Am J Cardiol. 2006 Oct 1;98(7):960-5. Epub 2006 Aug 14. PubMed PMID: 16996883.

240: Shaw AC, Kalidas K, Crosby AH, Jeffery S, Patton MA. The natural history of Noonan syndrome: a long-term follow-up study. Arch Dis Child. 2007Feb;92(2):128-32. Epub 2006 Sep 21. PubMed PMID: 16990350; PubMed Central PMCID: PMC2083343.

241: Kishnani PS, Nicolino M, Voit T, Rogers RC, Tsai AC, Waterson J, Herman GE, Amalfitano A, Thurberg BL, Richards S, Davison M, Corzo D, Chen YT. Chinesehamster ovary cell-derived recombinant human acid alpha-glucosidase ininfantile-onset Pompe disease. J Pediatr. 2006 Jul;149(1):89-97. PubMed PMID:16860134; PubMed Central PMCID: PMC2692727.

242: Drezner JA, Rogers KJ. Sudden cardiac arrest in intercollegiate athletes:detailed analysis and outcomes of resuscitation in nine cases. Heart Rhythm. 2006Jul;3(7):755-9. Epub 2006 Mar 28. PubMed PMID: 16818200.

243: Sorajja P, Nishimura RA, Ommen SR, Ackerman MJ, Tajik AJ, Gersh BJ. Use ofechocardiography in patients with hypertrophic cardiomyopathy: clinicalimplications of massive hypertrophy. J Am Soc Echocardiogr. 2006Jun;19(6):788-95. PubMed PMID: 16762758.

244: Lee CH, Liu PY, Lin LJ, Chen JH, Tsai LM. Clinical features and outcome ofpatients with apical hypertrophic cardiomyopathy in Taiwan. Cardiology.2006;106(1):29-35. Epub 2006 Apr 7. PubMed PMID: 16612066.

245: Obergassel L, Lawrenz T, Gietzen FH, Lieder F, Leuner C, Kuhn H, Stellbrink C. Effect of transcoronary ablation of septal hypertrophy on clinical outcome in hypertrophic obstructive cardiomyopathy associated with atrial fibrillation. ClinRes Cardiol. 2006 May;95(5):254-60. Epub 2006 Mar 21. PubMed PMID: 16598396.

246: Salemi VM, Rochitte CE, Lemos P, Benvenuti LA, Pita CG, Mady C. Long-termsurvival of a patient with isolated noncompaction of the ventricular myocardium. J Am Soc Echocardiogr. 2006 Mar;19(3):354.e1-354.e3. PubMed PMID: 16500501.

247: Sorajja P, Chareonthaitawee P, Ommen SR, Miller TD, Hodge DO, Gibbons RJ.Prognostic utility of single-photon emission computed tomography in adultpatients with hypertrophic cardiomyopathy. Am Heart J. 2006 Feb;151(2):426-35.PubMed PMID: 16442910.

248: D'Andrea A, Caso P, Severino S, Cuomo S, Capozzi G, Calabrò P, Cice G,Ascione L, Scherillo M, Calabrò R. Prognostic value of intra-left ventricularelectromechanical asynchrony in patients with hypertrophic cardiomyopathy. EurHeart J. 2006 Jun;27(11):1311-8. Epub 2005 Dec 19. PubMed PMID: 16364972.

249: Hassan WM, Fawzy ME, Al Helaly S, Hegazy H, Malik S. Pitfalls in diagnosisand clinical, echocardiographic, and hemodynamic findings in endomyocardialfibrosis: a 25-year experience. Chest. 2005 Dec;128(6):3985-92. PubMed PMID:16354870.

250: Yang H, Woo A, Monakier D, Jamorski M, Fedwick K, Wigle ED, Rakowski H.Enlarged left atrial volume in hypertrophic cardiomyopathy: a marker for disease severity. J Am Soc Echocardiogr. 2005 Oct;18(10):1074-82. PubMed PMID: 16198885.

251: Maron BJ, Wentzel DC, Zenovich AG, Estes NA 3rd, Link MS. Death in a youngathlete due to commotio cordis despite prompt external defibrillation. HeartRhythm. 2005 Sep;2(9):991-3. PubMed PMID: 16171756.

252: Watkins H, McKenna WJ. The prognostic impact of septal myectomy inobstructive hypertrophic cardiomyopathy. J Am Coll Cardiol. 2005 Aug2;46(3):477-9. PubMed PMID: 16053961.

253: Ommen SR, Maron BJ, Olivotto I, Maron MS, Cecchi F, Betocchi S, Gersh BJ,Ackerman MJ, McCully RB, Dearani JA, Schaff HV, Danielson GK, Tajik AJ, NishimuraRA. Long-term effects of surgical septal myectomy on survival in patients withobstructive hypertrophic cardiomyopathy. J Am Coll Cardiol. 2005 Aug2;46(3):470-6. PubMed PMID: 16053960.

254: Faber L, Seggewiss H, Gietzen FH, Kuhn H, Boekstegers P, Neuhaus L, SeipelL, Horstkotte D. Catheter-based septal ablation for symptomatic hypertrophicobstructive cardiomyopathy: follow-up results of the TASH-registry of the German Cardiac Society. Z Kardiol. 2005 Aug;94(8):516-23. PubMed PMID: 16049653.

255: Gibson W, Trevenen C, Giuffre M, Leung AK. Noonan syndrome in a prematureinfant with hypertrophic cardiomyopathy and death in infancy. J Natl Med Assoc.2005 Jun;97(6):805-7. PubMed PMID: 16035579; PubMed Central PMCID: PMC2569491.

256: Montgomery JV, Harris KM, Casey SA, Zenovich AG, Maron BJ. Relation ofelectrocardiographic patterns to phenotypic expression and clinical outcome inhypertrophic cardiomyopathy. Am J Cardiol. 2005 Jul 15;96(2):270-5. PubMed PMID: 16018856.

257: Arteaga E, Ianni BM, Fernandes F, Mady C. Benign outcome in a long-termfollow-up of patients with hypertrophic cardiomyopathy in Brazil. Am Heart J.2005 Jun;149(6):1099-105. PubMed PMID: 15976794.

258: Thaman R, Gimeno JR, Murphy RT, Kubo T, Sachdev B, Mogensen J, Elliott PM,McKenna WJ. Prevalence and clinical significance of systolic impairment inhypertrophic cardiomyopathy. Heart. 2005 Jul;91(7):920-5. PubMed PMID: 15958362; PubMed Central PMCID: PMC1768999.

259: Sherrid MV, Barac I, McKenna WJ, Elliott PM, Dickie S, Chojnowska L, CaseyS, Maron BJ. Multicenter study of the efficacy and safety of disopyramide inobstructive hypertrophic cardiomyopathy. J Am Coll Cardiol. 2005 Apr19;45(8):1251-8. PubMed PMID: 15837258.

260: Lawrenz T, Obergassel L, Lieder F, Leuner C, Strunk-Mueller C, Meyer ZuVilsendorf D, Beer G, Kuhn H. Transcoronary ablation of septal hypertrophy doesnot alter ICD intervention rates in high risk patients with hypertrophicobstructive cardiomyopathy. Pacing Clin Electrophysiol. 2005 Apr;28(4):295-300.PubMed PMID: 15826262.

261: Woo A, Williams WG, Choi R, Wigle ED, Rozenblyum E, Fedwick K, Siu S,Ralph-Edwards A, Rakowski H. Clinical and echocardiographic determinants oflong-term survival after surgical myectomy in obstructive hypertrophiccardiomyopathy. Circulation. 2005 Apr 26;111(16):2033-41. Epub 2005 Apr 11.PubMed PMID: 15824202.

262: Dearani JA, Danielson GK. Septal myectomy for obstructive hypertrophiccardiomyopathy. Semin Thorac Cardiovasc Surg Pediatr Card Surg Annu. 2005:86-91. PubMed PMID: 15818363.

263: Fernandes VL, Nagueh SF, Wang W, Roberts R, Spencer WH 3rd. A prospectivefollow-up of alcohol septal ablation for symptomatic hypertrophic obstructivecardiomyopathy--the Baylor experience (1996-2002). Clin Cardiol. 2005Mar;28(3):124-30. PubMed PMID: 15813618.

264: Autore C, Bernabò P, Barillà CS, Bruzzi P, Spirito P. The prognosticimportance of left ventricular outflow obstruction in hypertrophic cardiomyopathyvaries in relation to the severity of symptoms. J Am Coll Cardiol. 2005 Apr5;45(7):1076-80. PubMed PMID: 15808767.

265: Thiam M, Ka S, Ndiaye R, Fall PD, Mbaye FK, Imbert P. [Hypertrophicobstructive cardiomyopathy in childAbout two cases]. Dakar Med.

2004;49(3):215-7. French. PubMed PMID: 15776621.

266: Gorlitzer M, Ankersmit J, Fiegl N, Meinhart J, Lanzenberger M, Unal K,Dunkler D, Kilo J, Wolner E, Grimm M, Grabenwoeger M. Is the transpulmonarypressure gradient a predictor for mortality after orthotopic cardiactransplantation? Transpl Int. 2005 Apr;18(4):390-5. PubMed PMID: 15773956.

267: Golovchiner G, Mazur A, Kogan A, Strasberg B, Shapira Y, Fridman M, Kuzniec J, Vidne BA, Raanani E. Atrial flutter after surgical radiofrequency ablation of the left atrium for atrial fibrillation. Ann Thorac Surg. 2005 Jan;79(1):108-12. PubMed PMID: 15620925.

268: Maleszka A, Kleikamp G, Koerfer R. Tricuspid valve replacement: clinicallong-term results for acquired isolated tricuspid valve regurgitation. J HeartValve Dis. 2004 Nov;13(6):957-61. PubMed PMID: 15597590.

269: Dörge H, Schmitto JD, Liakopoulos OJ, Walther S, Schöndube FA. Extendedmyectomy for hypertrophic obstructive cardiomyopathy after failure orcontraindication of septal ablation or with combined surgical procedures. Thorac Cardiovasc Surg. 2004 Dec;52(6):344-8. PubMed PMID: 15573275.

270: MacKenzie R. Tall R wave in lead V1. J Insur Med. 2004;36(3):255-9. PubMedPMID: 15495441.

271: Romero-Farina G, Candell-Riera J, Galve E, Armadans L, Ramos F, Castell J,Aguadé S, Nogales JM, Soler-Soler J. Do myocardial perfusion SPECT andradionuclide angiography studies in adult patients with hypertrophiccardiomyopathy have prognostic implications? J Nucl Cardiol. 2004Sep-Oct;11(5):578-86. PubMed PMID: 15472643.

272: Faber L, Seggewiss H, Welge D, Fassbender D, Schmidt HK, Gleichmann U,Horstkotte D. Echo-guided percutaneous septal ablation for symptomatichypertrophic obstructive cardiomyopathy: 7 years of experience. Eur JEchocardiogr. 2004 Oct;5(5):347-55. PubMed PMID: 15341870.

273: Murakami K, Shigematsu Y, Hamada M, Higaki J. Insulin resistance in patientswith hypertrophic cardiomyopathy. Circ J. 2004 Jul;68(7):650-5. PubMed PMID:15226630.

274: García Castro M, Rodríguez Reguero J. [Hypertrophic myocardiopathy: how topredict its evolution?]. Med Clin (Barc). 2004 Jun 5;123(1):17-8. Spanish. PubMedPMID: 15207222.

275: Candell-Riera J, Romero-Farina G, Galve E, Armadans L, Palet J,Castell-Conesa J. [Hypertrophic cardiomyopathy in adult patients. Clinical courseand prognostic factors in 119 patients]. Med Clin (Barc). 2004 Jun 5;123(1):1-4. Spanish. PubMed PMID: 15207219.

276: Biswas RG, Bandyopadhyay BK, Sarkar M, Sarkar UK, Goswami A, Mukherjee P.Perioperative management of pregnant patients with heart disease for caesariansection under anaesthesia. J Indian Med Assoc. 2003 Nov;101(11):632, 634, 636-7passim. PubMed PMID: 15198410.

277: Gietzen FH, Leuner CJ, Obergassel L, Strunk-Mueller C, Kuhn H. Transcoronaryablation of septal hypertrophy for hypertrophic obstructive cardiomyopathy:feasibility, clinical benefit, and short term results in elderly patients. Heart.2004 Jun;90(6):638-44. PubMed PMID: 15145866; PubMed Central PMCID: PMC1768263.

278: Boldt T, Andersson S, Eronen M. Etiology and outcome of fetuses withfunctional heart disease. Acta Obstet Gynecol Scand. 2004 Jun;83(6):531-5. PubMedPMID: 15144333.

279: Chen LW. [Orthotopic heart transplantation: a clinical analysis of 43patients]. Zhonghua Yi Xue Za Zhi. 2004 Apr 17;84(8):646-8. Chinese. PubMed PMID:15130304.

280: Bhudia SK, McCarthy PM, Smedira NG, Lam BK, Rajeswaran J, Blackstone EH.Edge-to-edge (Alfieri) mitral repair: results in diverse clinical settings. AnnThorac Surg. 2004 May;77(5):1598-606. PubMed PMID: 15111150.

281: Coutu M, Perrault LP, White M, Pelletier GB, Racine N, Poirier NC, CarrierM. Cardiac transplantation for hypertrophic cardiomyopathy: a valid therapeuticoption. J Heart Lung Transplant. 2004 Apr;23(4):413-7. PubMed PMID: 15063400.

282: Minakata K, Dearani JA, Nishimura RA, Maron BJ, Danielson GK. Extendedseptal myectomy for hypertrophic obstructive cardiomyopathy with anomalous mitralpapillary muscles or chordae. J Thorac Cardiovasc Surg. 2004 Feb;127(2):481-9.PubMed PMI: 14762358.

283: Sorajja P, Ommen SR, Nishimura RA, Gersh BJ, Berger PB, Tajik AJ. Adverseprognosis of patients with hypertrophic cardiomyopathy who have epicardialcoronary artery disease. Circulation. 2003 Nov 11;108(19):2342-8. Epub 2003 Oct27. PubMed PMID: 14581405.

284: Munclinger MJ. Recent developments in cardiac pacing. Cardiovasc J S Afr.2003 Jul-Aug;14(4):172-5. PubMed PMID: 14532957.

285: Colín Lizalde Lde J. [Hypertrophic cardiomyopathy. Arrhythmia inhypertrophic cardiomyopathy]. Arch Cardiol Mex. 2003 Apr-Jun;73 Suppl 1:S26-30.panish. PubMed PMID: 12966640.

286: Sorajja P, Ommen SR, Nishimura RA, Gersh BJ, Tajik AJ, Holmes DR. Myocardialbridging in adult patients with hypertrophic ardiomyopathy. J Am Coll Cardiol.2003 Sep 3;42(5):889-94. PubMed PMID: 12957438.

287: Maron BJ, Casey SA, Hauser RG, Aeppli DM. Clinical course of hypertrophiccardiomyopathy with survival to advanced age. J Am Coll Cardiol. 2003 Sep3;42(5):882-8. PubMed PMID: 12957437.

288: Bhagwandeen R, Woo A, Ross J, Wigle ED, Rakowski H, Kwinter J, Eriksson MJ, Schwartz L. Septal ethanol ablation for hypertrophic obstructive cardiomyopathy: early and intermediate results of a Canadian referral centre. Can J Cardiol. 2003Jul;19(8):912-7. PubMed PMID: 12876612.

289: Kawasaki T, Azuma A, Asada S, Hadase M, Kamitani T, Kawasaki S, Kuribayashi T, Sugihara H. Heart rate turbulence and clinical prognosis in hypertrophiccardiomyopathy and myocardial infarction. Circ J. 2003 Jul;67(7):601-4. PubMedPMI: 12845183.

290: Iqbal K, Tramboo NA, Mohi-Ud-Din K. Hypertrophic cardiomyopathy and outflow tract obstruction. N Engl J Med. 2003 May 1;348(18):1815-6; author reply 1815-6. PubMed PMID: 12728915.291: Monserrat L, Penas-Lado M, Castro-Beiras A. Hypertrophic cardiomyopathy and outflow tract obstruction. N Engl J Med. 2003 May 1;348(18):1815-6; author reply 815-6. PubMed PMID: 12724493.

292: Kofflard MJ, Ten Cate FJ, van der Lee C, van Domburg RT. Hypertrophiccardiomyopathy in a large community-based population: clinical outcome andidentification of risk factors for sudden cardiac death and clinicaldeterioration. J Am Coll Cardiol. 2003 Mar 19;41(6):987-93. PubMed PMID:12651046.

293: Maron BJ, Piccininno M, Casey SA, Bernabò P, Spirito P. Relation of extreme left ventricular hypertrophy to age in hypertrophic cardiomyopathy. Am J Cardiol.2003 Mar 1;91(5):626-8. PubMed PMID: 12615280.

294: Isobe N, Toyama T, Taniguchi K, Oshima S, Kubota S, Suzuki T, Nagaoka H,Adachi H, Naito S, Hoshizaki H. Failure to raise blood pressure during exerciseis a poor prognostic sign in patients with hypertrophic non-obstructivecardiomyopathy. Circ J. 2003 Mar;67(3):191-4. PubMed PMID: 12604864.

295: Holmgren D, Wåhlander H, Eriksson BO, Oldfors A, Holme E, Tulinius M.Cardiomyopathy in children with mitochondrial disease; clinical course andcardiological findings. Eur Heart J. 2003 Feb;24(3):280-8. PubMed PMID: 12590906.

296: Smith SA, Tobias AH, Jacob KA, Fine DM, Grumbles PL. Arterialthromboembolism in cats: acute crisis in 127 cases (1992-2001) and long-termmanagement with low-dose aspirin in 24 cases. J Vet Intern Med. 2003Jan-Feb;17(1):73-83. PubMed PMID: 12564730.

297: Hyodo HM, Unno N, Masuda H, Watanabe T, Kozuma S, Taketani Y. Myocardialhypertrophy of the recipient twins in twin-to-twin transfusion syndrome andcerebral palsy. Int J Gynaecol Obstet. 2003 Jan;80(1):29-34. PubMed PMID:12527457.

298: Mohiddin SA, Fananapazir L. Systolic compression of epicardial coronary and intramural arteries in children with hypertrophic cardiomyopathy. Tex Heart Inst J. 2002;29(4):290-8. PubMed PMID: 12484613; PubMed Central PMCID: PMC140291.

299: Autore C, Conte MR, Piccininno M, Bernabò P, Bonfiglio G, Bruzzi P, Spirito P. Risk associated with pregnancy in hypertrophic cardiomyopathy. J Am CollCardiol. 2002 Nov 20;40(10):1864-9. PubMed PMID: 12446072.

300: Wasant P, Matsumoto I, Naylor E, Liammongkolkul S. Mitochondrial fatty acid oxidation disorders in Thai infants: a report of 3 cases. J Med Assoc Thai. 2002 Aug;85 Suppl 2:S710-9. PubMed PMID: 12403251.

301: Stefanelli CB, Bradley DJ, Leroy S, Dick M 2nd, Serwer GA, Fischbach PS.Implantable cardioverter defibrillator therapy for life-threatening arrhythmiasin young patients. J Interv Card Electrophysiol. 2002 Jul;6(3):235-44. PubMedPMID: 12154326.

302: Minami K, Boethig D, Woltersdorf H, Seifert D, Körfer R. Long term follow-upof surgical treatment of hypertrophic obstructive cardiomyopathy (HOCM): the roleof concomitant cardiac procedures. Eur J Cardiothorac Surg. 2002Aug;22(2):206-10. PubMed PMID: 12142186.

303: Gietzen FH, Leuner CJ, Obergassel L, Strunk-Mueller C, Kuhn H. Role oftranscoronary ablation of septal hypertrophy in patients with hypertrophiccardiomyopathy, New York Heart Association functional class III or IV, andoutflow obstruction only under provocable conditions. Circulation. 2002 Jul23;106(4):454-9. PubMed PMID: 12135945.

304: Rush JE, Freeman LM, Fenollosa NK, Brown DJ. Population and survivalcharacteristics of cats with hypertrophic cardiomyopathy: 260 cases (1990-1999). J Am Vet Med Assoc. 2002 Jan 15;220(2):202-7. PubMed PMID: 12126131.

305: Bruno E, Maisuls H, Juaneda E, Moreyra E, Alday LE. Clinical features ofhypertrophic cardiomyopathy in the young. Cardiol Young. 2002 Mar;12(2):147-52.PubMed PMID: 12018719.

306: Takagi E, Yamakado T. Prognosis of patients with hypertrophic cardiomyopathyin Japan. Card Electrophysiol Rev. 2002 Feb;6(1-2):34-5. PubMed PMID: 11984013.

307: Chatrath R, Porter CB, Ackerman MJ. Role of transvenous implantablecardioverter-defibrillators in preventing sudden cardiac death in children,adolescents, and young adults. Mayo Clin Proc. 2002 Mar;77(3):226-31. PubMedPMID: 11888025.

308: Eriksson MJ, Sonnenberg B, Woo A, Rakowski P, Parker TG, Wigle ED, Rakowski H. Long-term outcome in patients with apical hypertrophic cardiomyopathy. J AmColl Cardiol. 2002 Feb 20;39(4):638-45. PubMed PMID: 11849863.

309: Fluri S, Gebbers JO. [Cor bovinum: decreased incidence over the last 20years--a therapeutic success?]. Praxis (Bern 1994). 2001 Nov 8;90(45):1964-72.German. PubMed PMID: 11817240.

310: Robinson BV, Ettedgui JA, Sherman FS. Use of terbutaline in the treatment ofcomplete heart block in the fetus. Cardiol Young. 2001 Nov;11(6):683-6. PubMedPMID: 11813927.

311: Rodrigus IE, Amsel BJ, Conraads V, De Hert S, Moulijn AC. Emergencyventricular assist device: better survival rates in non-post cardiotomy-relatedcardiogenic shock. Acta Chir Belg. 2001 Sep-Oct;101(5):226-31. PubMed PMID:11758106.

312: Olivotto I, Cecchi F, Casey SA, Dolara A, Traverse JH, Maron BJ. Impact ofatrial fibrillation on the clinical course of hypertrophic cardiomyopathy.Circulation. 2001 Nov 20;104(21):2517-24. PubMed PMID: 11714644.

313: Romero-Farina G, Candell-Riera J, Pereztol-Valdés O, Castell J, Aguadé S,Galve E, Palet J, Oller-Martínez G, Armadans L, Reina D, Soler-Soler J.[Myocardial perfusion SPECT and isotopic ventriculography in obstructive andnon-obstructive hypertrophic myocardiopathy]. Rev Esp Med Nucl. 2001Dec;20(7):530-6. Spanish. PubMed PMID: 11709138.

314: Protonotarios N, Tsatsopoulou A, Anastasakis A, Sevdalis E, McKoy G, StratosK, Gatzoulis K, Tentolouris K, Spiliopoulou C, Panagiotakos D, McKenna W,Toutouzas P. Genotype-phenotype assessment in autosomal recessive arrhythmogenic right ventricular cardiomyopathy (Naxos disease) caused by a deletion inplakoglobin. J Am Coll Cardiol. 2001 Nov 1;38(5):1477-84. PubMed PMID: 11691526.

315: Gueffet JP, Langlard JM, Burban M, Campion L, Bouhour JB. [Hypertrophiccardiomyopathy. Long-term clinical development in a regional cohort of 243patients]. Arch Mal Coeur Vaiss. 2001 Sep;94(9):967-74. French. PubMed PMID:11603071.

316: Doi Y, Kitaoka H. Hypertrophic cardiomyopathy in the elderly: significanceof atrial fibrillation. J Cardiol. 2001;37 Suppl 1:133-8. PubMed PMID: 11433817.

317: Kitaoka H, Hitomi N, Yabe T, Furuno T, Doi YL. Cardiovascular events andplasma atrial natriuretic peptide level in patients with hypertrophiccardiomyopathy. Am J Cardiol. 2001 Jun 1;87(11):1318-20. PubMed PMID: 11377368.

318: Mohiddin S, Fananapazir L. Advances in understanding hypertrophiccardiomyopathy. Hosp Pract (1995). 2001 May 15;36(5):23-5, 29-30, 33-6. PubMedPMID: 11361156.

319: Isomura T, Suma H, Horii T, Sato T, Kobashi T, Kanemitsu H, Hoshino J,Hisatomi K. Left ventricle restoration in patients with non-ischemic dilatedcardiomyopathy: risk factors and predictors of outcome and change of mid-termventricular function. Eur J Cardiothorac Surg. 2001 May;19(5):684-9. PubMed PMID:11343953.

320: Elliott PM, Gimeno Blanes JR, Mahon NG, Poloniecki JD, McKenna WJ. Relation between severity of left-ventricular hypertrophy and prognosis in patients withhypertrophic cardiomyopathy. Lancet. 2001 Feb 10;357(9254):420-4. PubMed PMID:11273061.

321: Havndrup O, Pettersson G, Kjeldsen K, Bundgaard H. Outcome of septalmyectomy in patients with hypertrophic obstructive cardiomyopathy. ScandCardiovasc J. 2000 Dec;34(6):564-9. PubMed PMID: 11214008.

322: Candell Riera J, Romero Farina G, Galve Basilio E, Palet Balart J, Armadans L, Dolores Reina M, García del Castillo H, Soler Soler J. [Value ofDoppler-echocardiography in the prognosis and follow up of hypertrophicmyocardiopathy]. Rev Esp Cardiol. 2001 Jan;54(1):7-15. Spanish. PubMed PMID:11141449.

323: Karibe A, Tobacman LS, Strand J, Butters C, Back N, Bachinski LL, Arai AE,Ortiz A, Roberts R, Homsher E, Fananapazir L. Hypertrophic cardiomyopathy caused by a novel alpha-tropomyosin mutation (V95A) is associated with mil cardiacphenotype, abnormal calcium binding to troponin, abnormal myosin cycling, andpoor prognosis. Circulation. 2001 Jan 2;103(1):65-71. PubMed PMID: 11136687.

324: Mohiddin SA, Begley D, Shih J, Fananapazir L. Myocardial bridging does notpredict sudden death in children with hypertrophic cardiomyopathy but isassociated with more severe cardiac disease. J Am Coll Cardiol. 2000Dec;36(7):2270-8. PubMed PMID: 11127472.

325: Spirito P, Rubartelli P. Alcohol septal ablation in the management ofobstructive hypertrophic cardiomyopathy. Ital Heart J. 2000 Nov;1(11):721-5.PubMed PMID: 11110513.

326: Holmgren D, Asplund EL, Berggren H, Bergh CH, Eriksson BO, Mårtensson G,Nilsson F. Thoracic organ transplantation in children. The Sahlgrenska UniversityHospital experience. Scand Cardiovasc J. 2000 Aug;34(4):426-32. PubMed PMID:10983679.

327: Slonim AE, Bulone L, Ritz S, Goldberg T, Chen A, Martiniuk F. Identificationof two subtypes of infantile acid maltase deficiency. J Pediatr. 2000Aug;137(2):283-5. PubMed PMID: 10931430.

328: Merrill WH, Friesinger GC, Graham TP Jr, Byrd BF 3rd, Drinkwater DC Jr,Christian KG, Bender HW Jr. Long-lasting improvement after septal myectomy forhypertrophic obstructive cardiomyopathy. Ann Thorac Surg. 2000 Jun;69(6):1732-5; discussion 1735-6. PubMed PMID: 10892916.

329: Spirito P, Bellone P, Harris KM, Bernabo P, Bruzzi P, Maron BJ. Magnitude ofleft ventricular hypertrophy and risk of sudden death in hypertrophiccardiomyopathy. N Engl J Med. 2000 Jun 15;342(24):1778-85. PubMed PMID: 10853000.

330: Helton E, Darragh R, Francis P, Fricker FJ, Jue K, Koch G, Mair D, Pierpont ME, Prochazka JV, Linn LS, Winter SC. Metabolic aspects of myocardial disease anda role for L-carnitine in the treatment of childhood cardiomyopathy. Pediatrics. 2000 Jun;105(6):1260-70. Erratum in: Pediatrics 2000 Sep;106(3):623. PubMed PMID:10835067.

331: Vester EG, Dees H, Dobran I, Hennersdorf M, Perings C, Heydthausen M, WinterJ, Strauer BE. [14-year experience with implantable cardioverter/defibrillators: determination of prognosis and discharge behavior]. Z Kardiol. 2000;89 Suppl3:194-205. German. PubMed PMID: 10810803.

332: Faber L, Meissner A, Ziemssen P, Seggewiss H. Percutaneous transluminalseptal myocardial ablation for hypertrophic obstructive cardiomyopathy: long termfollow up of the first series of 25 patients. Heart. 2000 Mar;83(3):326-31.PubMed PMID: 10677415; PubMed Central PMCID: PMC1729336.

333: Prykhod'ko VS, Riha OO. [Predictors of sudden death in children withcardiomyopathy]. Lik Sprava. 1999 Sep;(6):31-4. Ukrainian. PubMed PMID: 10626436.

334: Timmermans C, Rodriguez LM, Ayers GM, Siu A, Smeets J, Barenbrug P, Wellens HJ. Design and preliminary data of the Metrix Atrioverter expanded indicationtrial. J Interv Card Electrophysiol. 2000 Jan;4 Suppl 1:197-9. PubMed PMID:10590508.

335: Ostman-Smith I, Wettrell G, Riesenfeld T. A cohort study of childhoodhypertrophic cardiomyopathy: improved survival following high-dosebeta-adrenoceptor antagonist treatment. J Am Coll Cardiol. 1999 Nov15;34(6):1813-22. PubMed PMID: 10577575.

336: Schulte HD, Borisov K, Gams E, Gramsch-Zabel H, Lösse B, Schwartzkopff B.Management of symptomatic hypertrophic obstructive cardiomyopathy--long-termresults after surgical therapy. Thorac Cardiovasc Surg. 1999 Aug;47(4):213-8.PubMed PMID: 10522789.

337: Olivotto I, Maron BJ, Montereggi A, Mazzuoli F, Dolara A, Cecchi F.Prognostic value of systemic blood pressure response during exercise in acommunity-based patient population with hypertrophic cardiomyopathy. J Am CollCardiol. 1999 Jun;33(7):2044-51. PubMed PMID: 10362212.

338: Olearchyk AS. [Congenital heart diseases in adults]. Klin Khir.1998;(7):23-6. Ukrainian. PubMed PMID: 9989081.

339: Takagi E, Yamakado T, Nakano T. Prognosis of completely asymptomatic adultpatients with hypertrophic cardiomyopathy. J Am Coll Cardiol. 1999Jan;33(1):206-11. PubMed PMID: 9935031.

340: Yetman AT, Hamilton RM, Benson LN, McCrindle BW. Long-term outcome andprognostic determinants in children with hypertrophic cardiomyopathy. J Am CollCardiol. 1998 Dec;32(7):1943-50. PubMed PMID: 9857876.

341: Kyriakidis M, Triposkiadis F, Anastasakis A, Theopistou A, Tocta R,Barbetseas J, Gialafos J. Hypertrophic cardiomyopathy in Greece: clinical course and outcome. Chest. 1998 Oct;114(4):1091-6. PubMed PMID: 9792582.

342: Dimitrow PP, Czarnecka D, Kawecka-Jaszcz K, Dubiel JS. Progression ofasymmetric pattern of left ventricular hypertrophy in patients with hypertrophic cardiomyopathy accompanied by hypertension in the elderly. J Hum Hypertens. 1998 Sep;12(9):631-2. PubMed PMID: 9783493.

343: Englund A, Hnatkova K, Kulakowski P, Elliot PM, Malik M, McKenna WJ. Use of spectral turbulence analysis for the identification of patients at high risk for ventricular fibrillation and sudden death in patients with hypertrophiccardiomyopathy. Cardiology. 1998 Oct;90(2):79-82. PubMed PMID: 9778542.

344: Maki S, Ikeda H, Muro A, Yoshida N, Shibata A, Koga Y, Imaizumi T.Predictors of sudden cardiac death in hypertrophic cardiomyopathy. Am J Cardiol. 1998 Sep 15;82(6):774-8. PubMed PMID: 9761089.

345: Anan R, Shono H, Kisanuki A, Arima S, Nakao S, Tanaka H. Patients withfamilial hypertrophic cardiomyopathy caused by a Phe110Ile missense mutation inthe cardiac troponin T gene have variable cardiac morphologies and a favorableprognosis. Circulation. 1998 Aug 4;98(5):391-7. PubMed PMID: 9714088.

346: Cox GF, Souri M, Aoyama T, Rockenmacher S, Varvogli L, Rohr F, Hashimoto T, Korson MS. Reversal of severe hypertrophic cardiomyopathy and excellentneuropsychologic outcome in very-long-chain acyl-coenzyme A dehydrogenasedeficiency. J Pediatr. 1998 Aug;133(2):247-53. PubMed PMID: 9709714.

347: Ponoth P, Kerr A, Raudkivi PJ, Grahm K, Hydock D, Milson P. Surgicalcorrection of hypertrophic obstructive cardiomyopathy: seventeen-year Green Lane experience. J Card Surg. 1997 Sep-Oct;12(5):294-9. PubMed PMID: 9635266.

348: Cecchi F, Olivotto I, Montereggi A, Squillatini G, Dolara A, Maron BJ.Prognostic value of non-sustained ventricular tachycardia and the potential role of amiodarone treatment in hypertrophic cardiomyopathy: assessment in anunselected non-referral based patient population. Heart. 1998 Apr;79(4):331-6.PubMed PMID: 9616338; PubMed Central PMCID: PMC1728662.

349: Schönbeck MH, Brunner-La Rocca HP, Vogt PR, Lachat ML, Jenni R, Hess OM,Turina MI. Long-term follow-up in hypertrophic obstructive cardiomyopathy afterseptal myectomy. Ann Thorac Surg. 1998 May;65(5):1207-14. PubMed PMID: 9594839.

350: Charron P, Dubourg O, Desnos M, Isnard R, Hagege A, Bonne G, Carrier L,Tesson F, Bouhour JB, Buzzi JC, Feingold J, Schwartz K, Komajda M.Genotype-phenotype correlations in familial hypertrophic cardiomyopathy. Acomparison between mutations in the cardiac protein-C and the beta-myosin heavychain genes. Eur Heart J. 1998 Jan;19(1):139-45. PubMed PMID: 9503187.

351: Cecchi F, Olivotto I, Lazzeroni E, Chiriatti G, Sachero A, Beretta L,Giagnoni E, Renosto G, Montereggi A, Baldassarre S, Castelli G, Ciaccheri M.[Clinical course of hypertrophic cardiomyopathy in a non selected population. TheExperience of the Italian Multicenter Cardiomyopathy Study]. G Ital Cardiol. 1997Nov;27(11):1133-43. Italian. PubMed PMID: 9463057.

352: Seggewiss H, Gleichmann U, Faber L, Fassbender D, Schmidt HK, Strick S.Percutaneous transluminal septal myocardial ablation in hypertrophic obstructive cardiomyopathy: acute results and 3-month follow-up in 25 patients. J Am CollCardiol. 1998 Feb;31(2):252-8. PubMed PMID: 9462563.

353: Suda K, Kohl T, Kovalchin JP, Silverman NH. Echocardiographic predictors of poor outcome in infants with hypertrophic cardiomyopathy. Am J Cardiol. 1997 Sep 1;80(5):595-600. PubMed PMID: 9294988.

354: Rubin DN, Ballal RS, Marwick TH. Outcomes and cost implications of aclinical-based algorithm to guide the discriminate use of stress imaging beforenoncardiac surgery. Am Heart J. 1997 Jul;134(1):83-92. PubMed PMID: 9266787.

355: Moolman JC, Corfield VA, Posen B, Ngumbela K, Seidman C, Brink PA, WatkinsH. Sudden death due to troponin T mutations. J Am Coll Cardiol. 1997 Mar1;29(3):549-55. PubMed PMID: 9060892.

356: Albanesi Filho FM, Castier MB, Diamant JD, Lopes JS, Lopes AS, Ginefra P.[Apical hypertrophic cardiomyopathy with right ventricular involvement]. Arq BrasCardiol. 1997 Feb;68(2):119-24. Portuguese. PubMed PMID: 9433839.

357: Nunoda S, Kurosawa R, Kogashi K, Yamagishi S, Mitsui F, Funabashi W. Points to note from indications for heart transplantation to post-heart transplant care:from the care of patients with refractory heart failure and overseas hearttransplantation. Heart Vessels. 1997;Suppl 12:37-40. PubMed PMID: 9476540.

358: Theodoro DA, Danielson GK, Feldt RH, Anderson BJ. Hypertrophic obstructivecardiomyopathy in pediatric patients: results of surgical treatment. J ThoracCardiovasc Surg. 1996 Dec;112(6):1589-97; discussion 1597-9. PubMed PMID:8975851.

359: Yanaga K, Takenaka K, Yamamoto K, Nishizaki T, Shirabe K, Shimada M,Kawahara N, Chishaki A, Sugimachi K. Cardiac complications after hepaticresection. Br J Surg. 1996 Oct;83(10):1448-51. PubMed PMID: 8944469.

360: McCully RB, Nishimura RA, Tajik AJ, Schaff HV, Danielson GK. Extent ofclinical improvement after surgical treatment of hypertrophic obstructivecardiomyopathy. Circulation. 1996 Aug 1;94(3):467-71. PubMed PMID: 8759090.

361: Jault F, Gandjbakhch I, Rama A, Nataf P, Dorent R, Bors V, Pavie A, CabrolC. [Long term results of the surgical treatment of obstructive hypertrophiccardiomyopathies]. Arch Mal Coeur Vaiss. 1996 Jun;89(6):679-84. French. PubMedPMID: 8760652.

362: Ko YL, Chen JJ, Tang TK, Cheng JJ, Lin SY, Liou YC, Kuan P, Wu CW, Lien WP, Liew CC. Malignant familial hypertrophic cardiomyopathy in a family with a453Arg-->Cys mutation in the beta-myosin heavy chain gene: coxistence of sudden death and end-stage heart failure. Hum Genet. 1996 May;97(5):585-90. PubMed PMID:8655135.

363: Robbins RC, Stinson EB. Long-term results of left ventricular myotomy andmyectomy for obstructive hypertrophic cardiomyopathy. J Thorac Cardiovasc Surg.1996 Mar;111(3):586-94. PubMed PMID: 8601973.

364: Nishimura T, Nagata S, Uehara T, Morozumi T, Ishida Y, Nakata T, Iimura O,Kurata C, Wakabayashi Y, Sugihara H, Otsuki K, Wada T, Koga Y. Prognosis ofhypertrophic cardiomyopathy: assessment by 123I-BMIPP(beta-methyl-p-(123I)iodophenyl pentadecanoic acid) myocardial single photonemission computed tomography. Ann Nucl Med. 1996 Feb;10(1):71-8. PubMed PMID:8814730.

365: Iacovino JR. The non mortality of hypertrophic cardiomyopathy in anunselected, community diagnosed and treated population. J Insur Med.

1996;28(1):51-4. PubMed PMID: 10163622.

366: Ten Cate FJ. Prognosis of hypertrophic cardiomyopathy. J Insur Med.1996;28(1):42-5. PubMed PMID: 10163618.

367: Cecchi F, Olivotto I, Montereggi A, Santoro G, Dolara A, Maron BJ.Hypertrophic cardiomyopathy in Tuscany: clinical course and outcome in anunselected regional population. J Am Coll Cardiol. 1995 Nov 15;26(6):1529-36.PubMed PMID: 7594081.

368: Yoshida M, Nakamura Y, Fukuhara T, Higashikawa M, Okada M, Shichiri G,Kinoshita M. Prognostic determinants of hypertrophic cardiomyopathy--the results of the Shiga Cardiomyopathy Study. Jpn Circ J. 1995 Nov;59(11):745-53. PubMedPMID: 8747764.

369: Seiler C, Jenni R, Vassalli G, Turina M, Hess OM. Left ventricular chamberdilatation in hypertrophic cardiomyopathy: related variables and prognosis inpatients with medical and surgical therapy. Br Heart J. 1995 Nov;74(5):508-16.PubMed PMID: 8562235; PubMed Central PMCID: PMC484070.

370: Heric B, Lytle BW, Miller DP, Rosenkranz ER, Lever HM, Cosgrove DM. Surgicalmanagement of hypertrophic obstructive ardiomyopathy. Early and late results. J Thorac Cardiovasc Surg. 1995 Jul;110(1):195-206; discussion 206-8. PubMed PMID:7609544.

371: Azzano O, Bozio A, Sassolas F, Di Filippo S, Agé C, André M,Jocteur-Monrozier D, Normand J. [Natural history of hypertrophic obstructivecardiomyopathy in young patients: apropos of 40 cases]. Arch Mal Coeur Vaiss.1995 May;88(5):667-72. French. PubMed PMID: 646275.

372: Millaire A. [Surgical treatment of hypertrophic cardiomyopathy; techniques, indications and results]. Arch Mal Coeur Vaiss. 1995 Apr;88(4 Suppl):585-8.French. PubMed PMID: 7487304.

373: Ishikawa K, Hashimoto H, Mitani S, Toki Y, Okumura K, Ito T. Enalaprilimproves heart failure induced by monocrotaline without reducing pulmonaryhypertension in rats: roles of preserved myocardial creatine kinase and lactatedehydrogenase isoenzymes. Int J Cardiol. 1995 Jan 6;47(3):225-33. PubMed PMID:7721499.

374: Spirito P, Rapezzi C, Autore C, Bruzzi P, Bellone P, Ortolani P, Fragola PV,Chiarella F, Zoni-Berisso M, Branzi A, et al. Prognosis of asymptomatic patients with hypertrophic cardiomyopathy and nonsustained ventricular tachycardia.Circulation. 1994 Dec;90(6):2743-7. PubMed PMID: 7994816.

375: Lefroy DC. Cardiac arrest and hypertrophic cardiomyopathy. Role of theimplantable defibrillator. BMJ. 1994 Nov 12;309(6964):1277-9. PubMed PMID:7888852; PubMed Central PMCID: PMC2541794.

376: Turrentine MW, Kesler KA, Caldwell R, Darragh R, Means L, Mahomed Y, BrownJW. Cardiac transplantation in infants and children. Ann Thorac Surg. 1994Mar;57(3):546-53; discussion 554. PubMed PMID: 8147620.

377: Anan R, Greve G, Thierfelder L, Watkins H, McKenna WJ, Solomon S, Vecchio C,Shono H, Nakao S, Tanaka H, et al. Prognostic implications of novel beta cardiac myosin heavy chain gene mutations that cause familial hypertrophiccardiomyopathy. J Clin Invest. 1994 Jan;93(1):280-5. PubMed PMID: 8282798; PubMedCentral PMCID: PMC293763.

378: Fananapazir L, Epstein ND. Genotype-phenotype correlations in hypertrophiccardiomyopathy. Insights provided by comparisons of kindreds with distinct andidentical beta-myosin heavy chain gene mutations. Circulation. 1994Jan;89(1):22-32. PubMed PMID: 8281650.

379: Schoendube FA, Klues HG, Reith S, Messmer BJ. Surgical correction ofhypertrophic obstructive cardiomyopathy with combined myectomy, mobilisation and partial excision of the papillary muscles. Eur J Cardiothorac Surg.1994;8(11):603-8. PubMed PMID: 7893501.

380: Stone CD, McIntosh CL, Hennein HA, Maron BJ, Clark RE. Operative treatmentof pediatric obstructive hypertrophic cardiomyopathy: a 26-year experience. AnnThorac Surg. 1993 Dec;56(6):1308-13; discussion 1313-4. PubMed PMID: 8267429.

381: Ferreira P, Fonseca C, Morais H, Carvalho A, Ceia F, Luís AS. ["Severe"congestive heart failure at a medical center]. Rev Port Cardiol. 1993Nov;12(11):919-30, 900. Portuguese. PubMed PMID: 8305244.

382: Kofflard MJ, Waldstein DJ, Vos J, ten Cate FJ. Prognosis in hypertrophiccardiomyopathy observed in a large clinic population. Am J Cardiol. 1993 Oct15;72(12):939-43. PubMed PMID: 8213552.

383: Schulte HD, Bircks WH, Loesse B, Godehardt EA, Schwartzkopff B. Prognosis ofpatients with hypertrophic obstructive cardiomyopathy after transaortic myectomy.Late results up to twenty-five years. J Thorac Cardiovasc Surg. 1993Oct;106(4):709-17. PubMed PMID: 8412267.

384: Delahaye F, Jegaden O, de Gevigney G, Genoud JL, Perinetti M, Montagna P,Delaye J, Mikaeloff P. Postoperative and long-term prognosis ofmyotomy-myomectomy for obstructive hypertrophic cardiomyopathy: influence ofassociated mitral valve replacement. Eur Heart J. 1993 Sep;14(9):1229-37. PubMed PMID: 8223738.

385: de Vivie ER, Borowski A, Mehlhorn U. Reduction of the left-ventricularoutflow-tract obstruction by aortoventriculoplasty--long-term results of 96patients. Thorac Cardiovasc Surg. 1993 Aug;41(4):216-23. PubMed PMID: 8211925.

386: Hina K, Kusachi S, Iwasaki K, Nogami K, Moritani H, Kita T, Taniguchi G,Tsuji T. Progression of left ventricular enlargement in patients withhypertrophic cardiomyopathy: incidence and prognostic value. Clin Cardiol. 1993May;16(5):403-7. PubMed PMID: 8504574.

387: Chikamori T, Doi YL, Akizawa M, Yonezawa Y, Ozawa T, McKenna WJ. Comparison of clinical, morphological, and prognostic features in hypertrophiccardiomyopathy between Japanese and western patients. Clin Cardiol. 1992Nov;15(11):833-7. PubMed PMID: 10969627.

388: Fananapazir L, Chang AC, Epstein SE, McAreavey D. Prognostic determinants inhypertrophic cardiomyopathy. Prospective evaluation of a therapeutic strategybased on clinical, Holter, hemodynamic, and electrophysiological findings.Circulation. 1992 Sep;86(3):730-40. PubMed PMID: 1516184.

389: Lazzeroni E, Rolli A, Aurier E, Botti G. Clinical significance of coronaryartery disease in hypertrophic cardiomyopathy. Am J Cardiol. 1992 Aug15;70(4):499-501. PubMed PMID: 1642188.

390: Atkins CE, Gallo AM, Kurzman ID, Cowen P. Risk factors, clinical signs, and survival in cats with a clinical diagnosis of idiopathic hypertrophiccardiomyopathy: 74 cases (1985-1989). J Am Vet Med Assoc. 1992 Aug15;201(4):613-8. PubMed PMID: 1517140.

391: Watkins H, Rosenzweig A, Hwang DS, Levi T, McKenna W, Seidman CE, SeidmanJG. Characteristics and prognostic implications of myosin missense mutations infamilial hypertrophic cardiomyopathy. N Engl J Med. 1992 Apr 23;326(17):1108-14. PubMed PMID: 1552912.

392: Shannon JA, Hammill SC, Gersh BJ. Predictive value of earlyelectrophysiologic testing in determining long-term outcome with amiodarone

treatment in patients with sustained ventricular tachycardia. Mayo Clin Proc.1991 Nov;66(11):1114-9. PubMed PMID: 1943242.

393: Garson A Jr. Sudden death in the young. Hosp Pract (Off Ed). 1991 Jun15;26(6):51-60. PubMed PMID: 1760004.

394: Aron LA, Hertzeanu HL, Fisman EZ, Nosrati IS, Kellermann JJ. Prognosis ofnonobstructive hypertrophic cardiomyopathy. Am J Cardiol. 1991 Jan15;67(2):215-7. PubMed PMID: 1987728.

395: Toshima H, Koga Y, Wada T. [The prognosis of cardiomyopathy]. Nihon Rinsho. 1991 Jan;49(1):155-60. Japanese. PubMed PMID: 2002595.

396: Drăgulescu SI, Brînzan O, Streian C. Prevalence of arrhythmias duringexercise stress testing in patients with hypertrophic cardiomyopathy. Rom JIntern Med. 1991 Jan-Jun;29(1-2):33-7. PubMed PMID: 1947710.

397: Pelliccia F, Cianfrocca C, Romeo F, Reale A. Natural history of hypertrophiccardiomyopathy in the elderly. Cardiology. 1991;78(4):329-33. PubMed PMID:1889051.

398: Pelliccia F, Cianfrocca C, Romeo F, Reale A. Hypertrophic cardiomyopathy:long-term effects of propranolol versus verapamil in preventing sudden death in"low-risk" patients. Cardiovasc Drugs Ther. 1990 Dec;4(6):1515-8. PubMed PMID:2081144.

399: Fay WP, Taliercio CP, Ilstrup DM, Tajik AJ, Gersh BJ. Natural history ofhypertrophic cardiomyopathy in the elderly. J Am Coll Cardiol. 1990Oct;16(4):821-6. PubMed PMID: 2212364.

400: Pelliccia F, Cianfrocca C, Cristofani R, Romeo F, Reale A.Electrocardiographic findings in patients with hypertrophic cardiomyopathy. Relation to presenting features and prognosis. J Electrocardiol. 1990Jul;23(3):213-22. PubMed PMID: 2384727.401: Alfonso F, Nihoyannopoulos P, Stewart J, Dickie S, Lemery R, McKenna WJ.Clinical significance of giant negative T waves in hypertrophic cardiomyopathy. JAm Coll Cardiol. 1990 Apr;15(5):965-71. PubMed PMID: 2312983.

402: Chikamori T, Dickie S, Poloniecki JD, Myers MJ, Lavender JP, McKenna WJ.Prognostic significance of radionuclide-assessed diastolic function inhypertrophic cardiomyopathy. Am J Cardiol. 1990 Feb 15;65(7):478-82. PubMed PMID:2305687.

403: Romeo F, Cianfrocca C, Pelliccia F, Colloridi V, Cristofani R, Reale A.Long-term prognosis in children with hypertrophic cardiomyopathy: an analysis of 37 patients aged less than or equal to 14 years at diagnosis. Clin Cardiol. 1990 Feb;13(2):101-7. PubMed PMID: 2306882.

404: Parfrey PS, Griffiths SM, Harnett JD, Taylor R, King A, Hand J, Barre PE.Outcome of congestive heart failure, dilated cardiomyopathy, hypertrophichyperkinetic disease, and ischemic heart disease in dialysis patients. Am JNephrol. 1990;10(3):213-21. PubMed PMID: 2143353.

405: Koga Y, Ogata M, Kihara K, Tsubaki K, Toshima H. Sudden death inhypertrophic and dilated cardiomyopathy. Jpn Circ J. 1989 Dec;53(12):1546-56.PubMed PMID: 2632825.

406: Seiler C, Hess OM, Turina J, Schoenbeck M, Jenni R, Rothlin M, Senning A,Turina M, Krayenbühl HP. [Long-term course of hypertrophic cardiomyopathy: drugversus surgical therapy]. Schweiz Med Wochenschr. 1989 Oct 28;119(43):1511-4.German. PubMed PMID: 2609126.

407: Kurita A, Uehata A, Nishioka T, Takase B, Maruyama T, Nakamura H, NakagawaA, Shiga M, Asano G. [An investigation of sudden cardiac death in apparentlyhealthy young men by annual health examination]. Nihon Eiseigaku Zasshi. 1989Aug;44(3):739-47. PubMed PMID: 2810875.

408: Lin CK, Kuo PL, Liu HC, Yau KI, Chang HS, Wang TR, Chen SH. Clinicalanalysis of infants of diabetic mothers. Zhonghua Min Guo Xiao Er Ke Yi Xue HuiZa Zhi. 1989 Jul-Aug;30(4):233-9. PubMed PMID: 2637603.

409: Herrmann G, Haverich A, Cremer J, Fieguth HG, Jurmann M, Wahlers T, BorstHG. [Indications and results of orthotopic heart transplantation in coronaryheart disease]. Z Gesamte Inn Med. 1989 Jan 1;44(1):6-11. German. PubMed PMID:2652897.

410: Yabe T, Doi Y, Yonesawa Y, Takada J, Yamada M, Odawara H, Ozawa T.[Assessment of prognosis of patients with ventricular tachycardia: role of theunderlying heart disease]. J Cardiol Suppl. 1989;22:123-5. Japanese. PubMed PMID:2640854.

411: Emoto R, Yokota Y, Miki T, Nomura H, Miki T, Usuki S, Chou HT, Kurozumi H,Seo T, Fukuzaki H. [Prognosis of hypertrophic cardiomyopathy: echocardiographicand postmortem histopathologic study of 30 patients]. J Cardiol. 1988Sep;18(3):695-703. Japanese. PubMed PMID: 3249285.

412: McKenna WJ. Noninvasive assessment and management of the patient at highrisk of sudden cardiac death. Clin Cardiol. 1988 Mar;11(3 Suppl 2):II22-5. PubMedPMID: 3271191.

413: Delahaye F, Perinetti M, Chambre G, Coll-Mazzei J, Jegaden O, Didier B,Delaye J, Mikaeloff P. [Results of myomectomy in obstructive cardiomyopathy.Apropos of 28 cases]. Arch Mal Coeur Vaiss. 1988 Feb;81(2):177-84. French. PubMedPMID: 3130817.

414: McKenna WJ, Franklin RC, Nihoyannopoulos P, Robinson KC, Deanfield JE.Arrhythmia and prognosis in infants, children and adolescents with hypertrophiccardiomyopathy. J Am Coll Cardiol. 1988 Jan;11(1):147-53. PubMed PMID: 3335690.

415: Cserhalmi L, Szabóky F, Ilovszky Z, Csukás A, Huszár G. Noninvasivefollow-up study in hypertrophic obstructive cardiomyopathy. Acta Cardiol.1988;43(3):323-7. PubMed PMID: 3261089.

416: Samukawa M, Hasegawa K, Harada Y, Nakao M, Tadaoka S, Yoneda M, Fujiwara T, Nakamura T, Nezuo S, Sawayama T. [Clinical features and significance ofhypertrophic cardiomyopathy with atrial fibrillation]. J Cardiol. 1987Sep;17(3):465-74. Japanese. PubMed PMID: 3453843.

417: Conte A, Hess OM, Maire R, Gautschi K, Brogli S, Knaus U, Krayenbühl HP.[Clinical significance of serum carnitine in the course and prognosis of dilated cardiomyopathy]. Z Kardiol. 1987 Jan;76(1):15-24. German. PubMed PMID: 3564613.

418: Yokota Y, Seo T, Fukuzaki H. [Cardiac function and prognosis in hypertrophiccardiomyopathy]. J Cardiol Suppl. 1987;16:79-88. Japanese. PubMed PMID: 3509684.

419: Schulte HD, Bircks W, Lösse B. Techniques and complications of transaorticsubvalvular myectomy in patients with hypertrophic obstructive cardiomyopathy(HOCM). Z Kardiol. 1987;76 Suppl 3:145-51. PubMed PMID: 3433868.

420: Hopf R, Kaltenbach M. 10-year results and survival of patients withypertrophic cardiomyopathy treated with calcium antagonists. Z Kardiol. 1987;76 Suppl 3:137-44. PubMed PMID: 3433866.

421: Brandt CM, Zaehringer M, Bas G, Desroches P, Drui S, Fincker JL.[Hypertrophic obstructive cardiomyopathy in elderly patients. Retrospective studyof 23 cases]. Arch Mal Coeur Vaiss. 1986 Dec;79(13):1925-31. French. PubMed PMID:3105505.

422: Bircks W. Surgical treatment of hypertrophic cardiomyopathy (Düsseldorfxperience). Postgrad Med J. 1986 Jun;62(728):571-4. PubMed PMID: 3774695; PubMedCentral PMCID: PMC2418767.

423: McKenna WJ, Oakley CM, Krikler DM, Goodwin JF. Improved survival withmiodarone in patients with hypertrophic cardiomyopathy and ventriculartachycardia. Br Heart J. 1985 Apr;53(4):412-6. PubMed PMID: 4039188; PubMedCentral PMCID: PMC481782.

424: Toshima H. Research on cardiomyopathy in Japan. Heart Vessels Suppl.1985;1:14-7. PubMed PMID: 3843577.

425: McKenna WJ, Deanfield JE. Hypertrophic cardiomyopathy: an important cause ofsudden death. Arch Dis Child. 1984 Oct;59(10):971-5. ubMed PMID: 6541894; PubMedCentral PMCID: PMC1628861.

426: Koga Y, Itaya K, Toshima H. Prognosis in hypertrophic cardiomyopathy. AmHeart J. 1984 Aug;108(2):351-9. PubMed PMID: 6540514.

427: Fighali S, Krajcer Z, Leachman RD. Septal myomectomy and mitral valvereplacement for idiopathic hypertrophic subaortic stenosis: short-and long-term follow-up. J Am Coll Cardiol. 1984 May;3(5):1127-34. PubMed PMID: 6538585.

428: Lassabe G, Kieny JR, Dumeny P, Grison D, Sacrez A. [Course and prognosis of primary hypertrophic cardiomyopathies]. Sem Hop. 1983 Dec 1;59(44):3047-51.French. PubMed PMID: 6320392.

429: McKenna WJ. Arrhythmia and prognosis in hypertrophic cardiomyopathy. EurHeart J. 1983 Nov;4 Suppl F:225-34. PubMed PMID: 6686539.

430: Rothlin ME, Gobet D, Haberer T, Krayenbuehl HP, Turina M, Senning A.Surgical treatment versus medical treatment in hypertrophic obstructivecardiomyopathy. Eur Heart J. 1983 Nov;4 Suppl F:215-23. PubMed PMID: 6686538.

431: Maron BJ, Epstein SE, Morrow AG. Symptomatic status and prognosis ofpatients after operation for hypertrophic obstructive cardiomyopathy: efficacy ofventricular septal myotomy and myectomy. Eur Heart J. 1983 Nov;4 Suppl F:175-85. PubMed PMID: 6686533.

432: Loogen F, Kuhn H, Gietzen F, Lösse B, Schulte HD, Bircks W. Clinical course and prognosis of patients with typical and atypical hypertrophic obstructive and with hypertrophic non-obstructive cardiomyopathy. Eur Heart J. 1983 Nov;4 SupplF:145-53. PubMed PMID: 6686530.

433: Bauman PM, Fergusson DJ. Dilated cardiomyopathy in Hawaii. A retrospectivestudy of 13 patients. Hawaii Med J. 1983 Mar;42(3):60, 62, 64. PubMed PMID:6683269.

434: Kuhn H, Gietzen F, Mercier J, Lösse B, Köhler E, Schulte HD, Bircks W,Loogen F. [Clinical aspects, course and prognosis of various forms ofhypertrophic cardiomyopathy]. Z Kardiol. 1983 Feb;72(2):83-98. German. PubMedPMID: 6221476.

435: Lassabe G, Kieny JR, Dumeny P, Grison D, Sacrez A. [Course and prognosis of primary hypertrophic cardiomyopathies]. Ann Cardiol Angeiol (Paris). 1983Jan-Feb;32(1):27-31. French. PubMed PMID: 6683478.

436: McKenna WJ, Borggrefe M, England D, Deanfield J, Oakley CM, Goodwin JF. The natural history of left ventricular hypertrophy in hypertrophic cardiomyopathy:an electrocardiographic study. Circulation. 1982 Dec;66(6):1233-40. PubMed PMID: 6128085.

437: Jones M, Barnhart GR, Morrow AG. Late results after operations for leftventricular outflow tract obstruction. Am J Cardiol. 1982 Sep;50(3):569-79.PubMed PMID: 6214178.

438: Maron BJ, Savage DD, Wolfson JK, Epstein SE. Prognostic significance of 24hour ambulatory electrocardiographic monitoring in patients with hypertrophiccardiomyopathy: a prospective study. Am J Cardiol. 1981 Aug;48(2):252-7. PubMedPMID: 7196685.

439: Baandrup U, Florio RA, Roters F, Olsen EG. Electron microscopicinvestigation of endomyocardial biopsy samples in hypertrophy and cardiomyopathy.A semiquantitative study in 48 patients. Circulation. 1981 Jun;63(6):1289-98.PubMed PMID: 6452971.

440: McKenna W, Deanfield J, Faruqui A, England D, Oakley C, Goodwin J. Prognosisin hypertrophic cardiomyopathy: role of age and clinical, electrocardiographicand hemodynamic features. Am J Cardiol. 1981 Mar;47(3):532-8. PubMed PMID:7193406.

441: Doi YL, McKenna WJ, Chetty S, Oakley CM, Goodwin JF. Prediction of mortalityand serious ventricular arrhythmia in hypertrophic cardiomyopathy. Anechocardiographic study. Br Heart J. 1980 Aug;44(2):150-7. PubMed PMID: 7191710; PubMed Central PMCID: C482374.

442: Chizner MA, Pearle DL, deLeon AC Jr. The natural history of aortic stenosis in adults. Am Heart J. 1980 Apr;99(4):419-24. PubMed PMID: 7189084.

443: Shirey EK, Proudfit WL, Hawk WA. Primary myocardial disease. Correlationwith clinical findings, angiographic and biopsy diagnosis. Follow-up of 139patients. Am Heart J. 1980 Feb;99(2):198-207. PubMed PMID: 7188716.

444: Orinius E. Prognosis in hypertrophic obstructive cardiomyopathy. Acta MedScand. 1979;206(4):289-92. PubMed PMID: 159601.

445: Ceci V, Milazzotto F, Masini V. [A study of the natural evolution ofypertrophic obstructive cardiomyopathy. Analysis of 100 cases (authors'

transl)]. G Ital Cardiol. 1978;8(11):1154-60. Italian. PubMed PMID: 572792.

446: Ceci V, Malinconico U, Pulignano M, Milazzotto F, Masini V. [The course ofypertrophic obstructive cardiomyopathy. A preliminary study of 40 cases(author's transl)]. G Ital Cardiol. 1978;8(1):111-6. Italian. PubMed PMID:564801.

447: Emeriau JP, Besse P, Sicart M, Conri C, Martin P, Bricaud H. [Alternationsof cardiac performance in the avoluations of obstructive cardiomyopathy.Prognostic significance (author's transl)]. Ann Radiol (Paris). 1977Apr-May;20(3-4):371-8. French. PubMed PMID: 560159.

448: Jaumin P, Cosyns J, Kestens-Servaye Y, Kremer R, Goenen M, Ponlot R,Schoevaerdts JC, Chalant CH. Idiopathic hypertrophic subaortic stenosis:long-term surgical results. J Cardiovasc Surg (Torino). 1976 Nov-Dec;17(6):541-7.PubMed PMID: 1033183.

449: Hanania G, Marino JP, Magnier S, Baragan J, Guillemaut P, Fernandez F,Gerbaux A. [Spontaneous development of obstructive myocardiopathies]. Arch MalCoeur Vaiss. 1976 Oct;69(10):1023-32. French. PubMed PMID: 827995.

450: Gerbaux A, Hanania G, Godefroid A, Baragan J, Maouad J, Gay J. [Long termresults of surgical treatment of obstructive myocardiopathy by intervention onthe interventricular septum]. Arch Mal Coeur Vaiss. 1976 Aug;69(8):791-800.French. PubMed PMID: 823918.

451: Bouhour JB, Petitier H, De Lajartre AY, Almazor M, Nicolas G, Horeau J.[Myocardial biopsy in congestive myocardiopathies of apparently primary origin]. Arch Mal Coeur Vaiss. 1976 May;69(5):485-94. French. PubMed PMID: 821418.

452: Maron BJ, Henry WL, Clark CE, Redwood DR, Roberts WC, Epstein SE. Asymetric septal hypertrophy in childhood. Circulation. 1976 Jan;53(1):9-19. PubMed PMID:942659.

453: Bigelow WG, Trimble AS, Wigle ED, Adelman AG, Felderhof CH. The treatment ofmuscular subaortic stenosis. J Thorac Cardiovasc Surg. 1974 Sep;68(3):384-92.PubMed PMID: 4369369.

454: Levy MJ, Vidne B, Wurtzel M, Aygen M. Open heart surgery for congenitalacyanotic cardiac malformations. Harefuah. 1974 May 1;86(9):452-4. Hebrew. PubMedPMID: 4858079.

455: Hardarson T, De la Calzada CS, Curiel R, Goodwin JF. Prognosis and mortalityof hypertrophic obstructive cardiomyopathy. Lancet. 1973 Dec 29;2(7844):1462-7.PubMed PMID: 4129311.

456: Apitz J, Schröter HJ, Schmaltz AA, Gaissmaier U. [Stenoses and atresias ofthe aortic valve and the thoracic aorta in infants]. Thoraxchir Vask Chir. 1971Oct;19(5):402-7. German. PubMed PMID: 5286837.457: Brunner L, Kirchhoff PG, Heidbreder D, Heisig B, Hoffmeister HE, Kaese HJ,Rastan H, Regensburger D, Stapenhorst K, Konez J. [Results of restorative surgeryin the left efficient tract and valve replacement in children and adolescents].Thoraxchir Vask Chir. 1971 Aug;19(4):317-32. German. PubMed PMID: 5284966.

458: Bentall HH. The place of surgery in hypertrophic obstructive cardiomyopathy (idiopathic hypertrophic subaortic stenosis). J Thorac Cardiovasc Surg. 1966Jan;51(1):49-52. PubMed PMID: 5948055.

459: BENTALL HH, CLELAND WP, OAKLEY CM, SHAH PM, STEINER RE, GOODWIN JF. SURGICALTREATMENT AND POST-OPERATIVE HAEMODYNAMIC STUDIES IN HYPERTROPHIC OBSTRUCTIVECARDIOMYOPATHY. Br Heart J. 1965 Jul;27:585-94. PubMed PMID: 14324118; PubMedCentral PMCID: PMC503349.

Embase

SEARCH QUERY

'hypertrophic cardiomyopathy'' and prognosis not ('interventional cardiovascular procedure'/exp or 'interventional cardiovascular procedure') or ('hypertrophic cardiomyopathy':ab,ti and survival:ab,ti) or ('hypertrophic cardiomyopathy' and mortality) or ('hypertrophic cardiomyopathy' and survival and clinical and outcome) and [2017-2017]/py

----

1. The role of SIRT1 in diabetic cardiomyopathy

2. Updates in heart failure: Highlights from the Iranian Joint Cardiovascular Congress Tehran, Iran, 1–4 March 2016

3. Curcumin as a potential protective compound against cardiac diseases

4. Delayed Myocardial Enhancement in Pediatric Hypertrophic Cardiomyopathy: Correlation with LV Function, Echocardiography, and Demographic Parameters

5. Comparison of Frequency of Ischemic Cardiovascular Events in Patients With Aortic Stenosis With Versus Without Asymmetric Septal Hypertrophy (from the SEAS Trial)

6. Management of Heart Failure in Cancer Patients and Cancer Survivors

7. Evaluation of right ventricular function using liver stiffness in patients with left ventricular assist device

8. Predicting risk of sudden death in hypertrophic cardiomyopathy: Can additional simple markers help?

9. Hypertrophic cardiomyopathy: The edge-to-edge secures the correction of the systolic anterior motion

10. Uncomplicated Pregnancy in a Patient Treated With Alcohol Septal Ablation for Hypertrophic Obstructive Cardiomyopathy

11. Exome sequencing identifies primary carnitine deficiency in a family with cardiomyopathy and sudden death

12. Efficacy and safety of alcohol septal ablation in patients over 65 years old with obstructive hypertrophic cardiomyopathy

13. The clinical features, outcomes and genetic characteristics of hypertrophic cardiomyopathy patients with severe right ventricular hypertrophy

14. Breaking the rules—Higher urgency listings by exception: Challenges when a child's life is at stake

15. Urgent listing exceptions and outcomes in pediatric heart transplantation: Comparison to standard criteria patients

16. The preparticipation physical evaluation

17. Life-long tailoring of management for patients with hypertrophic cardiomyopathy: Awareness and decision-making in changing scenarios

18. A Test in Context: Myocardial Strain Measured by Speckle-Tracking Echocardiography

19. Hypertrophic Cardiomyopathy With Left Ventricular Apical Aneurysm: Implications for Risk Stratification and Management

20. Saving More Lives

21. Effect of cellular and extracellular pathology assessed by T1 mapping on regional contractile function in hypertrophic cardiomyopathy

22. Relationship between aetiology and left ventricular systolic dysfunction in hypertrophic cardiomyopathy

23. Heartbeat: Intervention for asymptomatic severe aortic stenosis?

24. Comparison of Maximal Wall Thickness in Hypertrophic Cardiomyopathy Differs Between Magnetic Resonance Imaging and Transthoracic Echocardiography

25. 77-year-old female with syncope

26. Survival After Heart Transplantation in Patients With Arrhythmogenic Right Ventricular Cardiomyopathy

27. Left atrial size and function in hypertrophic cardiomyopathy patients and risk of new-onset atrial fibrillation

28. Combined heart and kidney transplantation in Fabry's disease: Long-term outcomes in two patients

29. Amiodarone use in patients listed for heart transplant is associated with increased 1-year post-transplant mortality

30. A Novel Founder Mutation in MYBPC3: Phenotypic Comparison With the Most Prevalent MYBPC3 Mutation in Spain

31. Psen1 as an adjunct for diagnosis of human myocarditis

32. Successful MACE risk stratification in hypertrophic cardiomyopathy patients using different 2D speckle-tracking TTE approaches

33. Echocardiographic comparison between left ventricular non-compaction and hypertrophic cardiomyopathy

34. Usefulness of Genetic Testing in Hypertrophic Cardiomyopathy: an Analysis Using Real-World Data

35. Diagnosis and Management of Endomyocardial Fibrosis

36. Stress echo 2020: The international stress echo study in ischemic and non-ischemic heart disease

37. The Transgenic Diabetic Pig Heart: A “Sweet Heart” for Translational Cardiovascular Research

38. Device-related infective endocarditis in cardiac resynchronization therapy recipients — Single center registry with over 2500 person-years follow up

39. Lung adenocarcinoma expressing receptor for advanced glycation end-products with primary systemic AL amyloidosis: A case report and literature review

40. Long-term survival after acute myocardial infarction in patients with hypertrophic cardiomyopathy

41. Outcomes in hypertrophic cardiomyopathy patients with and without atrial fibrillation: A survival meta-analysis

42. Incidence of Device-Detected Atrial Fibrillation and Long-Term Outcomes in Patients With Hypertrophic Cardiomyopathy

43. Pediatric heart transplantation: Past, present and future

44. Prognostic significance of non-dilated left ventricular size and mitral regurgitation in patients with dilated phase of hypertrophic cardiomyopathy

45. Cardiomyopathy and ion channel diseases registry: The Szeged cardiogen registry

46. Higher copeptin levels are associated with worse outcome in patients with hypertrophic cardiomyopathy

47. Comparison of Long-Term Outcome between Apical and Asymmetric Septal Hypertrophic Cardiomyopathy

48. Syncope in primary prevention implantable cardioverter defibrillator implantation

49. Genetic determinants of myocardial dysfunction

50. Hypertrophic cardiomyopathy

51. The good and bad of exercise

52. Pre-participation cardiovascular evaluation for athletic participants to prevent sudden death: Position paper from the EHRA and the EACPR, branches of the ESC. Endorsed by APHRS, HRS, and SOLAECE

53. Pre-participation cardiovascular evaluation for athletic participants to prevent sudden death: Position paper from the EHRA and the EACPR, branches of the ESC. Endorsed by APHRS, HRS, and SOLAECE

54. Relevance of mouse models of cardiac fibrosis and hypertrophy in cardiac research

55. Clinical cardiac regenerative studies in children

56. Coronary microvascular dysfunction: Epidemiology, pathogenesis, prognosis, diagnosis, risk factors and therapy

57. Current perspectives in coronary microvascular dysfunction

58. Left ventricular hypertrophy or storage disease? the incremental value of speckle tracking strain bull's-eye

59. Biphasic force-frequency relation predicts primary cardiac events in patients with hypertrophic cardiomyopathy

60. Clinical and genetic characterization of patients with hypertrophic cardiomyopathy and right atrial enlargement

61. Clinical recommendations of cardiac magnetic resonance, Part II: Inflammatory and congenital heart disease, cardiomyopathies and cardiac tumors: A position paper of the working group 'Applicazioni della Risonanza Magnetica' of the Italian Society of Cardiology

62. Implantation of a Left Ventricular Assist Device for Danon Cardiomyopathy

Cochrane

Record #1 of 1

ID: DARE-12012021555

AU: Green JJ

AU: Berger JS

AU: Kramer CM

AU: Salerno M

TI: Prognostic value of late gadolinium enhancement in clinical outcomes for hypertrophic cardiomyopathy (Provisional abstract)

SO: JACC: Cardiovascular Imaging

YR: 2012

VL: 5

NO: 4

PG: 370-377

2. Table 1, Agreement test for Newcastle–Ottawa Scale scores evaluated by reviewers

Table 1. Agreement test for Newcastle–Ottawa Scale scores evaluated by reviewers

author	Study design	NOS1	NOS2	NOS3
Olivotto et al.	prospective patients follow-up	7	7	
Olivotto et al.	prospective patients follow-up	8	7	7
Elliott et al.	retrospective cohort study	7	7	
Monserrat et al.	restrospective cohort study	8	8	
Maron et al.	multicenter cohort study	7	7	
Arteaga et al.	prospective patients follow-up	6	6	
Biagini et al.	prospective patients follow-up	6	7	6
Nistri et al.	registry study	8	8	
Nasermoaddeli et al.	A nationwide epidemiological study	6	6	
Losi et al.	patients follow-up	8	8	
Yang et al.	prospective patients follow-up	8	8	
Gimeno et al.	retrospective cohort study	6	6	
Spirito et al.	registry study	6	6	
Dimitrow et al.	retrospective cohort study	5	6	5
Finocchiaro et al.	registry study	8	8	
Efthimiadis et al.	single-center cohort study	7	7	
Klarich et al.	retrospective cohort study	6	6	
Wang et al.	prospective patients follow-up	7	7	
Xiao et al.	retrospective cohort study	7	7	

* NOS = Newcastle–Ottawa Scale; NOS1=NOS score evaluated by reviewer1; NOS2=NOS score evaluated by reviewer2; NOS3= Disagreements between the reviewers were resolved by consensus with the third author. Agreement test for NOS score: Kappa=0.842, P=0.000.

Figure Legend:

1. Figure 1 Forest plots showing the prognostic value of NSVT for cardiac sudden death
2. Figure 2 Forest plots showing the prognostic value of FHSD for cardiovascular death
3. Figure 3 Forest plots showing the prognostic value of FHSD for cardiac sudden death
4. Figure 4 Combined effect analysis for NYHA
5. Figure 5 Combined effect analysis for Syncope
6. Figure 6 Combined effect analysis for MLVWT
7. Figure 7 Begg's funnel plot to assess the degree of publication bias. Circles in black color represent the nine original studies included in the meta-analysis. Eggers test for NYHA class III/IV of cardiovascular death was not significant (p = 0.095). HR: hazard ratio.

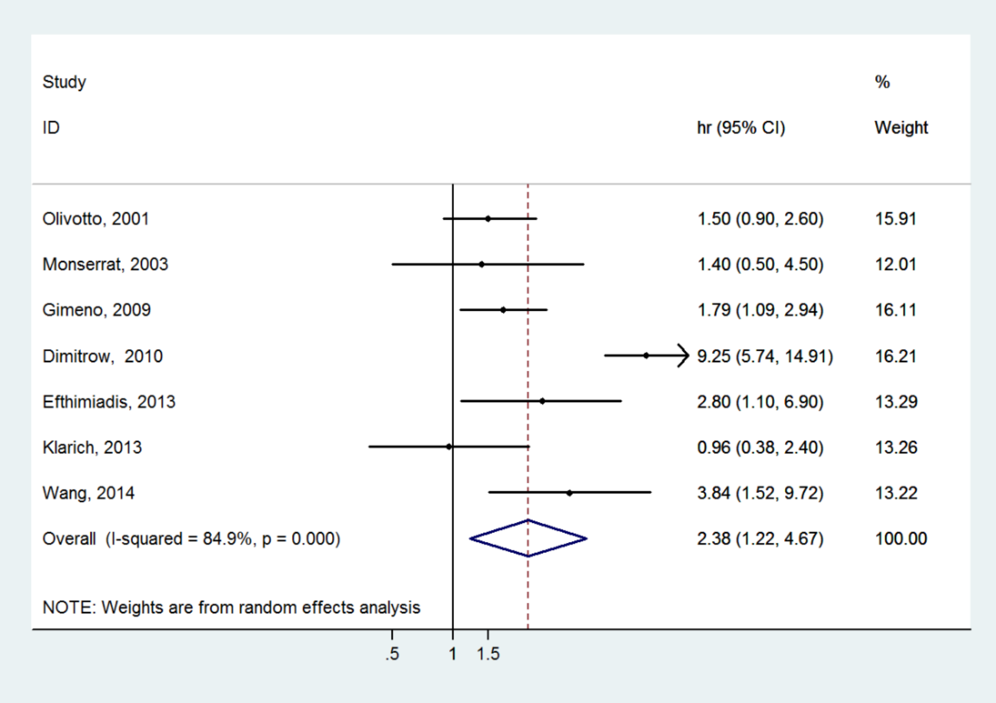

Figure 1 Forest plots showing the prognostic value of FHSD for cardiovascular death

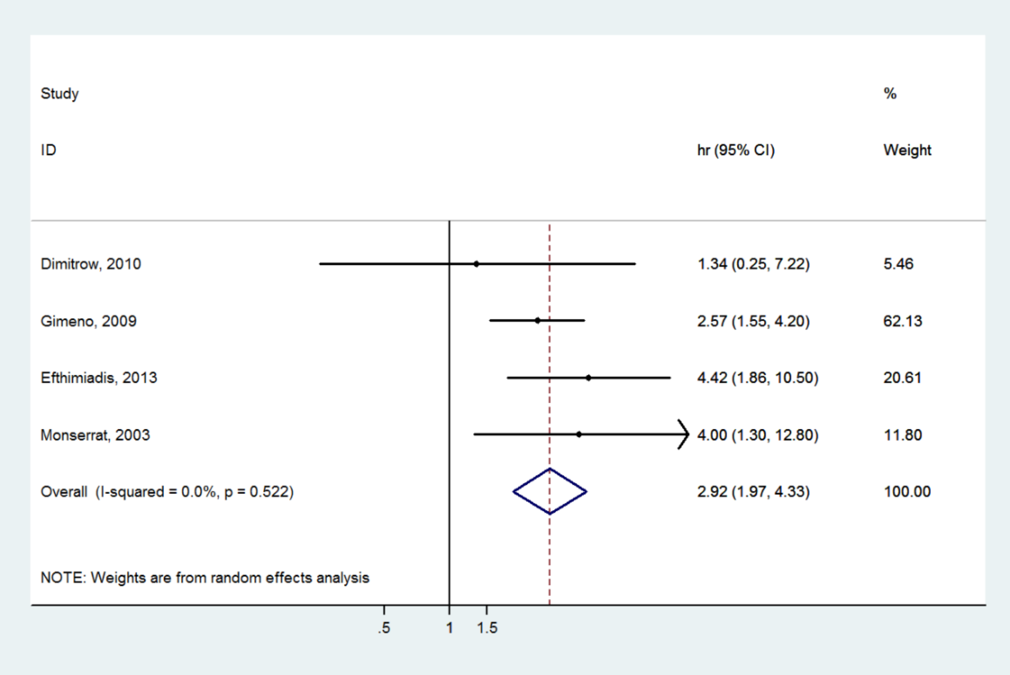

Figure 2 Forest plots showing the prognostic value of NSVT for cardiac sudden death

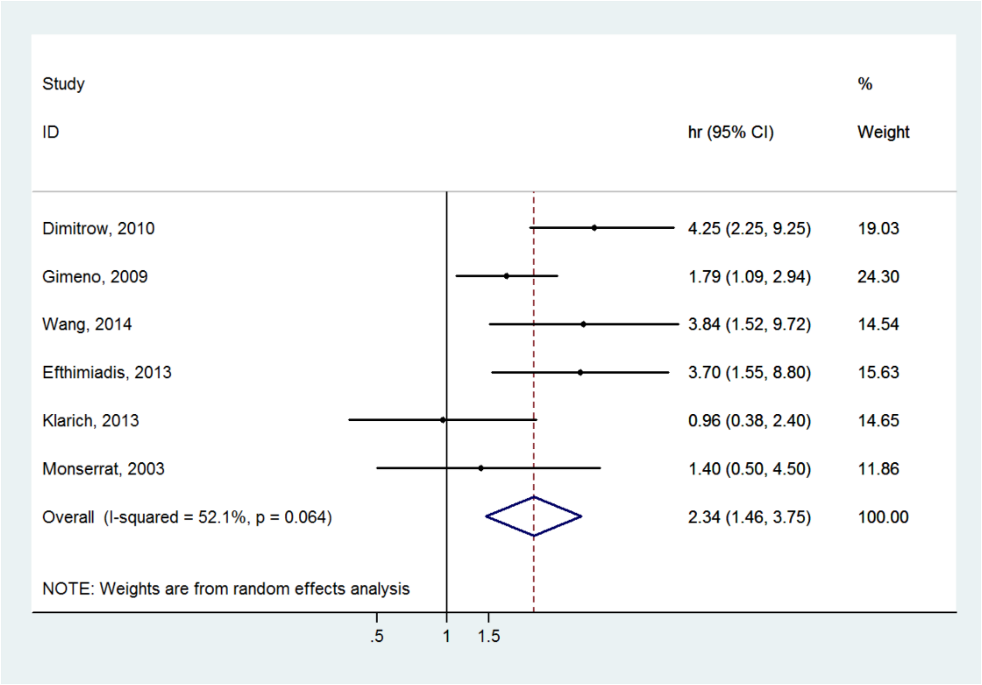

Figure 3 Forest plots showing the prognostic value of FHSD for cardiac sudden death

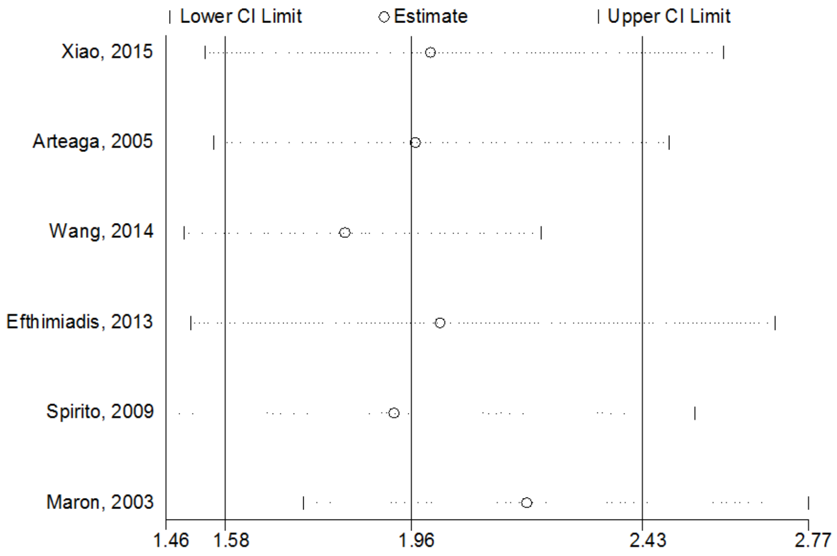

Figure 4 Combined effect analysis for NYHA

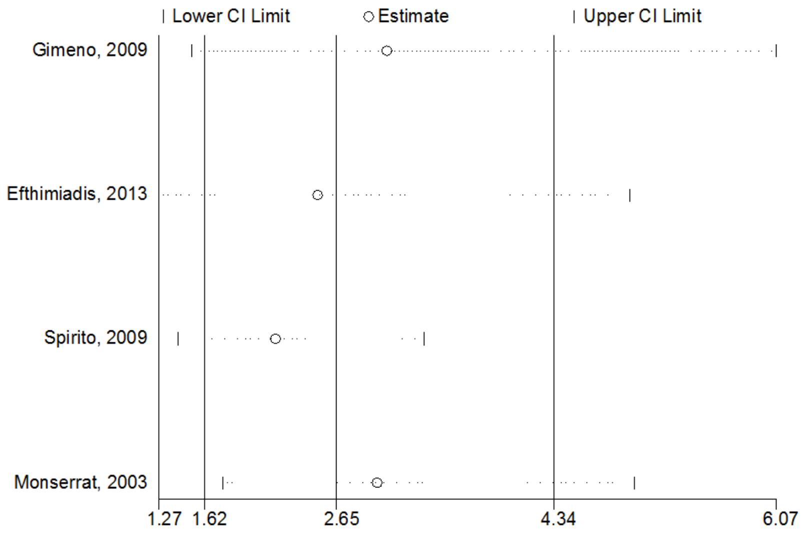

Figure 5 Combined effect analysis for Syncope

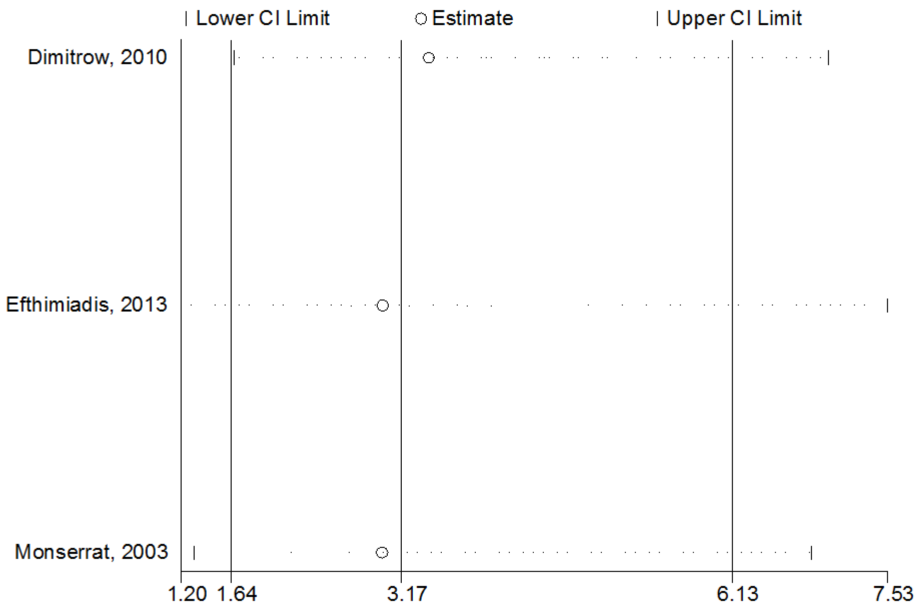

Figure 6 Combined effect analysis for MLVWT

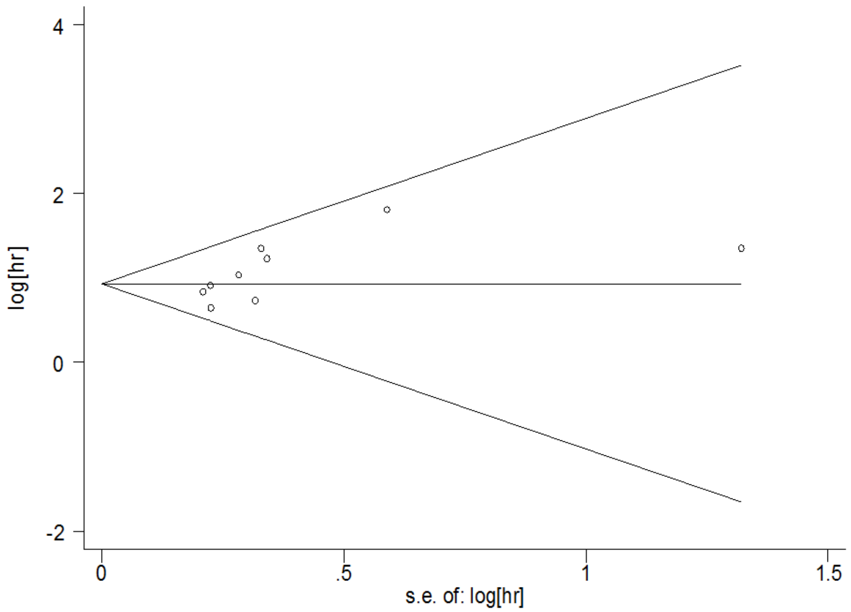

Figure 7 Begg’s funnel plot to assess the degree of publication bias.

Circles in black color represent the nine original studies included in the meta-analysis. Eggers test for NYHA class III/IV of cardiovascular death was not significant (p = 0.093). HR: hazard ratio.
